# BRAF inhibitor resistance in melanoma: from resistance mechanisms to therapeutic innovations

**DOI:** 10.1186/s43556-026-00425-4

**Published:** 2026-03-11

**Authors:** Yan Shang, Tingping Cao, Junyan Li, Juan Li, Lingnan Zhang, Qiqi Ma, Lanyan Feng, Hailong Zhao

**Affiliations:** 1 Department of Pathophysiology, School of Preclinical Medicine, Zunyi Medical University, Zunyi Guizhou, 563003 China; 2 Department of Medical Genetics, School of Preclinical Medicine, Zunyi Medical University, Zunyi Guizhou, 563003 China; 3 Guizhou Sinan County People’s Hospital, Tongren Guizhou, 565199 China; 4 Key Laboratory of Basic Pharmacology of Ministry of Education, Zunyi Medical University, Zunyi Guizhou, 563003 China; 5 School of First Clinical Medicine, Zunyi Medical University, Zunyi Guizhou, 563003 China

**Keywords:** Melanoma, BRAF inhibitor, Drug resistance, Targeted therapy, Precision medicine

## Abstract

BRAF inhibitors (BRAFi) have transformed the treatment of *BRAF* mutant melanoma, but inherent and acquired resistance remains a major barrier to curative outcomes. Resistance arises from interconnected mechanisms: genetic alterations reactivating the MAPK pathway or bypass cascades (e.g., PI3K/AKT/RTK), epigenetic modulation, metabolic reprogramming, and the tumor microenvironment (TME) remodeling. Despite extensive research into these mechanisms, a cohesive framework linking each resistance module to targeted therapeutic strategies is lacking. This review systematically categorizes resistance into intrinsic and acquired subtypes: intrinsic resistance is driven by constitutive molecular traits of *BRAF* mutant melanoma (e.g., persistent MAPK activation, baseline PI3K/AKT hyperactivity), while acquired resistance emerges via therapeutic pressure-induced genetic mutations, epigenetic shifts, metabolic reprogramming, or TME modifications. For each identified resistance mechanism, we provide a detailed examination of corresponding therapeutic advancements. These encompass the development of next-generation BRAFi, strategically designed combination therapies, epigenetic modulators, immunotherapeutic approaches, and RNA-based therapeutic agents. Furthermore, we underscore the pivotal role of state-of-the-art technologies, such as liquid biopsies, single-cell multi-omics analyses, and artificial intelligence, in facilitating precise resistance monitoring and personalized therapy selection. By integrating these insights, we present a structured, translationally focused framework to guide basic research and clinical decision-making, ultimately advancing precision salvage therapy and trials aimed at preventing or overcoming BRAFi resistance.

## Introduction

Metastatic melanoma has historically represented a formidable therapeutic challenge, characterized by its aggressive biology and refractoriness to conventional chemotherapy, which resulted in a dismal prognosis for affected individuals [1, 2]. The discovery that activating *BRAF* mutations are harbored by approximately 50% of cutaneous melanomas, alongside their prevalence in 40% of papillary thyroid carcinomas and 10% of colorectal cancers, established *BRAF*-mutant malignancies as the inaugural paradigm for genotype-directed therapy in solid tumors [3–5]. This foundational insight catalyzed the development and widespread clinical deployment of selective BRAF inhibitors (BRAFi) for the management of *BRAF*-mutated melanoma [6, 7], thyroid carcinoma [8, 9], and colorectal carcinoma [10, 11]. The regulatory approval of vemurafenib, followed by dabrafenib and encorafenib, has cemented the combination of BRAF and MEK inhibition (BRAFi ± MEKi) as the standard-of-care for metastatic disease, yielding objective response rates exceeding 60% and conferring the first substantial extension in overall survival [12, 13]. Beyond their canonical on-target effects, agents such as vemurafenib, dabrafenib, and binimetinib have been shown to exert ancillary immunomodulatory functions that contribute to prolonged progression-free survival in melanoma [14–16]. Furthermore, structural elucidation of the mechanisms by which RAS-driven paradoxical MAPK activation can compromise first-generation inhibitors has propelled the rational design of next-generation allosteric inhibitors and quinazoline-based analogues with enhanced potency and selectivity [17].

Notwithstanding these transformative therapeutic advances, the attainment of durable clinical benefit remains an unmet need for a significant proportion of patients [18]. Disease progression, driven by the emergence of therapeutic resistance, typically ensues within 6–12 months of treatment initiation [19, 20]. This pervasive limitation arises from a complex interplay of intrinsic tumor properties and adaptive, acquired resistance mechanisms forged under the selective pressure of therapy, encompassing genomic instability, epigenetic plasticity, metabolic reprogramming, and dynamic crosstalk with the tumor microenvironment. A bibliometric analysis spanning the period from 2003 To 2024 underscores the exponential expansion of research in this domain, with leading contributions from the United States, China, and Italy, and reveals a strategic pivot in scientific inquiry from the initial characterization of *BRAF* mutations and MAPK pathway reactivation toward the exploration of synergistic immunotherapy combinations, novel therapeutic regimens, and the roles of non-apoptotic cell death modalities [21]. To synthesize this rapidly evolving and multifaceted body of knowledge, we present this comprehensive review.

This review aims to develop a unified, translationally oriented framework that not only maps the molecular architecture of BRAFi resistance but also directly couples each resistance mechanism to its rationally derived therapeutic strategy (Table 1). We commence by delineating the molecular underpinnings of intrinsic resistance, which is predicated upon constitutive features of the tumor that confer baseline insensitivity. Subsequently, we deconstruct acquired resistance into four interconnected and non-mutually exclusive dimensions: genetic alterations such as secondary mutations and bypass track activation; non-genetic reprogramming mediated through epigenetic modifications and transcriptional network rewiring; adaptive metabolic shifts that support tumor survival under drug pressure; and reciprocal remodeling of the tumor microenvironment that fosters a protective niche. For each of these mechanistic categories, we provide a detailed appraisal of emerging salvage strategies, ranging from advanced targeted combinations and novel small-molecule inhibitors to epigenetic modulators, metabolic interventions, and innovative immunotherapeutic approaches. Finally, we highlight the pivotal role of cutting-edge technologies including liquid biopsy-based circulating free DNA (cfDNA) analysis, single-cell multi-omics, and artificial intelligence in enabling real-time, dynamic monitoring of resistance evolution to inform precision-guided therapeutic decision-making. Collectively, these insights underscore that overcoming the profound heterogeneity and dynamic adaptability of BRAFi resistance requires a therapeutic paradigm that is simultaneously multi-targeted, mechanism-guided, and responsive to real-time tumor evolution.

**Table 1:** BRAFi resistance in melanoma: genetic, epigenetic, metabolic, and microenvironmental mechanisms with targeted and emerging therapies

**Category**	**Core Mechanisms**	Ref
Intrinsic Resistance	• *BRAF*-V600E keeps MAPK “on” • ↑PI3K/AKT & RTKs (IGF-1R, EGFR)	[22–25]
Acquired – genetic	• ↑Secondary BRAF/MEK/NRAS • ↑YAP/TAZ • ↓STAG2/3 • ↑TERT-promoter	[24, 26–28]
Acquired – non-genetic	• ↑DNA methylation (PDE4D • ↑cGAS-STING) • ↑SAMMSON • ↑miR-211 • ↑Glutaminolysis • ↑Polyamine	[29–33]
Tumor micro-environment	• ↑CAF IL-6/TNF-α/M-CSF • ↑Lipid-loaded fibroblasts • ↑Soluble CD73	[34–37]
Targeted combinations	• BRAFi + MEKi ± PI3Ki • PROTACs • PARPi • FAKi • Hsp90i	[38–41]
Epigenetic/metabolic	• DNMTi, EZH2i • PDE4Di • Glutaminase • CSE • SREBP blockade	[42–47]
Immuno-combinations	• BRAF/MEK + PD-1 blockade • HDACi or STAT3i to restore T-cell influx	[48–52]
Emerging technologies	• ↑cfDNA tracks *BRAF*-V600E • scRNA-seq maps resistant clones • AI predicts combos; early imaging guides switch	[53–59]

## Intrinsic resistance to BRAF inhibitors in melanoma: molecular basis and clinical implications

### MAPK signaling pathway dysregulation in *BRAF* mutant melanoma

*BRAF* encodes a serine/threonine kinase, the core activator of the MAPK/ERK pathway [1, 60]. It is central to the regulation of cell proliferation, differentiation, migration and survival [61, 62]. Mutations in the *BRAF* gene (predominantly in exons 11 and 15) occur in ~ 7% of all cancers, with the V600E variant (exon 15) being the most common [63]. Mutations in the *BRAF* gene lead to the constitutive activation of the BRAF protein, driving normal cells to undergo continuous division and potentially culminating in oncogenesis [64]. *BRAF* mutations are in ~ 7% of cancers, including 100% hairy cell leukemia, 50–60% melanoma, 30–50% papillary thyroid cancer, 10–20% colorectal cancer, and 3–5% NSCLC [65]. BRAF V600E renders the kinase RAS-independent, constitutively activating downstream RAS/RAF/MEK/ERK signaling and promoting tumor proliferation, invasion, and metastasis [66].

Monomeric *BRAF* V600E, the canonical class I mutant, operates independently of RAS and drives high-level ERK signaling [67]. Consequently, in *BRAF* V600E-mutant tumors, *BRAF V600E* exists predominantly as a drug-sensitive monomer. Current RAF inhibitors selectively target *BRAF* monomers, exhibiting substantially weaker inhibition of RAF dimers [22]. BRAF V600E alone remains inhibitor-sensitive, but resistance arises when elevated RAS-GTP drives mutant kinase dimerization with wild-type BRAF or CRAF [68]. The formation of *BRAF* mutant dimers represents another significant mechanism underlying aberrant signaling pathway transduction in the BRAF pathway. Elevated BRAF dimerization not only confers increased drug resistance but also operates independently of upstream RAS activation, perpetuating downstream RAF/MEK/ERK signaling [69]. Apart from BRAF dimerization, RAS gene mutations can likewise induce aberrant activation of the BRAF signaling pathway. Activated RAS mutations can coexist with hypoactive or kinase-inactivated *BRAF* mutants. These *BRAF* mutants, whether hypoactive or kinase-inactivated, activate the MEK/ERK signaling pathway in a RAS-dependent manner, promoting a malignant phenotype in tumor cells [23]. CEP55 drives acral melanoma progression via MAPK activation, causing BRAFi resistance, yet its overexpression predicts favorable immunotherapy responses, making it a therapeutic target [70]. In summary, *BRAF* gene mutations drive tumor progression via two mechanisms: independent activation (single mutations, mutant dimers) and RAS co-mutation, both mechanisms promote a malignant tumor phenotype. Consequently, the clinical investigation and application of BRAFi can attenuate the malignant progression of tumors by inhibiting the aberrant activation of BRAF-mediated signaling pathways.

### Clinical efficacy, heterogeneity, and limitations of BRAF inhibitors in *BRAF* mutant melanoma

BRAFi rechallenge after progression yielded significant survival benefit in a BRAF V600 melanoma patient, suggesting that loss of evolutionary advantage in resistant clones restores cancer cell sensitivity to therapy [71]. The current clinical use of BRAFi is predominantly in combination with MEK inhibitors (MEKi) [72–74]. Dabrafenib and trametinib became the first FDA-approved targeted therapy on March 16, 2023, for systemic treatment of pediatric BRAF V600E low-grade glioma [75]. In clinical practice, BRAFi have demonstrated strong efficacy and a tolerable safety profile for BRAF V600E–mutant non-small cell lung cancer and glioma, with side effects that remain manageable [76–78]. These results have led the FDA to grant dabrafenib breakthrough therapy designation [79, 80].

Treating BRAF-mutant tumors effectively calls for either BRAFi alone or combined with MEKi, as demonstrated by SECOMBIT's strategy of using sequential combinations followed by immunotherapy maintenance [81, 82]. Long-term survival is possible for some BRAF-mutant patients. Encorafenib plus binimetinib, approved by the FDA in 2023 for BRAF V600E metastatic NSCLC in adults, illustrates this benefit [83, 84]. Transcriptome analysis reveals FAK activation mediates BRAF/MEKi resistance in melanoma, and FAKi plus avutometinib overcomes this by disrupting MAPK-RhoA-FAK-AKT feedback [38]. However, resistance to BRAFi (monotherapy or combination with MEK inhibitors) remains a significant clinical challenge despite their substantial therapeutic efficacy [85, 86]. For instance, vemurafenib and PLX8394 show off-target effects causing paradoxical endothelial MAPK activation and barrier impairment, unlike dabrafenib or encorafenib. Understanding these off-target effects is crucial for optimizing therapeutic regimens and minimizing adverse events in *BRAF* mutant melanoma treatment [87]. Vemurafenib and dabrafenib for V600E melanoma downregulate oncogenic RIPK4, a BRAF homolog, via direct binding and ERK1/2 modulation, enhancing efficacy and circumventing resistance via off-target effects [88]. BRAFi resistance leaves melanoma cells with heightened replication stress and an S-phase dependency that can be exploited to overcome MAPK pathway resistance [89]. In summary, BRAFi have made significant clinical headway against *BRAF V600E* solid tumors. Recent molecular insights from Asian melanoma populations also point toward the necessity of tailored treatments, especially for acral and mucosal forms [90]. However, the growing problem of resistance alongside ongoing drug development efforts has pushed BRAFi resistance reversal to the forefront, and scientists are now working hard to find answers.

Intrinsic resistance stems from the inherent molecular makeup of *BRAF* mutant melanoma, where constant MAPK activation and baseline bypass signaling create initial drug insensitivity. Most patients who initially respond to BRAFi still progress within 6 to 12 months as acquired resistance develops, an evolutionary adaptation driven by treatment pressure. Tumors remodel their genetic circuits, epigenetic states, metabolic pathways, or microenvironment to escape BRAF blockade, layering these adaptations atop intrinsic traits to form a complex defense that demands thorough mechanistic investigation.

## Acquired resistance to BRAF inhibitors: adaptive evolution via genetic, epigenetic, metabolic and the tumor microenvironment remodeling

Acquired resistance develops when tumors initially sensitive to BRAFi lose response during treatment, driven by genetic mutations, epigenetic shifts, metabolic rewiring, or changes in the tumor microenvironment (TME). This differs from intrinsic resistance, which exists prior to therapy. Instead, acquired resistance emerges under the selective pressure of BRAFi itself, allowing drug-tolerant cell populations to survive and expand. We organize these adaptive processes into four interconnected categories: genetic alterations, epigenetic dysregulation, metabolic reprogramming, and tumor microenvironment remodeling.

### Genetic mechanisms of adaptive escape

#### *BRAF* mutation specific genetic escape routes

**Fig. 1:**
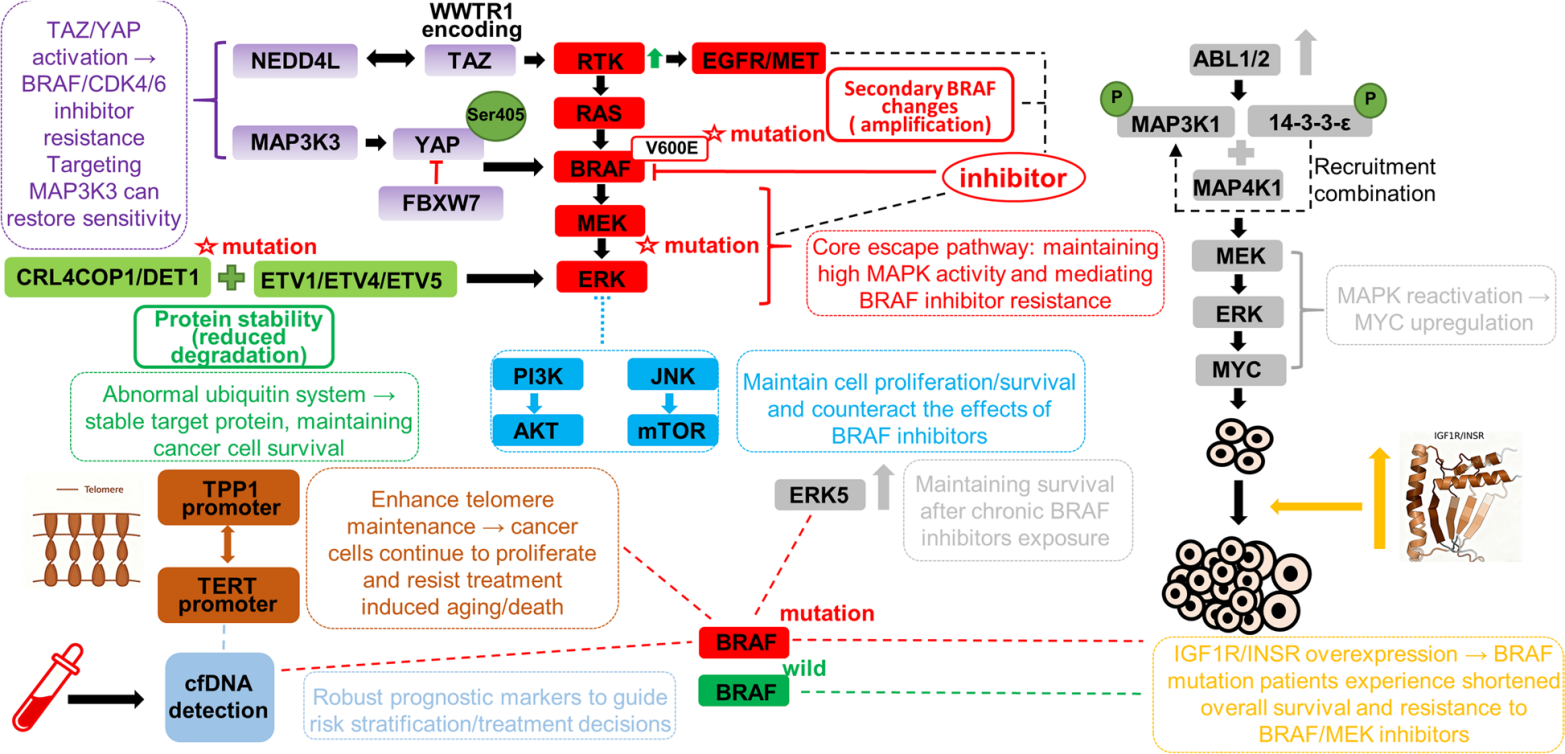
MAPK Reactivation and Parallel Bypass Pathways Confer BRAF Inhibitor Resistance in Melanoma

**Fig. 2:**
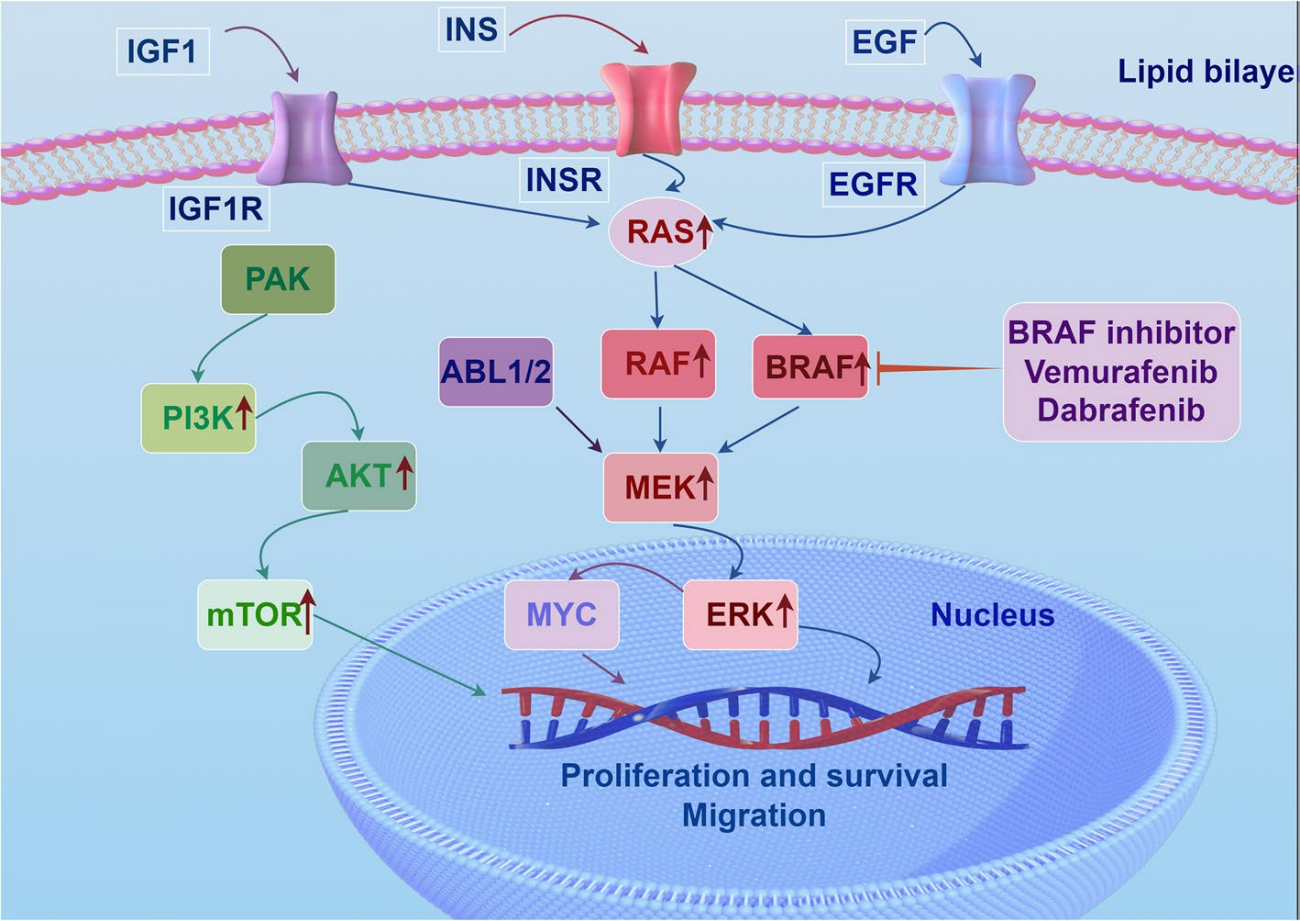
Gain-of-function BRAF mutations sustain oncogenic MAPK signaling, attenuating BRAF-inhibitor efficacy and driving intrinsic resistance; concomitant PI3K/AKT-pathway hyper-activation, together with amplified or ligand-stimulated IGF1R, INSR and EGFR, further potentiates resistance in *BRAF* mutant tumors

MAPK re-activation, achieved either by secondary BRAF alterations, RTK up-regulation or MEK/ERK mutations, remains the dominant escape route in *BRAF* mutant melanoma (Figs. 1 and 2). Down-stream rewiring via PI3K/AKT or JNK/mTOR is discussed only when genotype-specific [91–93]. Furthermore, a piggyBac screen identified BRAFi resistance genes in melanoma, revealing convergent activation of MAPK, PI3K-AKT, and notably Hippo pathways. Hippo effector TAZ (WWTR1) mediates BRAFi resistance through receptor tyrosine kinase regulation and NEDD4L interactions. This reveals potential targets to overcome resistance [94]. In BRAF V600E melanoma, acquired mutations in COP1 or DET1 stabilize ETV1, ETV4, and ETV5, driving resistance to MAPK inhibitors. This finding underscores the critical role of this E3 ligase in ERK-driven cancers [26]. MAP3K3 phosphorylates YAP at Ser405 to block FBXW7 degradation, sustaining YAP activity that drives BRAF/CDK4/6 inhibitor resistance, and targeting MAP3K3 restores sensitivity by lowering YAP [95]. Persistent BRAF V600E activity drives melanoma as a therapeutic target, causing oncogenic MAPK hyper-activation and intrinsic BRAFi resistance [27, 96]. *BRAF* mutations dictate metastatic potential and clinical outcome; therefore, precise molecular identification of BRAF alterations is indispensable for risk stratification and targeted therapeutic decision-making [97–100]. Heightened ABL1/2 phosphorylates MAP3K1 and 14–3-3-ε, recruiting MAP4K1 to reactivate MAP3K1-MEK-ERK and re-induce MYC. This MAPK pathway mechanism transcriptionally upregulates MYC to sustain melanoma resistance [101]. ERK5 signaling is amplified in dabrafenib-resistant BRAF V600E melanoma, sustaining proliferation and survival after chronic exposure, offering a target to dismantle acquired resistance [102]. Although WM9 and Hs294T survived BRAF/MEKi, they showed raised ERK/AKT/p38/JNK and altered receptors (EGFR/ErbB2/MET/PDGFRβ up, ErbB3 down). They also displayed elevated drug metabolism and EMT/CSC traits [103]. Of note, androgen receptor is elevated in BRAFi resistant melanoma and drives EGFR and SERPINE1 upregulation to maintain resistance [104]. MAPK alterations (KRAS/BRAF amplification, MEK1 mutations) confer RAF/EGFR or RAF/MEK resistance by sustaining pathway activity, promoting tumor drug resistance [105, 106]. TERT promoter mutations can act synergistically with TPP1 promoter mutations to enhance telomere maintenance and permanence in melanoma, allowing continued melanoma proliferation despite therapeutic intervention [107–110]. This mechanism operates independent of *BRAF* genotype and has been observed across all melanoma subtypes. Its overexpression reduces the sensitivity of *BRAF* mutant melanomas to BRAFi and MEKi, promoting the development of drug resistance in these tumors [111, 112]. Upregulation of IGF1R and INSR expression has been observed in *BRAF* mutant melanoma cell lines that are resistant to BRAFi and MEKi [24, 113]. Patients with *BRAF* mutations and elevated IGF1R and INSR expression exhibit poorer overall survival [24]. Because comparable IGF1R/INSR overexpression has also been documented in *BRAF* wild-type melanomas, this represents a pan-genotype bypass pathway. Genomic deletions producing BRAF splice variants conferring RAF-inhibitor resistance are actionable vulnerabilities, while cfDNA detection of BRAF/TERT alterations serves as a prognostic biomarker in melanoma [53, 114–116].

Furthermore, loss-of-function mutations in STAG2 or STAG3, adhesion protein complex subunits, occur in *BRAF* mutant melanoma [117, 118]. The loss of function, particularly of the STAG2 protein, impacts the expression of dual-specificity phosphatase 6 (DUSP6), resulting in resistance to BRAFi [118]. This leads to heightened resistance of melanoma to BRAFi and that is restricted to the *BRAF* mutant context [118]. *BRAF* mutant drug-resistant melanoma, the activation of p21-activated kinase (PAK) in cells that have acquired drug resistance reactivates the JNK and mTOR pathways. This bypasses the ERK pathway and inhibits apoptosis, promoting the proliferation of melanoma cells [119]. The convergence of MAPK (via ERK reactivation) and mTORC1 signaling pathways is crucial for driving cyclin D1 protein production, enabling cell cycle reentry and drug escape [120]. Because this PAK-driven bypass has also been observed in *NRAS*- and *NF1*-mutant melanomas, it qualifies as a genotype-independent resistance mechanism. The stem cell marker aldehyde dehydrogenase 1A1 (ALDH1A1) is overexpressed in *BRAF* mutant melanoma cells [121, 122]. This overexpression confers drug resistance and reprograms signaling from MEK/ERK to PI3K/AKT in *BRAF* mutant melanoma, promoting malignant progression [123]. Moreover, ALDH1A3-ACSS2 enzymatic coupling in melanoma links acetaldehyde metabolism to histone H3 acetylation of neural crest lineage genes, driving transcriptional heterogeneity and BRAFi resistance via chromatin remodeling [124]. ALDH2 acts as a detoxifying enzyme, and its reduced expression in metastatic melanoma predicts poorer survival. This downregulation also drives resistance to BRAF/MEKi, positioning ALDH2 as a potential therapeutic target [28]. EGFR pathway activation confers BRAFi resistance in vitro and in vivo and activates RTK and AKT in EGFR-activated melanoma cells [125, 126]. This phenotype occurs in both BRAF mutant and wild-type melanomas, indicating a pan-genotype bypass mechanism causing uninhibited proliferation and increased malignant progression [25, 127, 128]. PTRF has been identified as a key contributor to acquired drug resistance in melanoma by upregulating EGFR expression, leading to reduced sensitivity to BRAFi and their combinations with MEKi [129]. Building on recent evidence, in vemurafenib-resistant melanoma cells the EGF/EGFR-YAP1/TEAD2 axis drives nuclear accumulation of YAP1, which transcriptionally up-regulates the endoplasmic reticulum Ca^2^⁺ sensor STIM1, amplifies store-operated calcium entry and fuels both tumor progression and continued drug resistance [130].

Furthermore, the ATP-Binding Cassette (ABC) transporter family is a major factor mediating drug resistance in tumors [131–133]. It facilitates drug efflux, preventing efficient intracellular drug accumulation and promoting resistance in *BRAF* mutant melanoma cells. This mechanism is observed in all melanoma subtypes and therefore constitutes a genotype-independent contributor to resistance in *BRAF* mutant melanoma cells [134, 135]. In the presence of functional ABCG2, the inhibitory effect of vemurafenib on *BRAF V600E*-mutant melanoma cells is attenuated. The tolerance of tumor cells to the drug is enhanced. These findings suggest that ABCG2 confers resistance to BRAFi in melanoma cells [136, 137]. Vemurafenib enhances ABCB1-mediated drug efflux, increasing resistance and decreasing sensitivity to vemurafenib in *BRAF* mutant melanomas, because ABCB1 up-regulation has also been reported in *BRAF* wild-type melanomas treated with chemotherapy or immunotherapy, this represents a pan-genotype efflux-based resistance mechanism [138]. In *BRAF V600*-mutant melanoma brain metastases, intrinsic vemurafenib resistance is driven by blood–brain barrier P-gp and BCRP limiting drug entry. As shown by superior responses in ABCB1a/b, ABCG2 knockout mice, whereas acquired resistance emerges rapidly and independently of pharmacokinetics or target blockade [139].UBE3C, identified as an E3 ubiquitin ligase for mutant *BRAF V600E*, facilitates its ubiquitination and degradation, suggesting a novel strategy to overcome secondary resistance to BRAFi like Vemurafenib by targeting UBE3C [140]. Therefore, studies targeting the ABC transporter family represent a potential approach to elucidate the mechanisms of acquired resistance to vemurafenib in *BRAF V600E*-mutant melanoma cells.

#### Pan-genotype genetic drivers of cross-resistance

Although our focus is on resistance in *BRAF* mutant tumors, melanoma cells carrying *NRAS, NF1, KIT* mutations or triple-WT also exhibit intrinsic or acquired insensitivity to BRAF-targeted therapy [141–145]. We outline these pan-genotypic mechanisms and identify the relationship between these genes and the MAPK signaling pathway, thereby justifying alternative treatment strategies. Patients with *NRAS* mutant tumors lack a druggable *BRAF* mutation and show high intrinsic and acquired resistance to MEKi, so they do not benefit from these therapies [146]. In *NRAS*-mutant melanoma, the MAPK signaling pathway plays a negative feedback role in which ERK is hyperactivated and EGR1 is overexpressed, and this mechanism is regulated by eIF4F [96, 146, 147]. Meanwhile, using a *BRAF V600E* melanoma patient derived tumor xenograft model, researchers uncovered new acquired resistance drivers GPR39, CD27, SLC15A3, IFI27, PDGFA, and ABCB1, which act via immune modulation, microenvironment shifts or drug efflux and represent fresh targets to combat BRAFi resistance [148]. Furthermore, the emergence of melanoma resistance is associated with *KIT* mutations and also occurs independently of secondary *KIT* mutations through reactivation of the MAPK and PI3K/AKT pathways [149]. Updates on BRAFi from 2018–2023 show that *RAC1* mutations, *PTEN* loss, and *NF1/CCND1* alterations fuel acquired resistance across cancers, driving the need for next-generation compounds with tighter structure–activity profiles and fewer off-target effects [150]. *NRAS*, *NF1*, *KIT* mutations and triple wild type tumors can mediate resistance through shared mechanisms such as reactivation of the MAPK or PI3K/AKT pathways, providing a theoretical basis for subsequent combination targeted strategies.

*BRAF*-mutant melanoma typically evades therapy through several mutation-restricted mechanisms, including MAPK pathway reactivation, PI3K/AKT signaling rewiring, upregulation of ABC efflux pumps, and exploitation of ALDH1A1 or STAG2 loss. Concurrently, alterations in RAS, KIT, or triple wild-type tumors provide ancillary reinforcement of MAPK and PI3K signaling across diverse genetic contexts. Therefore, effective strategies should prioritize concurrent blockade of MAPK and PI3K axes, pharmacologic inhibition of ABC transporters, and targeted modulation of epigenetic and microenvironmental cues to surmount resistance and prolong clinical benefit. Beyond genetic alterations, non-genetic mechanisms marked by reversible epigenetic modifications and transcriptional reprogramming are equally crucial in mediating acquired resistance. These processes frequently collaborate with genetic drivers to enhance tumor cell survival under therapeutic pressure from BRAFi.

### Non-genetic resistance: epigenetic and transcriptional reprogramming

#### DNA methylation-mediated resistance

**Fig. 3:**
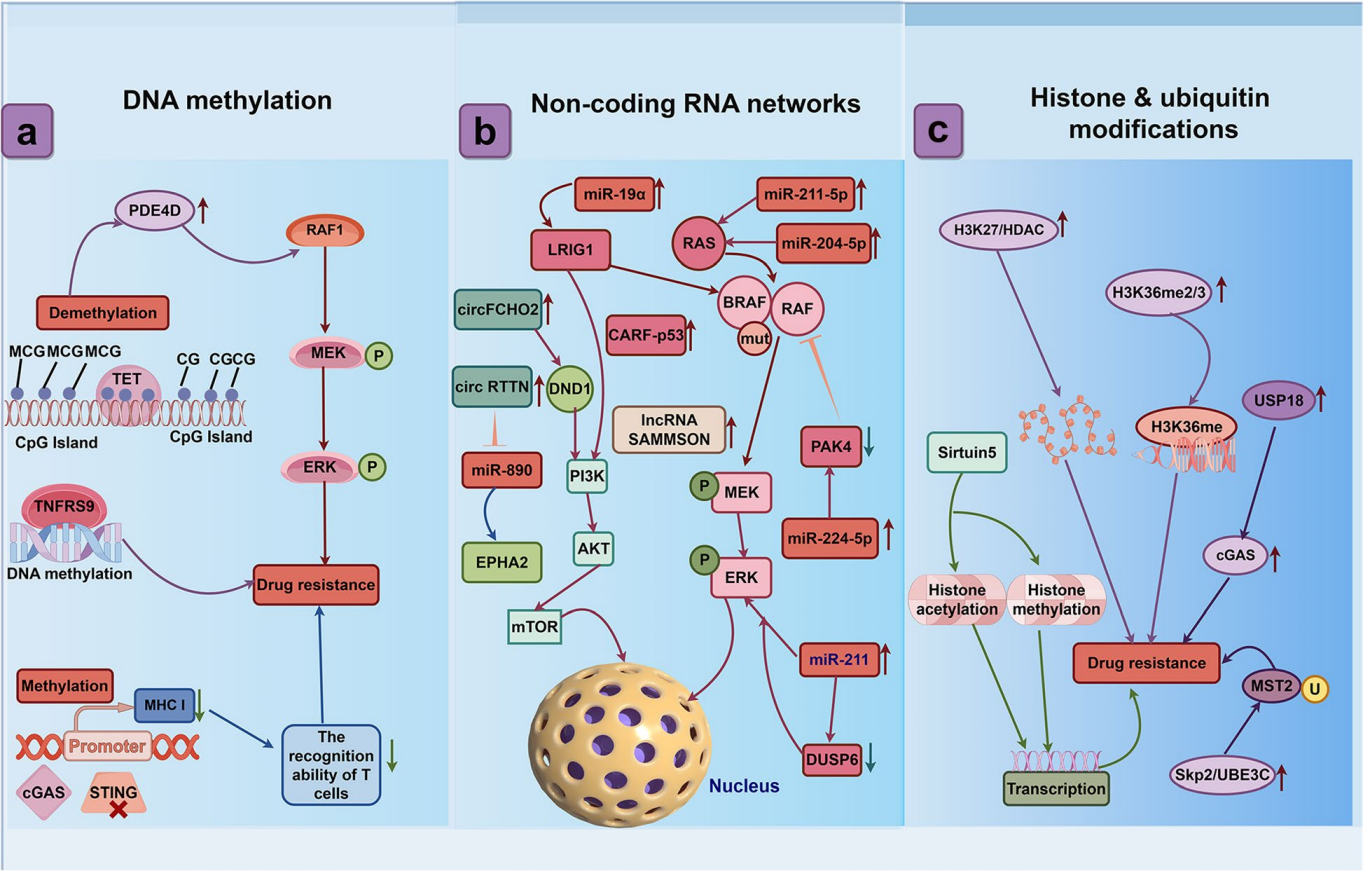
Epigenetic Drivers of BRAF-Inhibitor Resistance in Melanoma: DNA Methylation, Non-coding RNAs, and Histone/Ubiquitin Modifications. **a** DNA Methylation Rewiring in BRAF Mutant Melanoma: from PDE4D RAF1 Reactivation and cGAS STING Immune Escape to TNFRSF9 SHOX2 Liquid Biopsy Biomarkers for Epigenetic Combination Therapy. **b** Noncoding RNAs comprising microRNAs, circular RNAs and long non-coding RNAs exhibit crosstalk and independently enhance PI3K/AKT and MAPK signaling, thereby driving BRAF-inhibitor resistance in melanoma cells. **c** Post-translational histone and ubiquitin modifications that confer BRAF inhibitor resistance in melanoma

Epigenetic profiling has revealed distinct BRAFi resistance mechanisms, highlighting the critical role of epigenetic plasticity during acquired resistance [29]. Epigenetic alterations contributing to intrinsic resistance mechanisms to BRAFi in *BRAF* mutant melanoma cells include DNA methylation, noncoding RNA regulation and protein modification [30, 74, 151, 152]. Overexpression of DNA methyltransferase is closely associated with the malignant progression of *BRAF* mutant melanoma cells [153–155]. It is not only overexpressed in BRAFi-resistant *BRAF* mutant melanomas but also highly expressed in metastatic melanomas regardless of BRAF status, representing a pan-genotype epigenetic driver [31, 156]. Moreover, demethylation of the CpG island upstream of the phosphodiesterase 4D (*PDE4D*) gene promoter can lead to increased PDE4D expression in drug-resistant melanoma cell lines. This occurs preferentially in *BRAF* mutant tumors and enhances RAF1 activation, contributing to increased resistance to BRAFi in that sp-ecific context [42]. DNA methylation remodeling is a key driver of melanoma immune escape [157, 158]. Promoter hypermethylation of cGAS and STING impairs STING signaling, downregulates MHC class I expression, and blunts cytotoxic T cell recognition and killing, thereby fostering immune evasion and resistance [159]. Hypermethylation of circulating cell free SHOX2 DNA in plasma correlates with anti PD-1 response and can serve as an early companion biomarker for melanoma patients receiving anti PD-1 therapy [54]. Elevated DNA methylation of the T cell costimulatory receptor TNFRSF9 is associated with shorter progression free survival, whereas high TNFRSF9 mRNA expression together with low TNFRSF9 methylation predicts longer overall survival, providing a rationale for targeting TNFRSF9 DNA methylation in immunotherapy [160]. Therefore, DNA methylation may represent a promising target for epigenetic and targeted combination therapies (Fig. 3a).

#### Non-coding RNA networks

Various non-coding RNAs, from miRNAs to lncRNAs and circRNAs, influence how BRAF mutant melanoma develops and resists BRAFi by regulating Wnt and MAPK pathways, marking them as valuable for both detection and treatment [161]. Resistance to BRAFi mediated by ncRNAs operates through distinct mechanisms that align with three functional themes, each associated with a particular class of ncRNA.

Altered miRNA expression shows a tight association with BRAFi resistance emergence in melanomas carrying *BRAF* mutations. These miRNAs tune essential signaling pathways and gene programs that keep tumor cells alive and dividing when faced with BRAFi treatment pressure [162–166]. For instance, pigmented melanoma cells resist BRAFi by upregulating miR-192-5p, miR-211-5p, GPR143, and OCA2, which drive melanosome maturation and trafficking, suggesting that pigmentation-based resistance mechanisms represent potential combination targets [167]. Furthermore, certain miRNAs facilitate therapeutic resistance by reactivating the MAPK/PI3K signaling axis. Notably, miR-19a, highly expressed in *BRAF* mutant melanoma cells, promotes resistance to BRAFi by targeting immunoglobulin-like domain-containing protein 1 (LRIG1), a mechanism specific to the *BRAF* mutant context [168]. Additionally, miR-19a is abundantly present in exosomes secreted by BRAFi-resistant melanoma cells, which in turn propagate drug resistance to other BRAFi-sensitive melanoma cells [168]. Conversely, let-7a miRNA enhances the synergistic apoptotic effects of dabrafenib and trametinib combination therapy in melanoma cells by targeting caspase-3 [169]. The expression of miR-211 augments melanoma cell resistance to vemurafenib and cobimetinib, a resistance associated with elevated ERK5 phosphorylation, as miR-211 directly inhibits DUSP6, thereby sustaining ERK5 activation to promote proliferation and counteract BRAFi [170, 171]. Moreover, ectopic expression of miR-204-5p and miR-211-5p in untreated human melanoma cells confers resistance to vemurafenib and enhances in vivo tumor growth, with their combined overexpression persistently activating Ras and inducing MAPK upregulation upon vemurafenib exposure, specifically within *BRAF* mutant melanomas [162]. It is noteworthy that vemurafenib efficacy in melanoma is constrained, and drug resistance arises only when the basal level of miR-211 surpasses that of miR-204, an effect mediated by MITF-dependent pro-pigmentation activity [172]. Recent studies have also highlighted the upregulated expression of specific miRNAs, such as miR-4443 and miR-4488, in drug-resistant melanomas, which promote migratory and invasive phenotypes by downregulating the intermediate filament protein nestin, further exacerbating drug resistance [173]. Conversely, miR-224-5p acts as a tumor suppressor in melanoma by directly targeting PAK4 to inhibit the CRAF/MEK/ERK pathway, thereby reversing acquired resistance to BRAFi [32]. Thus, identifying miRNAs that confer resistance to BRAFi in drug-resistant melanoma could pave the way for mechanism-based strategies to mitigate resistance and enhance clinical outcomes.

The regulation of signal transduction pathways is intricately linked to the development of adaptive drug resistance in *BRAF* mutant melanomas, with lncRNAs playing a central role. Therapeutic pressure exerted by BRAFi induces alterations in signaling cascades, leading to the activation of compensatory mechanisms that enable tumor cells to evade the inhibitory effects of the drug. These adaptive changes are crucial for tumor cell survival and contribute significantly to the emergence of resistance. LncRNAs are essential for the growth and survival of human *BRAF* mutant melanoma cells. However, overexpression of certain lncRNAs can specifically mediate resistance of *BRAF* mutant melanoma to BRAFi within the mutant setting. For instance, overexpression of the lncRNA SAMMSON enhances resistance to RAF inhibitors in *BRAF* mutant melanoma by modulating CARF-p53 signaling, thereby contributing to adaptive resistance [174, 175]. This highlights the importance of lncRNAs in fine-tuning cellular responses to BRAFi therapy and their potential as therapeutic targets.

Circular RNAs (circRNAs) exhibit significant upregulation in drug-resistant melanoma cells, where they serve as critical signaling modulators that attenuate the sensitivity of melanoma to BRAFi. Mechanistically, circRNAs function as molecular sponges, sequestering multiple microRNAs (miRNAs) and thereby activating various canonical signaling pathways implicated in drug resistance [176–179]. Notably, circFCHO2, an elevated circRNA, directly interacts with DND1 to activate PI3K/AKT signaling, thereby enhancing melanoma aggressiveness in vitro [180]. This underscores the multifaceted role of circRNAs in driving tumor malignancy and drug resistance. In a broader context of ncRNA involvement, the overexpressed lncRNA U73166 has been implicated in mediating RNA processing, cell invasion, and the induction of a more aggressive tumor phenotype, as well as conferring resistance to vemurafenib in *BRAF*-mutant melanoma cells. However, to maintain a strict focus on circRNAs, it is pertinent to highlight that other, yet-to-be-fully-characterized circRNAs likely play analogous roles in melanoma resistance [181]. The identification and targeted inhibition of circRNAs that confer resistance to BRAFi in drug-resistant melanoma cells represent a novel and promising therapeutic strategy to overcome resistance and restore melanoma sensitivity to BRAFi (Fig. 3b).

#### Histone and ubiquitin modifications

Protein modification plays a crucial regulatory role in tumor cells, with altered protein conformations participating in cell signaling, metabolic regulation and the manifestation of malignant cellular phenotypes [182–184] (Fig. 3c). Conversion of histone H3 lysine 27 methylation to acetylation opens chromatin and drives BRAFi resistance in mutant melanoma cells [185]. In these resistant cells overexpression of the E3 ligases Skp2 and UBE3C leads to ubiquitination of MST2 pathway proteins while CDKN2A loss or mutation disrupts MDM2 regulation causing p53 degradation [140, 186, 187]. The resulting impairment of ubiquitin signaling and loss of p53 activity heightens tumor aggressiveness and increases the refractory nature of resistant melanoma. Deubiquitination of cyclic GMP-AMP synthase (cGAS) by ubiquitin-specific peptidase 18 (USP18) alters the protein conformation, rendering it structurally stable. This subsequently increases cGAS expression in *BRAF V600E*-mutant melanomas, promoting resistance to BRAFi specifically within the *BRAF* mutant context [188]. Grigore et al. showed that chronic exposure of *BRAF V600E*-mutant melanoma cells to the selective BRAFi vemurafenib significantly increases H3K36me2/3. Because elevated H3K36 methylation rewires transcription toward pro-survival programs, these findings implicate H3K36 hypermethylation as an epigenetic driver of vemurafenib resistance [189]. Sirtuin 5, a member of the sirtuin deacylase family, maintains histone acetylation and methylation levels in melanoma cells and thereby sustains oncogenic transcriptional programs [190]. Inactivation of the neddylation signaling pathway by tetracaine hydrochloride effectively suppresses melanoma cell proliferation and alleviates vemurafenib resistance, highlighting a novel non-genetic mechanism underlying drug tolerance [191].

In summary, varying degrees of epigenetic alterations can confer resistance to BRAFi in *BRAF* mutant melanomas. Meanwhile, elucidating the diverse mechanisms underlying these epigenetic changes may provide a potential therapeutic strategy to enhance the sensitivity of resistant melanomas to BRAFi. Collectively documented hypermethylation of key gene promoters together with aberrant expression of miR19a, miR211 and the lncRNA SAMMSON, orchestrates transcriptional reprogramming in melanoma. Pharmacologic deployment of DNA methyltransferase inhibitors sequence-specific silencing of relevant non coding RNAs, and modulation of protein modifications may resensitize melanoma cells to BRAFi by reversing these epigenetic constraints.

### Metabolic reprogramming: adaptive metabolic liabilities in acquired resistance

Metabolic reprogramming serves as a critical adaptive strategy during acquired resistance, where melanoma cells remodel their metabolic routes, such as ramping up glutaminolysis and polyamine production, to survive BRAFi-induced growth suppression and oxidative damage. Bioinformatics analysis identifies EGFR as a ferroptosis-related driver of *BRAF V600E* melanoma resistance. Meanwhile, lactate promotes LSD1 lactylation, which suppresses ferroptosis via the LSD1-FosL1-TFRC axis. Conversely, MitoCur-1 blocks USP14 to inactivate GPX4, deplete GSH, and accumulate ferrous iron, thereby reactivating ferroptosis. Together these findings position ferroptosis modulation as a promising unified approach to resensitize resistant tumors and enhance immunotherapy efficacy [192–194]. Adding to this resistance landscape, reprogramming of the transsulfuration pathway, especially upregulation of cystathionine gamma lyase (CSE), drives acquired resistance to BRAF V600E inhibitors in melanoma. The enzyme boosts cystine and cysteine metabolism to produce persulfides and hydrogen sulfide, which shield cells from drug induced oxidative stress and meet increased energy demands. Preclinical studies show that combining BRAF V600E inhibitors with CSE inhibitors curbs proliferative relapse and extends progression free survival [43]. Polyamine biosynthesis and EIF5A hypusination downstream of c-Myc now explain a new vemurafenib resistance route in melanoma. CRISPR screens pointed to AMD1 as a druggable node and metabolomic plus proteomic data showed that resistant tumors boost polyamines which heightens EIF5A hypusination mitochondrial translation and oxidative phosphorylation. Sustained c-Myc keeps the polyamine flux high and blocking this pathway re-sensitizes tumors in vitro and in vivo spotlighting polyamine synthesis as a clinically actionable target to improve BRAFi efficacy [195].

Therapy-exposed BRAF mutant melanoma contains persister cells that spatial transcriptomics identifies as upregulating oxidative phosphorylation and invasion while downregulating proliferation. Their dependence on DUSPs, reticulon 4, and CDK2 for survival exposes vulnerable intervals where intervention could prevent resistance emergence [196]. In BRAFi-resistant melanoma cells, amplified glutaminolysis fuels survival, making glutaminase a metabolic vulnerability for overcoming resistance [33]. Metabolic reprogramming in resistant melanoma cells is not an isolated adaptive process, it is tightly intertwined with remodeling of the tumor microenvironment, where stromal cells and soluble factors further shape metabolic vulnerabilities and reinforce drug tolerance.

### The tumor microenvironment remodeling: stromal-immune synergistic resistance

**Fig. 4:**
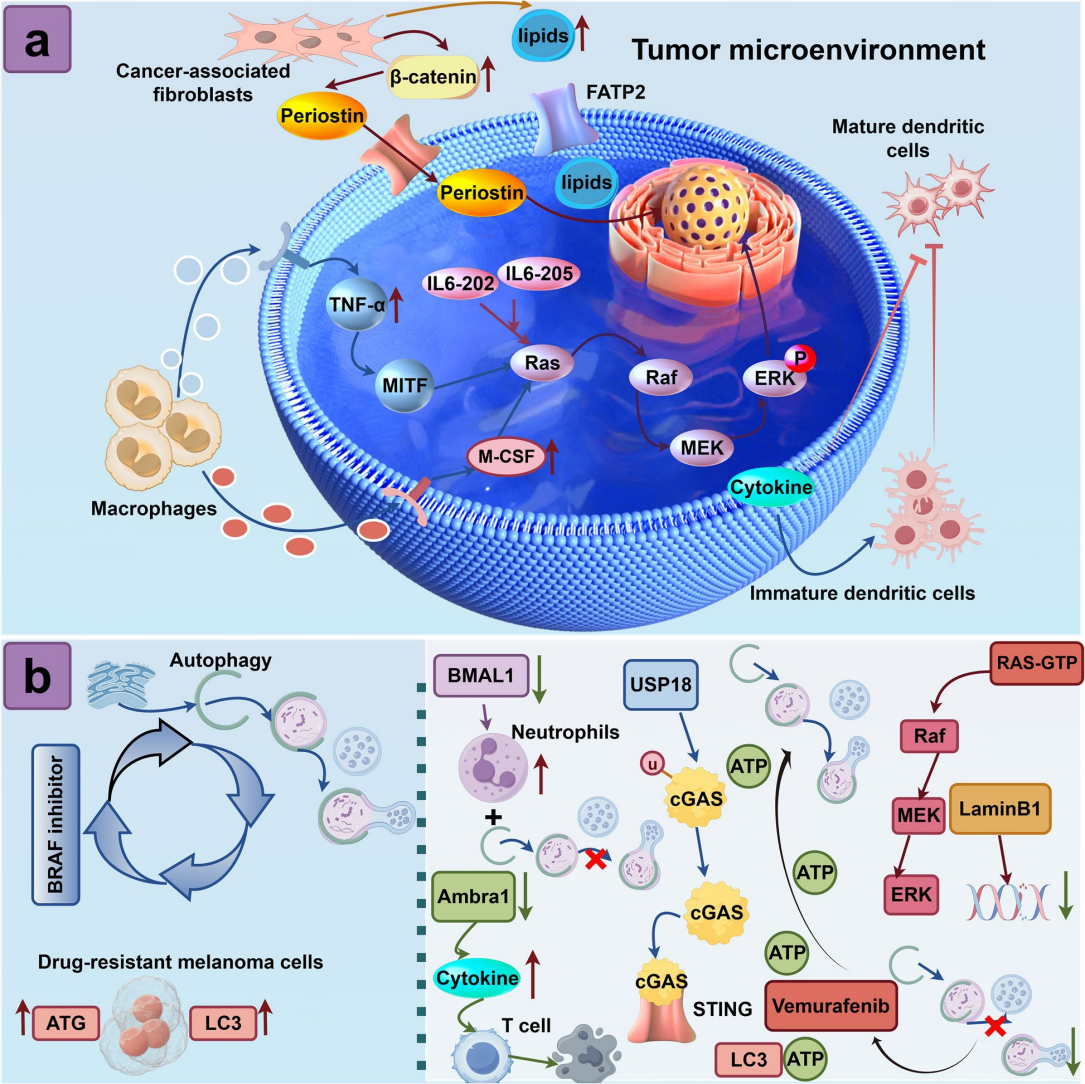
Tumor Microenvironment and Autophagy Networks in BRAF-Inhibitor Resistance: Myofibroblasts, Immune Cytokines, and Autophagy-Driven Feedback Circuits. **a** Tumor associated myofibroblasts and infiltrating immune cells including dendritic cells and macrophages orchestrate a cytokine milieu comprising TNF α, M CSF and IL 6 that confers BRAF inhibitor resistance in melanoma. **b** Autophagy-Driven Resistance Circuitry in *BRAF*-Mutant Melanoma: from Ambra1 Loss and BMAL1 Repression to USP18-cGAS Deubiquitination, LaminB1 Modulation, and ATP-LC3-ERK Feedback

TME comprising stromal cells, extracellular matrix, and soluble molecules, plays a pivotal role in melanoma progression and treatment resistance, highlighting its potential as a therapeutic target [34]. The progression of malignant tumors from early to advanced stages is significantly influenced by TME [197–199]. Mechanical and architectural signals within the tumor microenvironment significantly influence acral melanoma pathogenesis, inducing DNA damage and promoting a malignant phenotype, as revealed by a novel 3D in vitro platform [35]. Alterations in TME can promote resistance to BRAFi in *BRAF* mutant melanoma cells [200–203]. Fibroblasts in older men selectively drive melanoma cell invasiveness and therapeutic resistance to BRAFi, this phenomenon has been documented across both *BRAF* mutant and *BRAF* wild-type melanomas and therefore represents a pan-genotype microenvironmental driver [204]. Likewise aging fibroblasts in elderly melanoma patients secrete more lipids and co cultured young melanoma cells increase lipid uptake via fatty acid transporter FATP2 which reduces their sensitivity to targeted inhibitors [205]. BRAFi induce the activation of cancer-associated fibroblasts (CAFs), which in turn drive stromal remodeling and immune-evasion TME in melanoma [206–208]. Activation of β-catenin in CAFs stimulates the secretion of periostin, promoting resistance of specifically in *BRAF* mutant melanoma cells [209]. At the same time, Rho/MRTF pathway activation drives aggressive behavior in vemurafenib-resistant murine melanomas through enhanced stress fiber formation, MRTF-A nuclear entry and immune checkpoint upregulation provide a new target to overcome resistance and improve outcomes [210]. Drug-resistant cells display elevated IL-6 secretion, the IL-6–202 and IL-6–205 transcript variants confer BRAFi resistance in *BRAF* mutant melanoma [211]. Moreover, BRAFi resistant melanoma cells shed elevated soluble CD73, an ectonucleotidase that produces immunosuppressive adenosine and fuels resistance. Nutrient stress drives MMP-9 mediated CD73 release, which is blocked by CD73 inhibitors. While BRAFi treatment lowers CD73 in sensitive cells, resistant cells upregulate both membrane and soluble CD73, linking CD73 to acquired BRAF-targeted therapy resistance [212]. Vemurafenib-resistant melanoma cells not only modulate dendritic cell (DC) activation and cytokine production within TME, influencing DC maturation, but also exhibit elevated expression levels of IFN-γ, IL-8, VEGF, CD147/basigin and MMP-2 compared to their parental cell counterparts, these alterations have been observed in both *BRAF* mutant and *BRAF* wild-type melanomas, qualifying as pan-genotype mechanisms [213]. TNF-α derived from macrophages within TME can drive melanoma cells to develop resistance to MAPK inhibitors through the lineage-specific transcription factor MITF (microphthalmia-associated transcription factor), an event that occurs preferentially in *BRAF* mutant melanoma cells [36]. Elevated secretion of macrophage colony-stimulating factor (M-CSF) in drug-resistant melanoma cells and higher levels of M-CSF induce a drug-resistant phenotype in melanoma cells [214, 215]. This promotes BRAFi resistance in *BRAF V600E* mutant melanoma while increasing tumor migration and growth, and correlates with poor prognosis in patients with BRAF V600E mutant metastatic melanoma [214]. Cancer stem cells (CSCs) within the melanoma TME, regulated by the paracrine renin-angiotensin system, contribute to treatment resistance, highlighting the potential of targeting this system to enhance the efficacy of targeted therapy and immunotherapy [216]. Furthermore, BRAFi-resistant melanoma cells silence RICTOR/mTORC2 to boost mitochondrial respiration via the NAMPT-ETC axis and simultaneously overexpress and secrete NAMPT and NNMT free or in extracellular vesicles, sculpting a microenvironment that reinforces both intrinsic and extrinsic resistance to BRAF/MEK therapy [37, 217]. Therefore, the aforementioned findings indicate that alterations in TME can contribute to resistance in tumor cells to BRAFi. Some of these alterations are pan-genotype, whereas others are restricted to the *BRAF* mutant context. Investigation of TME-associated factors may represent a promising therapeutic target for overcoming BRAFi resistance. In addition to the paracrine signals and stromal remodeling mediated by CAFs and immune cells, autophagy emerges as a central hub that integrates TME cues with intracellular survival pathways, further amplifying BRAFi resistance in a bidirectional crosstalk between tumor cells and their microenvironment (Fig. 4a).

Autophagy functions as a central metabolic and signaling hub that dynamically sculpts the tumor microenvironment by regulating immune cell infiltration, cytokine release, and extracellular matrix remodeling [218–222] (Fig. 4b). This bidirectional interaction promotes immune evasion and therapy resistance while simultaneously providing actionable targets for combination cancer therapies [223–225]. As an example, loss of Ambra1 (autophagy and Beclin-1 regulator 1) upregulates multiple cytokines and chemokines, which in turn suppresses regulatory T cell infiltration and thereby promotes melanoma immune escape and drug resistance [220]. BMAL1 (aryl hydrocarbon receptor nuclear translocator-like) is markedly down-regulated in melanoma, impairing autophagy and increasing tumor-associated neutrophil infiltration to reshape the tumor microenvironment and drive malignant progression [226]. Autophagy plays a cytoprotective role in melanoma cells treated with BRAFi such as vemurafenib and dabrafenib, and its induction promotes tumor cell survival, contributing to drug resistance. Therefore, targeting autophagy represents a promising adjuvant strategy to enhance the efficacy of BRAF-targeted therapies and overcome resistance [227].

BRAFi-resistant melanoma cells exhibit increased basal levels of autophagosome markers and decreased autophagic flux [228, 229]. Conditioned media derived from these drug-resistant cells enhance the resistance of BRAFi-sensitive melanoma cells [230, 231]. Cellular autophagy has been associated with various mechanisms, such as the deubiquitination of cyclic GMP-AMP synthase (cGAS) by USP18. This process specifically triggers resistance-protective autophagy in *BRAF* mutant melanoma cells, conferring resistance to vemurafenib and other BRAFi [188]. Interfering with nuclear protein laminB1 enhances vemurafenib sensitivity in *BRAF*-mutated melanoma cells by promoting DNA damage and inhibiting protective autophagy, revealing laminB1 as a potential diagnostic marker and therapeutic target [232]. Vemurafenib-resistant cells exhibit heightened autophagic flux and ATP efflux, sustaining ERK autocrine signaling [233]. As drug resistance increases, autophagic flux increases and the co-localization of intracellular ATP with the pro-autophagic marker LC3 is enhanced [233].

Collectively, CAF-dependent β-catenin activation and macrophage-secreted M-CSF or IL-6 isoforms sustain proliferative and survival signaling within the resistant TME. Immune cell derived M -CSF IL 6 and TNF alpha drive stromal support and enhance *BRAF* mutant melanoma resistance to BRAFi. We conclude that BRAFi escape is not a single-pathway revival but a multi-layer rewiring of genetics, epigenetics and the tumor micro-environment. Looking forward, real-time integration of cfDNA methylation scores with single-cell enhancer maps should allow the first pre-emptive switch before imaging escape is even visible. Unsupervised analysis identifies key immune response genes and matrisome components as shared contributors to heterogeneous resistance mechanisms against BRAFi and MEKi in melanoma, highlighting their potential as therapeutic targets [234].

## Mechanism-driven strategies to overcome resistance: targeted, epigenetic, immune and novel therapies

Resistance arises from intertwined genetic, epigenetic, metabolic, and microenvironmental processes, requiring multi-pronged therapeutic designs. Such integrated approaches bridge the gap between laboratory findings and clinical efficacy.

### Targeted combination therapies: genomics-guided precision regimens

The goal of individualized precision therapy is to achieve the optimal treatment match for each patient, enhancing therapeutic efficacy and minimizing the risks and costs associated with ineffective treatments [235–239]. This treatment paradigm, which incorporates patient genomics and molecular profiling, is particularly crucial in cancer treatment, as tumors from different patients may exhibit distinct genetic and molecular profiles, necessitating personalized treatment strategies (Table 2). In clinical settings, individualized treatment plans can be developed based on patients' genomic characteristics and tumor molecular features [72, 240–243]. These plans may encompass palliative care, the selection of appropriate targeted agents and immunotherapies, and combination therapies [244–247]. They are all aimed at improving patients' quality of life while minimizing treatment-related adverse effects [248, 249]**.**

**Table 2:** Genomics-guided and targeted combination strategies to overcome tumor drug resistance via inhibition, degradation, and pathway modulation

Strategy Class	Key Combination/Agent	Mechanism Trampled	Ref
PARP add-on	• PARP inhibitor after BRAF/MEK failure	• Exploits increased replication stress • Deepens response	[39]
T-type calcium block	• Cav3.1 blocker + vemurafenib	• Inhibits autophagy-mediated survival • Restores apoptosis	[40]
PI3K rescue	• Neratinib (pan-ERBB/HERi) + BRAF/MEK in PTEN-null tumors	• Reverses PTEN-loss–driven PI3K/AKT hyper-activation & restores sensitivity	[41]
ABL1/2 block	• Nilotinib + BRAF ± MEKi	• Silences ABL-driven MAPK re-activation & prevents emergence of resistance	[101]
Genomics-guided matching	•Tailor combo to each patient’s mutation profile	• Cuts futile therapy & cost; boosts response	[235–239]
PROTAC degradation	•PROTAC that degrades *BRAF-V600E*	• Catalytic removal of mutant *BRAF* → deeper MAPK shut-off, slower resistance	[250]
Genome-wide scan	•Whole-genome sequencing of resistant tumor	• Finds actionable second-site mutations for next combo	[166, 251–256]
FANCD2 degradation	•Protocatechuic aldehyde + temozolomide	• Degrades FANCD2, boosts cytotoxicity regardless of MGMT status	[257]
Proteasome + ion channel	•Proteasomei + Kv1.3 blocker	• Synergistic apoptosis in sensitive & resistant cells	[258]

The strategies outlined, which encompass approaches from ABL1/2 inhibition to PROTAC-mediated protein degradation, are precisely tailored to counteract the genetic escape mechanisms. Such as MAPK pathway reactivation and PI3K/AKT bypass signaling, described earlier. This alignment underscores the meticulous design of therapeutic interventions to specifically target the underlying mechanisms of resistance.

A comprehensive genome-wide analysis of drug-resistant melanoma patients, leveraging advanced genomic techniques, holds significant potential to elucidate the underlying genetic mechanisms of resistance [166, 251–253]. This can provide crucial insights to inform the development of highly personalized treatment regimens and optimized clinical strategies tailored to the specific genetic profiles of individual patients. Utilizing whole-genome sequencing to analyze a patient's tumor sample enables the identification of specific gene mutations, copy number variations and gene expression profiles [254–256]. This comprehensive approach elucidates the mechanisms underlying drug resistance and informs the development of targeted therapies, enhancing the precision and efficacy of treatment strategies [39, 250].

Wongchenko MJ et al. meticulously analyzed and evaluated the gene expression profiles of *BRAF* mutant melanoma samples, including the expression of cell-cycle-related genes, in a bid to precisely target these profiles for the treatment of *BRAF* mutant melanoma [257]. In *BRAF* mutant melanoma that has acquired resistance to BRAFi, secondary gene mutations fuel the survival of resistant cells, and inhibitors targeting these mutations can halt their progression. For instance, nilotinib, an FDA-approved ABL1/2 tyrosine-kinase inhibitor, restores sensitivity to BRAFi ± MEKi in resistant melanoma. In vitro it abrogates cell viability and triggers apoptotic commitment. In vivo it elicits sustained regression of BRAFi/MEKi-refractory xenografts. And, when implemented as upfront therapy, effectively precludes the evolution of resistance [101]. PARP inhibitors produce partial or near-complete responses in advanced *BRAF V600-mutant* melanoma after BRAF/MEK progression, pointing to a synergistic PARPi plus BRAF/MEK regimen for refractory disease [40]. PROTAC-directed degradation of BRAF V600E demonstrates superior MAPK pathway inhibition and apoptotic induction compared to standard BRAFi in transcriptomic studies. This enhanced efficacy and diminished resistance risk highlight PROTACs as a compelling therapeutic avenue for resistant melanoma [258]. Protocatechuic aldehyde (PA) demonstrates a synergistic effect with temozolomide (TMZ) in enhancing cytotoxicity against both BRAFi-sensitive and -resistant melanoma cells by promoting FANCD2 degradation, offering a novel combinatorial chemotherapy strategy regardless of MGMT status [41]. The Cav3.1 gene isoform of the T-type calcium channel (TTCC) is highly expressed in vemurafenib-resistant *BRAF* mutant melanomas. TTCC blockers have been shown to induce apoptosis and impair tumor cell migration and invasion by inhibiting autophagy-mediated resistance in drug-resistant melanoma cells and in mouse xenograft models [259]. Thus, Cav3.1 may serve as a marker for the sensitivity of drug-resistant melanoma cells to combination therapy. TTCC blockers may provide a potential targeted therapy for vemurafenib-resistant *BRAF V600E*-mutant melanoma. A novel therapeutic approach combining proteasome inhibition (Proteasomei) with Kv1.3 potassium channel blockade demonstrates synergistic effects in inducing apoptosis in both drug-sensitive and BRAFi-resistant melanoma cells, offering a promising strategy to overcome therapy resistance and potentially extending to other *BRAF* mutant tumors [260]. In *BRAF V600E* melanoma, PTEN loss-of-function mutations occur in approximately 40 percent of tumors, leading to hyperactivated PI3K/AKT signalling that compromises the efficacy of combined BRAF/MEK blockade. Treatment with the pan-ERBB/HER inhibitor (pan-ERBB/HERi) neratinib restores drug sensitivity in these PTEN-null cells and effectively reverses the resistant phenotype [261].

In summary, integrative genomic profiling via whole-exome and long-read sequencing deciphers clonal architecture and bypass lesions driving BRAF/MEKi resistance in melanoma. Precision combination protocols that co-target ABL1/2, PARP, PROTAC-directed *BRAF V600E* degradation, FANCD2 proteolysis, TTCC or Kv1.3 blockade, and PI3K/AKT re-sensitization with pan-ERBBi achieve synchronized blockade of adaptive signaling, collateral survival circuits, and immune-evasive reprogramming, yielding augmented objective responses with attenuated off-target toxicity. While genomics-guided combination therapies effectively block known resistance pathways, novel small-molecule inhibitors expand the therapeutic arsenal by targeting previously undruggable nodes or non-oncogene addictions that emerge during BRAFi resistance.

### Novel small-molecule inhibitors: targeting resistance drivers beyond MAPK

Phosphoenolpyruvate carboxykinase 1 (PCK1) is upregulated in vemurafenib-resistant melanomas, activating PI3K/Akt and reducing ROS. Targeted inhibition of PCK1 with 3-mercaptopropionic acid restores vemurafenib sensitivity [262]. Equally important, RIDR-PI-103, a ROS-activated PI3K prodrug, blocks the PI3K/Akt pathway under high ROS and halts proliferation in BRAFi and MEKi -resistant melanoma, while diclofenac increases intracellular ROS and activates the p38/p53 pathway to augment cytotoxicity and apoptosis in BRAFi-resistant cells, offering new ways to beat resistance [263, 264]. Disulfiram (DSF), a copper ionophore, overcomes BRAFi resistance in melanoma by inducing mitochondrial dysfunction via copper-dependent oxidative stress and TXNIP upregulation, independent of MAPK inhibition, offering a novel therapeutic avenue [265]. Likewise, acyclic retinoid overcomes vemurafenib resistance in *BRAF V600E* melanoma by simultaneously blocking MAPK and PI3K/AKT/mTOR signaling, highlighting its promise as a new therapeutic agent [266]. VERU-11, a microtubule inhibitor, disrupts microtubule polymerization and induces microtubule depolymerization in drug-resistant melanoma cells. When combined with vemurafenib, it blocks the cell cycle, induces apoptosis and inhibits cell migration. This combination also increases p53 expression levels and suppresses the activation of the ERK/Akt signaling pathway [267]. The quinolinol small molecules MMRi62 and its derivative SC-62–1 induce p53-independent apoptosis in melanoma cells, including those resistant to BRAFi, by covalently binding to over 1500 cellular proteins and altering multiple metabolic and stress response pathways, pointing to their potential as multi-targeting agents to overcome BRAFi resistance [268]. C-terminal Hsp90 inhibitors (KU757, KU758) overcome MEK/BRAFi resistance in melanoma: they had similar IC50 in A375 and resistant A375 MEKi. KU757 downregulated resistance pathways (oxidative phosphorylation, AKT/PI3K/MTOR) and cell cycle, upregulated apoptosis, with hub genes (e.g., *NDUFA7, CDC20*) suggesting clinical benefit [269]. 3-bromopyruvate beats vemurafenib alone against resistant melanoma by blocking glycolysis and EMT, halting proliferation and invasion while killing resistant cells [270]. A novel antibody therapeutic, such as an Indolium1, has been shown to inhibit the proliferation, migration and invasion of *BRAF* mutant, vemurafenib-resistant melanoma cells. This effect is achieved by inhibiting the expression of the oncogenic factor pRB and inducing the expression of the tumor suppressor EPHA3, restoring the drug sensitivity of resistant cells [271].

ENOX2, the cell-surface disulfide-thiol exchanger that powers melanoma redox cycling, has emerged as a double-duty asset. High tumor levels track with shorter overall, disease-specific and metastasis-free survival, and the small-molecule inhibitor phenoxodiol shuts down ENOX2 activity, suppresses cell viability and can prevent acquired vemurafenib resistance [272]. Marine fungal extracts are providing new anticancer candidates, particularly mortiamide-D. This Mortierella-derived cyclic peptide achieves selective toxicity, eradicating both drug-sensitive and resistant melanoma cells at micromolar levels while remaining harmless to normal keratinocytes. Structure–activity work shows that swapping D-Ile for D-Arg at position 7 boosts membrane penetration and cytotoxic potency, underscoring mortiamides as optimizable scaffolds for future melanoma drugs that could be deployed alongside ENOX2 blockers to forestall resistance and improve patient outcomes [273]. Researchers have designed new benzimidazole derivatives containing oxindole and non-oxindole structures that simultaneously block BRAF V600E and ABL2 kinase activity. These dual inhibitors kill resistant melanoma cells, shut down key survival signals including p-CrkL and p-ERK1/2, halt cell cycle progression in G1 phase, and offer a viable strategy to tackle treatment-resistant disease [274].

Lipid metabolism-dependent processes and genes regulating lipid metabolism, such as SREBP-1, promote adipogenesis in *BRAF* mutant melanoma cells. This results in enhanced therapeutic resistance of tumor cells to targeted agents, conferring acquired resistance to these therapies [275–277]. However, targeting lipid metabolism can attenuate the resistance of *BRAF* mutant melanomas to BRAFi. Avasimibe, an SREBP inhibitor, can act synergistically with vemurafenib, increasing the occurrence of ferroptosis in tumor cells. This enhances the sensitivity of drug-resistant cells to the drug [278]. *BRAF*-mutated melanoma cells (A375, FO-1) developed distinct resistance signatures after long-term RAF inhibitor (dabrafenib/AZ628) exposure (dabrafenib-resistant: high proliferation, AZ628-resistant: slow-cycling, ferroptosis-susceptible). Antiretrovirals doravirine/cabotegravir reduced resistant cell viability, with doravirine specifically activating apoptosis and inhibiting growth in proliferative resistant cells via upregulating p16Ink4a/p27Kip1 [279]. In *BRAF*-mutated malignant melanoma, resistance to the second-generation BRAFi Encorafenib is mediated by NCOA4-regulated iron trafficking, leading to altered iron metabolism and ferritinophagy, as evidenced by increased levels of NCOA4, FTH1, and intracellular iron in resistant A375 cells [280]. Desmoplakin, a potential anticancer agent, can reverse the upregulation of the pentose phosphate pathway and lipogenesis in vemurafenib-resistant *BRAF* mutant melanoma cells. This drug not only upregulates the expression of the lactophilin family of protein-coding genes but also enhances T-cell activity, exerting an antitumor effect [281]. Dihydrotanshinone I (DHT), a lipophilic compound from *Salvia miltiorrhiza*, enhances BRAF/MEKi efficacy in *BRAF* mutant melanoma by directly inhibiting STAT3/SOX2 signaling, thereby overcoming primary and secondary resistance via dual MAPK-STAT3 pathway blockade [282]. The Rho/MRTF axis and its effector pirin drive melanoma drug resistance, and blocking Rho/MRTF with CCG-257081 restores vemurafenib sensitivity while preventing emergence of resistance by amplifying apoptosis, positioning pirin or Rho/MRTF inhibition as a promising tactic to overcome therapy failure [283]. Xanthohumol (XN), a natural compound derived from hops, enhances the sensitivity of melanoma cells to vemurafenib by reducing membrane cholesterol and increasing fluidity, thereby facilitating greater drug uptake and improving therapeutic efficacy with minimal toxicity to non-tumor cells [44]. A novel small molecule, EPS496, has been identified as a selective inhibitor of p300, a histone acetyltransferase implicated in transcriptional regulation and resistance mechanisms in melanoma. EPS496 demonstrated high binding stability and potent anti-proliferative activity in both normal and vemurafenib-resistant *BRAF V600E* mutated melanoma cells, offering a promising therapeutic strategy to overcome vemurafenib resistance [284]. In summary, studies targeting lipid metabolism may provide a viable strategy for the clinical management of vemurafenib-resistant *BRAF* mutant melanoma and for improving patient outcomes.

In summary, post-genomic metabolic liabilities PCK1, SREBP1, NCOA4 and ENOX2 propagate BRAFi/MEKi refractoriness; targeted small-molecule, ionophore and degradative interventions enforce ferroptotic oxidative stress and G1 arrest MAPK-independently. Single-cell PDO lipidomics will prioritize these non-oncogene addictions for adaptive, metabolism-driven phase I trials.

### Epigenetic and metabolic interventions: reversing adaptive reprogramming

Although epigenetic modifications are known to contribute to resistance to BRAFi in melanoma, the interplay between these alterations and associated signaling pathways remains a component of the resistance mechanism that is not yet fully elucidated. Thus, an in-depth investigation of epigenetics could provide a theoretical basis for the development of novel small-molecule inhibitors. Integrated multi-omics analysis of proteins and RNAs can elucidate the epigenetic regulation of proteins and RNAs in tumor cells [285].

It is worth noting that pharmacological modulation of epigenetic machinery and associated metabolism offers a promising therapeutic strategy for patients with resistant melanoma. In advanced disease upregulation of PDE4D methylation is observed and selective PDE4 inhibition effectively counters resistant melanoma cells [42]. Inhibition of EZH2, an epigenetic regulator linked to melanoma progression and BRAF therapy resistance, enhances vemurafenib activity in BRAFi-resistant cells by downregulating PLK1, leading to decreased viability, cell-cycle arrest, and increased apoptosis, indicating that targeting EZH2 or PLK1 together with BRAFi offers a potential new therapeutic strategy for *BRAF* mutant melanomas [286]. DNA methylation also drives the exhaustion of cytotoxic T cells during tumor progression and histone methyltransferase EZH2 cooperates with DNA methylation to promote resistance. Consequently, combined targeting of DNA methylation and EZH2 may be essential to overcome immunotherapy resistance in melanoma [45, 287]. LncRNAs play a critical regulatory role in melanoma biology [288, 289]. A combined CRISPR activation and small-molecule screen mapped lncRNAs that drive BRAFi resistance in melanoma, uncovering druggable lncRNAs and combination treatment options [290]. Suppression of lncRNA ROSALIND elevates reactive oxygen species and protein oxidation thereby inducing severe mitochondrial respiratory dysfunction and compromising melanoma cell viability [291]. Knockdown of lncRNA U73166 and LINC01291 markedly inhibits melanoma cell migration and invasion. Consequently lncRNA-directed therapies may serve as novel biomarkers and effective therapeutic targets for melanoma [46, 181]. Metabolic regulatory nodes such as autophagy play a pivotal role in melanoma [292, 293]. Pharmacological blockade of autophagy represents a potential strategy to circumvent therapeutic resistance [294]. The small molecule inhibitor SPP86 suppresses autophagy and exerts antineoplastic activity through attenuation of PI3K/AKT signaling [295]. Moreover, the antibiotic eravacycline induces autophagy while promoting M1 macrophage polarization thereby enhancing the efficacy of anti PD-1 therapy in melanoma [296]. Similarly, elevated expression of the histone lysine methyltransferase Suv39h1 promotes tumor immune escape, and inhibiting this gene enhances CD8 + T-cell infiltration [297].

Moreover, recent insights into oxidative stress in melanoma reveal its paradoxical role in tumor progression, where it initially promotes tumorigenesis but later hinders metastasis, and highlight redox metabolic reprogramming as a key factor in acquired resistance to BRAFi/MEKi, suggesting that modulating the antioxidant system could enhance therapeutic efficacy [47]. Mechanistically, the involvement of Nrf2, a key regulator of antioxidant responses, in the acquired resistance to BRAFi and MEKi in melanoma has been illuminated, where its upregulation and stabilization by DUB3 contribute to therapy resistance, and targeting Nrf2 or DUB3 presents a promising approach to reverse this resistance [298]. In patient-derived melanoma cells, the rewiring of oxidative phosphorylation, preservation of pyruvate dehydrogenase activity, and maintenance of high glutathione levels have been identified as crucial contributors to the onset of PLX4032 resistance, suggesting that targeting glutathione biosynthesis and/or pyruvate dehydrogenase activity in combination with PLX4032 may offer a viable strategy to overcome drug resistance in *BRAF*-mutated melanoma patients [299]. Cholesterol and its metabolite 27-hydroxycholesterol (27HC) have been implicated in promoting resistance to vemurafenib in melanoma by activating Rap1-PI3K/AKT signaling, suggesting that targeting 27HC synthesis could be a potential strategy to overcome treatment resistance [300]. In summary, combinatorial blockade of EZH2–DNMT–lncRNA axes, glutathione-dependent OXPHOS, 27HC-Rap1–PI3K signaling and autophagic flux reactivates cytotoxic T cells, exhausts antioxidant capacity and enforces ferroptosis, thereby ablating BRAFi/MEKi refractoriness. These convergent epigenetic, metabolic and redox interventions constitute a resistance-agnostic backbone for forthcoming adaptive basket trials in advanced melanoma.

### Immunotherapy combinations: the tumor microenvironment-targeted immuno-oncology strategies

TME shapes immune escape and drug resistance, thereby dictating therapeutic response in melanoma [301, 302]. Targeting this niche with targeted and immune therapies may achieve durable disease control [48, 303, 304]. The combination of the BRAFi and the MEKi has been widely employed in clinical practice for the treatment of *BRAF* mutant melanoma and has demonstrated significant efficacy in improving overall survival among patients (ClinicalTrials.gov identifier NCT01902173) [49, 305, 306]. However, as resistance develops, immune cells within the tumor also undergo corresponding changes, and novel therapies such as immunotherapy have therefore been introduced. For instance, vemurafenib resistance concurrently up-regulates MICA/ULBP2 and TRAIL-RII, markedly enhancing NK-cell degranulation and IFN-γ secretion. Serum levels of sMICA, sB7H6, PD-L1 and CEACAM1 correlate with clinical response, providing mechanistic and biomarker rationale for combining BRAFi/MEKi with NK-directed immunotherapy to overcome acquired resistance [307]. Moreover, RNA-seq and bioinformatics pinpoint a CD20 + MM subset that is intrinsically resistant to BRAFi, and pairing BRAFi with anti-CD20 antibodies markedly boosts killing of these cells, offering a promising way to prevent recurrence in melanoma patients carrying this CD20 + subpopulation [308]. The combination of atezolizumab, vemurafenib and cobimetinib increases the expression of lactate dehydrogenase (LDH) and programmed death ligand 1 (PD-L1) in TME and enhances the inhibitory effects of BRAFi on tumor cells. This combination significantly improves progression-free survival (PFS) in patients with advanced-stage *BRAF V600E*-mutant melanoma [309, 310]. Over the past two decades, melanoma treatment has moved from low-efficacy cytokine-based immunotherapy to PD-1 antibodies as the standard for advanced disease, alongside FDA-approved BRAFi and BRAF/MEK combinations for *BRAF* mutant tumors, changes that have produced the largest survival improvements seen in any cancer during the 2010s [311]. As an illustration, a LASSO model singled out high platelet-to-lymphocyte ratio, acral or mucosal subtype, *BRAF* mutation, low globulin, and multiple metastatic sites as key predictors of primary PD-1 inhibitor resistance in metastatic melanoma, giving clinicians early warning flags and hints for countermeasures [312]. In BRAFi/MEKi-refractory melanoma, EGFR overexpression was identified as a potential biomarker for responsiveness to second-line immune checkpoint inhibitors (ICIs), as demonstrated by increased CD8 + T effector cells and activation of the EGFR-STAT signaling pathway in resistant tumors, which retained sensitivity to ICIs, contrasting with NRAS-driven resistance showing cross-resistance to ICIs [313]. Moreover, the combination of atezolizumab, vemurafenib and cobimetinib can also serve as a first-line therapeutic option for patients with unresectable, advanced *BRAF V600E*-mutation-positive melanoma. This combination has demonstrated safety and tolerability in targeted therapy [6]. Subsequently, sequential administration of PD-1 checkpoint inhibitors (nivolumab or pembrolizumab) after first-line BRAFi/MEKi failure provides the greatest survival benefit to patients with advanced *BRAF* mutant melanoma [314].

The use of immunotherapy, either alone or in combination with targeted therapies, can help determine a patient’s eligibility for treatment with immune checkpoint inhibitors [315]. Liu S et al. loaded CpG or BMS-202 into liposomes and conjugated them to OT-1 CD8 + T cells (CD8-T-LP-CpG/CD8-T-LP-BMS-202), markedly reducing PMN-MDSCs, M2 macrophages, and Tregs while increasing the infiltration of cytotoxic and effector-memory CD8 + T cells within mouse tumors. This platform combines an immunomodulator with adoptive T cells, offering a readily translatable strategy to disrupt the immunosuppressive microenvironment of solid tumors and enhance adoptive T-cell therapy [316–318]. Likewise, inhibiting the Rho/MRTF pathway with CCG-257081 lowers PDL1 in BRAFi-resistant melanoma, boosts CD8 + T and B cell infiltration while reducing tumor-associated macrophages, and thereby restores anti-PD1 efficacy to overcome resistance and enhance immunotherapy response [319]. Nivolumab, an anti-PD-1 antibody, synergizes with CD26-high CD4⁺ T cells to produce clinical benefit in patients with PD-1-resistant melanoma, and their overall survival is longer than 12 months [50]. Yet BRAF blockade heightens tumor immunogenicity by upregulating immune activation genes, drawing activated cDC1 and cDC2 into the tumor and draining nodes, and this myeloid remodeling is essential for the CD4 + and CD8 + T cell response that drives therapeutic efficacy [320]. Combination therapy involving BRAFi, MEKi and PD-1 checkpoint inhibitors in patients with *BRAF V600E*-mutant melanoma not only enhances antitumor efficacy but also improves outcomes for a subset of metastatic patients by increasing the frequency of durable responses [321]. In melanoma resistant to vemurafenib, the cell-permeable STAT3 inhibitory peptide APTSTAT3 9R increases cytotoxic T lymphocyte (CTL) infiltration into TME. Combined with anti-PD-1 antibodies, this therapy reduces myeloid suppressor cell and tumor-associated macrophage infiltration, while enhancing CD8 + T cell infiltration and cytotoxicity, significantly inhibiting tumor growth [322]. Likewise, within TME, Tim-3 and STAT3 are upregulated in anti-PD-1-resistant melanoma, which limits Treg-mediated immunosuppression, and targeting STAT3 significantly enhances PD-1 immunotherapy in mouse melanoma models [323]. Furthermore, recent research has identified Diosmetin (DIOS), a naturally occurring flavonoid compound, which significantly augments the antitumor activity of BRAFi in melanoma by concurrently suppressing the MAPK and JAK2/STAT3 signaling pathways. DIOS not only exhibits potent antiproliferative effects but also downregulates PD-L1 expression in melanoma cells, thereby enhancing intratumoral T cell infiltration and activating antitumor immune responses, providing a novel adjuvant strategy for *BRAF* mutant melanoma therapy [324].

The contribution of integrin and TGF-β signaling to vemurafenib resistance in metastatic melanoma is well established. Strategic co-targeting of ITGA5, ITGB3, PAI1, or p21 alongside vemurafenib generates synergistic suppression of proliferation, invasion, and clonogenicity in resistant populations, presenting a rational approach to circumvent resistance and boost clinical benefit [51]. The triple combination of vemurafenib, a Toll-like receptor 7 (TLR7) agonist, and a PD-1 antibody synergistically enhances the induction of melanoma antigen gp100-specific T cells in a *BRAF* mutant melanoma mouse model [325]. This augments T-cell immunoreactivity and promotes the efficacy of tumor-specific T cells, tumor-specific T cells can be used to treat patients with metastatic melanoma and have demonstrated feasibility and safety(NCT02424916) [325, 326]. Romidepsin plus IFN-α2b reverses vemurafenib resistance in primary melanoma cells by blocking tumorigenic signals, restoring immune responses silenced via histone deacetylation, and boosting immunogenicity, offering a promising approach for BRAFi-refractory metastatic disease [52]. In TME, HDAC inhibitors transcriptionally up-regulate PD-1/PD-L1 on both tumor and immune cells, sensitizing *BRAF* mutant-bearing mice to PD-1/PD-L1 blockade [327]. Concurrent HDAC and MAPK/MEKi synergistically enhances PD-1 blockade efficacy, increases intratumoral CD8 + T-cell infiltration, and yields significant antitumor activity [328, 329]. Thus, research has increasingly focused on combination therapies as potential effective treatment strategies for overcoming resistance to BRAFi in *BRAF* mutant melanoma within clinical settings (Table 3).

**Table 3:** Immuno-oncology tactics to overcome resistance: PD-1/PD-L1 boost, NK activation, multi-pathway blockade, metabolic reprogramming, biomarkers, and target degradation

Intervention Focus	Tested Agent/Regimen	Immune Pathway Modulated	Ref
CD26-high CD4 help	Nivolumab + CD26-high CD4⁺ T cells	• Prolongs OS > 12 months in PD-1-refractory patients	[50]
Integrin/TGF-β axis block	ITGA5/ITGB3/PAI1/p21 inhibitors + vemurafenib	• ↓Proliferation • ↓Invasion • Colony formation of resistant cells	[51]
HDAC + IFN boost	Romidepsin + IFN-α2b with vemurafenib	• Re-awakens silenced immune genes • Immunogenicity	[52]
NK-cell ligand up-regulation	Add NK-directed Rx to vemurafenib	• ICA/ULBP2 & TRAIL-RII up-regulated → ↑ NK degranulation & IFN-γ • Serum sMICA/sB7H6/PD-L1/CEACAM1 correlate with response	[307]
CD20⁺ resistant subset elimination	Anti-CD20 mAb + BRAFi	• Edicates CD20⁺ resistant sub-pop • Pevents recurrence	[308]
PD-L1-boost triplet (IMspire150 regimen)	Atezolizumab + vemurafenib + cobimetinib	• ↑LDH & PD-L1 in TME • Improves PFS vs targeted alone	[309, 310]
LASSO predictive signature	High PTL, acral/mucosal, *BRAF* mutation, low globulin, multiple mets	• Early warning of primary PD-1 resistance • Guides counter-measures	[312]
EGFR biomarker for ICI response	EGFR over-expression in BRAF/MEK-refractory tumors	• Identifies ICI-sensitive subset • EGFR-STAT activation linked to retained ICI benefit vs NRAS-driven cross-resistance	[313]
Sequential PD-1 blockade	Nivolumab/pembrolizumab after BRAF/MEK	•Greatest OS benefit when PD-1 given second-line	[314]
metabolic T-cell help (liposome-conjugated)	CpG or BMS-202-liposome conjugated to OT-1 CD8⁺ T cells	• ↓PMN-MDSC/M2/Treg • ↑Cytotoxic & memory CD8⁺ T cells	[316–318]
Rho/MRTF pathway inhibition	CCG-257081 + anti-PD-1	• ↓PDL1 • ↑CD8⁺/B cells • ↓TAM • Restores anti-PD-1 efficacy	[319]
cDC1/2 recruitment (BRAFi alone effect)	BRAFi alone	• ↑Immune-activation gene signature • Recruits cDC1/2 • Fuels CD4⁺/CD8⁺ T-cell response	[320]
STAT3 peptide inhibition	APTSTAT3 9R peptide + anti-PD-1	• ↑CTL infiltration •↓ MDSC & TAM reverses vemurafenib resistance	[322]
Tim-3/STAT3 dual hit	STAT3 inhibition + anti-PD-1	• Reverses Tim-3–mediated Treg suppression • Boosts PD-1 efficacy	[323]
Diosmetin adjuvant	Diosmetin flavonoid + BRAFi	• Dual MAPK & JAK2/STAT3 suppression • ↓PD-L1 • ↑T-cell influx	[324]
TLR7 agonist triplet	Vemurafenib + TLR7 agonist + PD-1 Ab	• ↑Gp100-specific T cells • Augments T-cell immunoreactivity	[325]

### Emerging therapeutic approaches: CRISPR, nanomedicine, and herbal-derived agents

The development of novel targeted therapies can enhance the efficacy of BRAFi and reduce adverse effects. Their combination with BRAFi can overcome resistance in *BRAF* mutant melanoma cells. This approach also provides a viable strategy for improving patient outcomes, representing a significant direction in contemporary cancer therapy. CRISPR/Cas9 enables precise in vivo gene editing and is emerging as a central platform for cancer therapy. Using targeted CRISPR-Cas systems in relevant HER2-positive ovarian cancer models results in powerful permanent gene correction, induces considerable tumor regression, and expands the landscape of druggable targets to enable durable anti-cancer efficacy [39]. Goh CJH et al. performed a genome-wide CRISPR/Cas9 screen to identify genes that regulate resistance to Vemurafenib in melanomas harboring the *BRAF V600E* mutation. Their study pinpointed several key genes, including NF1/2, CUL3, MED10/12, FOXD3, ANAPC11. Alterations in these genes have been shown to confer resistance to Vemurafenib in drug-resistant melanoma cases [330]. Genome-scale CRISPR-Cas9 transcriptional activation screening identified three BRAFi resistance-associated lncRNA genes (SNHG16, NDUFV2-AS1, and LINC01502) in melanoma, revealing a lncRNA-miRNA-mRNA regulatory network that provides new insights into the mechanisms underlying BRAFi resistance [331]. Equally significant, CD133-positive melanoma stem cells have been shown to acquire resistance to trametinib. CRISPR–Cas9-mediated, precise knockdown of CD133 effectively triggers melanoma-cell apoptosis, providing compelling evidence that CD133 is a high-value target for combination therapeutic strategies [332]. The evolution of *BRAF*-targeted therapies in melanoma underscores the necessity for innovative strategies to overcome therapeutic resistance, highlighting emerging targets such as ERK5 and CD73, and the potential of advanced tools like mRNA vaccines and CRISPR-Cas9 in personalized oncology [333]. In *BRAF*-mutated melanoma, increased expression of the cancer stem cell marker CD271, driven by Nox4-derived reactive oxygen species (ROS), mediates resistance to BRAFi like vemurafenib. Inhibition of Nox with DPI reduces CD271 expression, ERK and Akt signaling, and epithelial-mesenchymal transition (EMT), thereby suppressing drug resistance and metastatic potential [334].

The development of drug resistance in melanoma is closely associated with mutations in the *BRAF* gene, making it a prime target for gene-editing therapies utilizing CRISPR/Cas9 technology. However, the delivery of CRISPR/Cas9 remains a significant challenge. Nanoparticles based on nanotechnology, such as multifunctional lipid nanoparticles, have been shown to effectively deliver CRISPR/Cas9-sgRNA ribonucleoprotein complexes to target the *BRAF* gene in murine models. This delivery system efficiently edits melanoma cells and reduces *BRAF* expression. It inhibits melanoma progression [335]. Red-blood-cell-membrane nanocages that co-deliver CRISPR–Cas9 and ACC inhibitors achieve potent, dual metabolic–genetic suppression of tumor in vivo [336]. Complementing this, intratumoral PLNPs containing Cas9/sgPLK-1 plasmid reduce A375 tumor burden by > 67%, highlighting the platform’s strong translational promise [337]. Two distinct lipid-based delivery systems show promise against BRAFi-resistant melanoma. An oral nanocomplex combining BRD4 PROTAC ARV-825 with vemurafenib, and a gene therapy approach co-delivering PTEN plasmid with BRD4 PROTAC, both suppress c-Myc, demonstrate synergistic cytotoxicity in vitro, and achieve significant tumor suppression and apoptosis in vivo [338, 339]. Consequently, the development of targeted therapies aimed at these specific genes holds promise as a viable strategy for the treatment of melanoma patients. Nanotechnology-based delivery systems hold significant potential for effectively targeting tumors. Furthermore, the tumor suppressor protein p53 is often inactivated in *BRAF* mutant melanomas. However, its activator SLMP53-2 targets p53 to restore transcriptional activity, curbing melanoma aggressiveness and synergizing with vemurafenib to resensitize resistant tumor cells and exert anti-tumor effects [340]. The p53 family members TP53, TP63 and TP73 participate in acquired MAPK inhibitor resistance in melanoma, with specific isoforms showing elevated expression that directly fuels tumor cell proliferation, survival and therapy evasion, pointing to these proteins as potential therapeutic targets to overcome resistance [341]. Furthermore, encorafenib, a novel BRAFi, has been approved for the treatment of melanoma and colorectal cancer [342–344]. Encorafenib was found to inhibit drug efflux mediated by the ABC transporter protein ABCB1, reducing drug resistance in *BRAF* mutant melanoma cells and exerting antitumor effects [345]. Thus, encorafenib may serve as a therapeutic agent for tumors characterized by *BRAF* mutations and overexpression of the ABCC1 transporter protein.

Ezrin, a cytoskeletal protein involved in cell junctions in tumor cells, is significantly associated with the development of resistance to vemurafenib in *BRAF V600E*-mutant melanoma. Targeting this protein not only enhances the sensitivity of drug-resistant cells to vemurafenib but also synergistically enhances the antitumor effects of vemurafenib when combined with its inhibitor in drug-resistant melanoma cells [346]. The expression of cysteine sulfinyl reductase Sestrin2 is elevated in vemurafenib-resistant *BRAF* mutant melanomas. Knockdown of Sestrin2 enhances the sensitivity of these drug-resistant cells to vemurafenib. Guo et al. demonstrated that combining an mTOR inhibitor with Sestrin2 knockdown and vemurafenib synergistically enhances the antiproliferative and pro-apoptotic effects of vemurafenib in drug-resistant melanoma [347]. Furthermore, combining statins, which are inhibitors of 3-hydroxy-3-methyl-glutaryl-coenzyme A reductase (HMGCR), with disopyramide, an inhibitor of sterol regulatory element-binding protein 2 (SREBP2), may enhance the efficacy of vemurafenib in patients with *BRAF V600E*-mutant, drug-resistant melanoma. This combination may represent a significant advancement in the treatment of resistant melanoma [348]. Mechanistically, Kisspeptin-54, a KiSS1-derived peptide, enhances vemurafenib-induced apoptosis in BRAF mutant melanoma cells according to their PLX4032 sensitivity while modulating apoptotic regulators, revealing a new route to defeat BRAFi resistance [349]. Connexin43 (Cx43) has been identified to enhance the response to BRAFi/MEKi by reducing DNA repair capacity, promoting persistent DNA damage, and inducing cellular senescence, thereby offering a novel therapeutic strategy to overcome drug resistance in advanced *BRAF* mutant tumors [350]. PF-07799933, a brain-penetrant pan-mutant BRAFi, shows preclinical and clinical activity against *V600* and non-*V600* mutants and beats current RAF resistance through dose escalation guided by pharmacokinetics, offering a promising new option [351]. Thus, the development of novel targeted agents and their integration with BRAFi holds promise for the treatment of *BRAF* mutant, drug-resistant melanoma.

Herbal-derived small molecules and standardized botanical extracts have emerged as a complementary strategy to overcome BRAFi resistance in melanoma [352–354]. Emerging studies show that violacein, a natural pigment derived from Chromobacterium violaceum, significantly enhances the efficacy of vemurafenib in *BRAF*-mutated melanoma spheroids by downregulating key mediators such as fatty acid synthase (FASN), thereby inducing apoptosis and improving treatment response [355]. In pre-clinical models, cumingianoside A, a triterpenoid saponin isolated from the leaves and fine twigs of cumingia latifolia, has been shown to block the cell cycle and and supress autophagy-mediated resistance in vemurafenib-resistant *BRAF* mutant melanoma cells [356]. Cumingianoside A demonstrated significant inhibitory effects on tumor growth in xenograft mouse models, whether used alone or in combination with vemurafenib [356]. Vitexin compound 1 from Zingiber officinale inhibits BRAFi-resistant melanoma cells through ROS elevation and consequent DNA damage. This monomer does not adversely affect normal cells [357]. Furthermore, curcumin, as a single therapeutic agent, can enhance ROS production and disrupt mitochondrial membrane potential. It can induce apoptosis in drug-resistant *BRAF* mutant melanoma cells. Combining curcumin with gefitinib enhances the ability to inhibit the proliferation of drug-resistant cells [358]. Ailanthone, a novel c-Jun inhibitor, enhances the efficacy of anti-PD-1 therapy in melanoma and attenuates the immunosuppressive function of Tregs within the tumor microenvironment [359]. Cyclic membrane-active peptides, such as cyclic tachyplesin I (cTI), demonstrate potent efficacy in killing proliferative, non-proliferative, and drug-resistant melanoma cells, including those with the *BRAF V600E* mutation, without inducing resistance, offering a promising alternative therapeutic strategy to combat acquired drug resistance in melanoma [360]. Although these compounds exhibit low toxicity in vitro and in vivo, their clinical value remains to be established by well-designed randomised controlled trials. At present, no herbal products have received FDA approval for melanoma, and some plant-derived compounds such as aristolochic acid are recognized carcinogens. Therefore, rigorous pharmacological standardisation and Phase I/II safety studies are essential before translating these agents into clinical practice. Based on the resistance patterns outlined in this review, monotherapy or combined targeted agents only delay the onset of resistant disease and do not achieve lasting control. These findings indicate that ERK and PI3K inhibitors plus immunotherapy should be moved to the first line. Initiating randomized triple or quadruple regimens at cycle one represents the logical next phase, shifting from reactive rescue to a proactive first strike with emerging targeted agents and technologies.

## Emerging technologies shaping resistance management and future directions

To maximize the efficacy of the mechanism-driven therapies discussed in the aforementioned section, emerging technologies offer transformative tools for real-time resistance monitoring, precise patient stratification, and optimization of treatment regimens for closing the gap between preclinical discoveries and clinical application.

### Liquid biopsies: non-invasive monitoring of resistance dynamics

cfDNA detection offers high sensitivity and specificity for clinical tunor samples harboring mutant DNA at frequencies as low as 1 to 5% [55, 361–365]. Silva S et al. employed whole-genome sequencing to create a cfDNA copy number profile for 83 melanoma patients. Their findings revealed that cfDNA is not only relatively stable in plasma but also that an increase in its copy number is strongly correlated with *BRAF V600E*-mutant melanoma [366]. The *BRAF V600E* mutation was detected in 14 (18%) of the 76 successfully amplified cfDNA samples. CfDNA concentrations were found to be significantly elevated in patients with active melanoma compared to healthy controls [366]. This finding indicates that gene copy number analysis may function as a potential prognostic indicator of survival and a biomarker for patients with active melanoma. CfDNA profiling enables comprehensive surveillance of emergent targetable and resistance-associated mutations throughout treatment [53]. This technology therefore supports precision personalization of therapy for patients with drug-refractory melanoma.

Noninvasive metabolic imaging using 1H and 1H/31P MRS captures immediate biochemical responses to dabrafenib in melanoma models. The technology tracks sensitivity through lactate and alanine accumulation and measures bioenergetic capacity via βNTP/Pi ratios, offering a promising approach to forecast patient outcomes with signaling pathway inhibitors [367]. Vemurafenib triggers a noncanonical senescence associated secretory phenotype in *BRAF V600E* melanoma cells, releasing CCL2, TIMP2, and NGFR that shield neighboring tumor cells from growth inhibition, so co-targeting senescent cells and these cytokines may defeat vemurafenib resistance [368]. Ranolazine, an FDA and EMA approved anti-anginal drug, rewires melanoma metabolism by blocking fatty acid oxidation and boosting the methionine salvage pathway, delaying acquired BRAFi resistance and enhancing immunogenicity to improve both targeted therapy and immunotherapy responses [56]. The new patient-derived cutaneous melanoma line MelT79 carries both *BRAF V600E* and the rare RET S649L mutation, giving researchers a unique tool to dissect how combined alterations fuel heterogeneity, progression, and BRAFi resistance [369].

### Single-cell omics: deciphering tumor heterogeneity and resistance clones

Single-cell RNA-seq (scRNA-seq) has become the cornerstone for dissecting melanoma heterogeneity, revealing cell-state switches that underlie BRAFi resistance [370–373]. A study created and fully characterized the vemurafenib-resistant lines A375V, SK-MEL-28V, and RPMI-7951, which show altered morphology, faster growth, and greater invasiveness, providing a robust model to define resistance mechanisms and test new drugs against BRAFi resistance in melanoma [374]. Leveraging these transcriptomic maps guides the identification of targetable RNA networks and accelerates precision strategies [375, 376]. For instance, the dynamics of gene regulation can be understood through the study of protein chromatin open transitions in epigenetics using single-cell Assay for Transposase-Accessible Chromatin with sequencing scATAC-Seq technology [57]. According to scATAC-seq profiles, melanoma drug resistance is tightly linked to TME [377]. scRNA-seq analysis of tumor-specific genes reveals immune infiltration and tumor micro-environment remodeling in melanoma, improving patient prognosis prediction and the feasibility of gene-targeted therapy [378–381]. Tumor-infiltrating T cells exhibit an exhausted phenotype while cancer-associated fibroblasts accumulate, together orchestrating immune escape and resistance to immunotherapy [382–385].

### AI-driven drug discovery: predicting resistance and optimizing combinations

Artificial intelligence (AI) is reshaping anti-cancer drug development by forecasting resistance before it emerges and ranking effective drug pairs within minutes [58, 386, 387]. Machine learning models that mine large-scale omics and literature data rapidly identify next-generation inhibitors and optimal combination strategies for *BRAF* mutant tumors, offering a powerful platform for precision oncology [388]. For instance, machine learning models can detect specific downregulation events linked to CD8 + T-cell infiltration during tumor drug resistance, offering actionable insights for immune checkpoint inhibitor development [389]. Machine learning-based prognostic signatures independently and robustly predict melanoma patient outcome, and algorithm-guided analyses further illustrate the benefit of combining kinase inhibitors with mTOR inhibitors, DNA-damaging agents or HDAC inhibitors [390]. Although artificial intelligence facilitates daily life, models trained on biased or small datasets frequently yield overfitted predictions that fail across diverse patient cohorts. Their black-box architecture hampers biological interpretation and regulatory validation, thereby restricting clinical translation [391, 392]. AI-driven identification of optimal drug combinations and resistance predictors has accelerated the development of adaptive clinical trial designs, which prioritize real-world data to rapidly translate preclinical insights into patient-centric treatments. Accumulating real-world clinical evidence is therefore essential before AI-based disease and therapeutic predictions can be reliably implemented.

### Clinical trial innovations: adaptive designs for precision salvage therapy

New clinical trial designs use real-time data to quickly assign patients to the best BRAFi treatments as resistance develops. As an illustration, baseline and early [18F] FDG PET/CT-derived metabolic tumor volume and total lesion glycolysis predict progression-free survival in advanced *BRAF V600*-mutated melanoma on BRAFi/MEKi better than traditional SUV metrics [393]. How quickly metastases grow before treatment starts serves as a standalone predictor of survival in advanced *BRAF V600* melanoma on targeted agents. Rapid baseline growth worsens prognosis regardless of other factors like LDH, where tumors have spread, overall disease load, or prior therapy lines [394]. Furthermore, a phase 1 trial exploring the combination of vemurafenib, cobimetinib, and the HSP90 inhibitor XL888 in advanced *BRAF V600*-mutant melanoma demonstrated significant antitumor activity, albeit with notable toxicities necessitating dose reductions. Combining HSP90 blockade with BRAFi and MEKi yielded responses in over three quarters of patients, but durability proved challenging with 7.6 months median progression-free survival and 37% five-year survival, underscoring the mixed results of this intensive regimen [59]. Re-challenging with BRAF/MEKi after a drug-free interval can still elicit objective responses in patients who previously acquired resistance, as retrospective data show measurable response rates and progression-free survival during third or fourth courses, supporting repeated cycles alongside other agents [395]. Data from SECOMBIT trial demonstrate that the combination of targeted therapies, including BRAFi and MEKi, with dual immunotherapy targeting PD-1 and cytotoxic T-lymphocyte-associated protein 4 (CTLA-4), significantly improves overall survival (OS) and progression-free survival (PFS) in patients with metastatic melanoma harboring the *BRAF V600E* mutation (NCT05732805) [81, 396]. Crucially, the combination of anti-PD-1 therapy and BRAFi has demonstrated acceptable safety in patients with advanced melanoma, and longitudinal clinical benefit can be predicted using validated biomarkers (NCT02967692) [16, 397]. Through clinical trials, including BRAFi in metastatic melanoma phase II trial (BRIM-2), BRAFi in metastatic melanoma phase III trial (BRIM-3), BRAFi in metastatic melanoma phase Ib/II combination trial (BRIM-7), cobimetinib plus vemurafenib combination phase III trial (coBRIM), researchers have demonstrated that the combination of cobimetinib and vemurafenib significantly mitigates the adverse effects associated with vemurafenib monotherapy, particularly in patients who previously exhibited poor outcomes with vemurafenib alone [257, 398, 399]. This finding not only elucidated that combination therapy more effectively suppresses the expression of cell-cycle-related genes compared to monotherapy, but also led to a significant improvement in progression-free survival (PFS) among patients. These innovative clinical trial designs not only validate the mechanism-driven therapies outlined earlier but also address unresolved questions such as optimal treatment sequencing and toxicity management, paving the way for personalized salvage therapy in BRAFi-resistant melanoma. However, the NeoTrio trial shows that sequential therapy (targeted therapy followed by neoadjuvant immunotherapy) yields higher disease-free survival than concurrent therapy, while the concurrent arm has more recurrences and the highest toxicity (NCT02858921) [400]. Thus, new clinical evidence is needed to confirm the safety of combining targeted therapy with immunotherapy.

Overall, this section emphasizes four pioneering technologies: liquid biopsy, single-cell omics, AI-driven drug discovery, and adaptive trial design. These technologies collectively have the capability to anticipate and counteract resistance to BRAFi. The crucial message is that these tools should not operate independently. Instead, an integrated framework that incorporates cfDNA monitoring, single-cell profiling, AI-guided decision-making, and adaptive randomization is vital. This integration will enable a shift from reactive rescue strategies to a genuinely proactive and precision-focused therapeutic approach.

## Conclusion

Mutations in the *BRAF* gene, as well as other genetic and epigenetic alterations, predispose patients to recurrence and metastasis following surgery [401, 402]. For instance, patients with sigmoid colon cancer harboring both a *BRAF* mutation and an NTRK3 fusion gene were found to develop metastases following surgical resection [403]. The introduction of BRAFi has revolutionized the treatment landscape for *BRAF* mutant melanoma. However, as resistance to vemurafenib, dabrafenib in combination with trametinib and other therapies continues to emerge, the development of novel targeted agents has become a critical need, facilitating improved clinical care for patients with drug-resistant disease. Through clinical observation, periodic assessment of tumor response and the development of drug resistance allows for timely adjustment of treatment plans [404]. While BRAFi have demonstrated substantial clinical efficacy, the development of drug resistance presents a formidable challenge. Consequently, addressing the issue of tumor resistance to BRAFi is crucial for enhancing the clinical efficacy of these agents. The integration of whole-genome analysis, liquid biopsies (e.g., cell-free DNA testing), individualized therapy, new drug development and combination therapy strategies can enhance therapeutic efficacy, overcome tumor resistance and improve patient prognosis.

This article provides a detailed explanation of the theoretical underpinnings of the mechanism of action of BRAFi and explores the mechanisms of drug resistance in *BRAF* mutant melanoma. It outlines strategies for overcoming and reversing this resistance. It aims to promote the rational use of these agents by clinicians and mitigate tumor resistance, improve patient outcomes, and provide new research directions for investigators studying *BRAF* mutant melanoma. Despite the rapid accumulation of pre-clinical and clinical data, several unresolved issues temper the conclusions presented herein:(i) The causal hierarchy linking MAPK re-activation, PI3K/AKT bypass signalling and microenvironmental reprogramming remains incompletely understood, recent single-cell studies suggest that these events may occur in parallel rather than in a linear sequence, complicating the design of combination strategies [57, 325]. (ii) The prognostic value of circulating *BRAF V600E* cfDNA in early-stage or non-metastatic melanoma remains controversial, with discordant results across independent cohorts [115, 366]. (iii) Phase III clinical evidence is still lacking for several promising triple regimens, such as combining a BRAFi, TLR7 agonist, and PD-1 blockade, and the immune-related toxicity risks may outweigh their benefits in unselected patient populations [325]. (iv) Optimal sequencing of targeted therapy followed by immunotherapy (or vice versa) has yet to be defined, and validated predictive biomarkers for this decision are absent [81]. (v) The clinical translation of CRISPR/Cas9-based gene editing or nanoparticle delivery systems is hindered by tissue-specific targeting, off-target mutagenesis and scalable manufacturing challenges [335]. Addressing these limitations will be essential for converting mechanistic insights into durable clinical gains. This review provides new research avenues for investigators focusing on *BRAF* mutant melanoma to elucidate potential resistance mechanisms, identify corresponding tumor markers and develop drugs to counteract BRAFi resistance. These efforts can enhance or synergize the effects of BRAF-targeted therapies, improving therapeutic efficacy in vemurafenib-resistant melanoma and enhancing patients' quality of life.

## Data Availability

The data referenced in this study are primarily sourced from publicly accessible databases: PubMed andweb of science. Specific literature and datasets can be accessed as follows: PubMed: A doi has been provided after each reference. As this study did not generate new datasets or raw data, there are no additional data to share. All necessary data and analysis results are described in detail within the article and can be accessed through the iaforementioned links.

## References

[1] Darabi S, Stafford P, Braxton DR, Zuazo CE, Brodie TJ, Demeure MJ. BRAF V600E mutation has variable tumor-specific effects on expression of MAPK pathway genes that could affect patient outcome. Int J Mol Sci. 2025. 10.3390/ijms26167910. 40869231 10.3390/ijms26167910 PMC12386775

[2] Chen X, Chen X, Xie W, Ge H, He H, Zhang A, et al. BRAF-activated ARSI suppressed EREG-mediated ferroptosis to promote BRAF V600E (mutant) papillary thyroid carcinoma progression and sorafenib resistance. Int J Biol Sci. 2025;21(1):128–42. 10.7150/ijbs.99423. 39744439 10.7150/ijbs.99423 PMC11667812

[3] Castellani G, Buccarelli M, Arasi MB, Rossi S, Pisanu ME, Bellenghi M, et al. BRAF mutations in melanoma: biological aspects, therapeutic implications, and circulating biomarkers. Cancers (Basel). 2023. 10.3390/cancers15164026. 37627054 10.3390/cancers15164026 PMC10452867

[4] Al-Masri M, Al-Shobaki T, Al-Najjar H, Iskanderian R, Younis E, Abdallah N, et al. BRAF V600E mutation in papillary thyroid carcinoma: it’s relation to clinical features and oncologic outcomes in a single cancer centre experience. Endocr Connect. 2021;10(12):1531–7. 10.1530/ec-21-0410. 34734568 10.1530/EC-21-0410 PMC8679880

[5] Morris V, Overman MJ, Jiang ZQ, Garrett C, Agarwal S, Eng C, et al. Progression-free survival remains poor over sequential lines of systemic therapy in patients with BRAF-mutated colorectal cancer. Clin Colorectal Cancer. 2014;13(3):164–71. 10.1016/j.clcc.2014.06.001. 25069797 10.1016/j.clcc.2014.06.001 PMC4266576

[6] Gutzmer R, Stroyakovskiy D, Gogas H, Robert C, Lewis K, Protsenko S, et al. Atezolizumab, vemurafenib, and cobimetinib as first-line treatment for unresectable advanced BRAF V600 mutation-positive melanoma (IMspire150): primary analysis of the randomised, double-blind, placebo-controlled, phase 3 trial. Lancet (London, England). 2020;395(10240):1835–44. 10.1016/s0140-6736(20)30934-x. 32534646 10.1016/S0140-6736(20)30934-X

[7] Syeda MM, Wiggins JM, Corless BC, Long GV, Flaherty KT, Schadendorf D, et al. Circulating tumour DNA in patients with advanced melanoma treated with dabrafenib or dabrafenib plus trametinib: a clinical validation study. Lancet Oncol. 2021;22(3):370–80. 10.1016/s1470-2045(20)30726-9. 33587894 10.1016/S1470-2045(20)30726-9 PMC8034833

[8] Farlow JL, McCrary HC, Sipos JA, Phay JE, Konda B, Agrawal A. Neoadjuvant dabrafenib and trametinib for functional organ preservation in recurrent BRAF V600E-mutated papillary thyroid cancer. Oral Oncol. 2023;147:106625. 10.1016/j.oraloncology.2023.106625. 37948895 10.1016/j.oraloncology.2023.106625

[9] Chen D, Su X, Zhu L, Jia H, Han B, Chen H, et al. Papillary thyroid cancer organoids harboring BRAF V600E mutation reveal potentially beneficial effects of BRAF inhibitor-based combination therapies. J Transl Med. 2023;21(1):9. 10.1186/s12967-022-03848-z. 36624452 10.1186/s12967-022-03848-z PMC9827684

[10] Middleton G, Yang Y, Campbell CD, André T, Atreya CE, Schellens JHM, et al. BRAF -mutant transcriptional subtypes predict outcome of combined BRAF, MEK, and EGFR blockade with dabrafenib, trametinib, and panitumumab in patients with colorectal cancer. Clin Cancer Res Official J Am Assoc Cancer Res. 2020;26(11):2466–76. 10.1158/1078-0432.Ccr-19-3579. 10.1158/1078-0432.CCR-19-3579 PMC8194012 32047001

[11] Tabernero J, Grothey A, Van Cutsem E, Yaeger R, Wasan H, Yoshino T, et al. Encorafenib plus cetuximab as a new standard of care for previously treated BRAF V600E-mutant metastatic colorectal cancer: updated survival results and subgroup analyses from the BEACON study. J Clin Oncol. 2021;39(4):273–84. 10.1200/jco.20.02088. 33503393 10.1200/JCO.20.02088 PMC8078423

[12] Planchard D, Besse B, Groen HJM, Hashemi SMS, Mazieres J, Kim TM, et al. Phase 2 study of dabrafenib plus trametinib in patients with BRAF V600E-mutant metastatic NSCLC: updated 5-year survival rates and genomic analysis. J Thorac Oncol Official Pub Int Assoc Study Lung Cancer. 2022;17(1):103–15. 10.1016/j.jtho.2021.08.011. 10.1016/j.jtho.2021.08.011 34455067

[13] Dummer R, Long GV, Robert C, Tawbi HA, Flaherty KT, Ascierto PA, et al. Randomized phase III trial evaluating spartalizumab plus dabrafenib and trametinib for BRAF V600-mutant unresectable or metastatic melanoma. J Clin Oncol. 2022;40(13):1428–38. 10.1200/jco.21.01601. 35030011 10.1200/JCO.21.01601 PMC9061149

[14] Jung T, Haist M, Kuske M, Grabbe S, Bros M. Immunomodulatory properties of BRAF and MEK inhibitors used for melanoma therapy-paradoxical ERK activation and beyond. Int J Mol Sci. 2021. 10.3390/ijms22189890. 34576054 10.3390/ijms22189890 PMC8469254

[15] Verma V, Jafarzadeh N, Boi S, Kundu S, Jiang Z, Fan Y, et al. MEK inhibition reprograms CD8 + T lymphocytes into memory stem cells with potent antitumor effects. Nat Immunol. 2021;22(1):53–66. 10.1038/s41590-020-00818-9. 33230330 10.1038/s41590-020-00818-9 PMC10081014

[16] Dummer R, Welti M, Ramelyte E. The role of triple therapy and therapy sequence in treatment of BRAF-mutant metastatic melanoma. Response to overall survival with first-line atezolizumab in combination with vemurafenib and cobimetinib in BRAFV600 mutation-positive advanced melanoma (IMspire150): second interim analysis of a multicentre, randomised, phase 3 study. J Transl Med. 2023;21(1):529. 10.1186/s12967-023-04391-1. 37543586 10.1186/s12967-023-04391-1 PMC10403899

[17] Paik A, Purawarga Matada GS, Ghara A, Pal R, Pavani G, Kamurthy H. BRAF inhibitors in melanoma: structural insights, therapeutic resistance, and biological evaluation of quinazoline derivatives. Eur J Med Chem. 2025;296:117866. 10.1016/j.ejmech.2025.117866. 40541114 10.1016/j.ejmech.2025.117866

[18] Kosnopfel C, Sinnberg T, Mane S, Dongo M, Garbe C, Niessner H. Combinatorial ERK inhibition enhances MAPK pathway suppression in BRAF-mutant melanoma. Int J Mol Sci. 2025. 10.3390/ijms26199794. 41097061 10.3390/ijms26199794 PMC12524744

[19] Harada K, Yuki S, Kawamoto Y, Nakamura T, Kaneko S, Ishida K, et al. Anti-epidermal growth factor receptor treatment for patients with Neo RAS wild-type metastatic colorectal cancer: a case report of two cases. Ther Adv Med Oncol. 2023;15:17588359231216090. 10.1177/17588359231216090. 38033418 10.1177/17588359231216090 PMC10685759

[20] Stagno A, Vari S, Annovazzi A, Anelli V, Russillo M, Cognetti F, et al. Case report: rechallenge with BRAF and MEK inhibitors in metastatic melanoma: a further therapeutic option in salvage setting? Front Oncol. 2021;11:645008. 10.3389/fonc.2021.645008. 34136385 10.3389/fonc.2021.645008 PMC8202400

[21] Bai J, Wan Z, Zhou W, Wang L, Lou W, Zhang Y, et al. Global trends and emerging insights in BRAF and MEK inhibitor resistance in melanoma: a bibliometric analysis. Front Mol Biosci. 2025;12:1538743. 10.3389/fmolb.2025.1538743. 39897423 10.3389/fmolb.2025.1538743 PMC11782018

[22] Yaeger R, Yao Z, Hyman DM, Hechtman JF, Vakiani E, Zhao H, et al. Mechanisms of acquired resistance to BRAF V600E inhibition in colon cancers converge on RAF dimerization and are sensitive to its inhibition. Cancer Res. 2017;77(23):6513–23. 10.1158/0008-5472.Can-17-0768. 28951457 10.1158/0008-5472.CAN-17-0768 PMC5712250

[23] Yao Z, Yaeger R, Rodrik-Outmezguine VS, Tao A, Torres NM, Chang MT, et al. Tumours with class 3 BRAF mutants are sensitive to the inhibition of activated RAS. Nature. 2017;548(7666):234–8. 10.1038/nature23291. 28783719 10.1038/nature23291 PMC5648058

[24] Patel H, Mishra R, Yacoub N, Alanazi S, Kilroy MK, Garrett JT. IGF1R/IR mediates resistance to BRAF and MEK inhibitors in BRAF-mutant melanoma. Cancers (Basel). 2021. 10.3390/cancers13225863. 34831014 10.3390/cancers13225863 PMC8616282

[25] Ji Z, Njauw CN, Guhan S, Kumar R, Reddy B, Rajadurai A, et al. Loss of ACK1 Upregulates EGFR and Mediates Resistance to BRAF Inhibition. J Invest Dermatol. 2021;141(5):1317-1324.e1311. 10.1016/j.jid.2020.06.041. 33159968 10.1016/j.jid.2020.06.041

[26] Ndoja A, Rose CM, Lin E, Reja R, Petrovic J, Kummerfeld S, et al. COP1 deficiency in BRAF V600E melanomas confers resistance to inhibitors of the MAPK pathway. Cells. 2025. 10.3390/cells14130975. 40643496 10.3390/cells14130975 PMC12249101

[27] Mologni L, Costanza M, Sharma GG, Viltadi M, Massimino L, Citterio S, et al. Concomitant BCORL1 and BRAF mutations in vemurafenib-resistant melanoma cells. Neoplasia. 2018;20(5):467–77. 10.1016/j.neo.2018.02.009. 29605720 10.1016/j.neo.2018.02.009 PMC5915992

[28] Zhai Z, Yamauchi T, Sandoval K, Villarreal K, Kwong MWC, Swanson EJ, et al. Downregulated ALDH2 contributes to tumor progression and targeted therapy resistance in human metastatic melanoma cells. Cells. 2025. 10.3390/cells14120913. 40558540 10.3390/cells14120913 PMC12191128

[29] Kang Y, Ji Z, Li H, Tsao H. Divergent BRAF inhibitor resistance mechanisms revealed through epigenetic mapping. J Invest Dermatol. 2023;143(5):842-853.e846. 10.1016/j.jid.2022.03.039. 36529262 10.1016/j.jid.2022.03.039

[30] Celesia A, Notaro A, Franzò M, Lauricella M, D’Anneo A, Carlisi D, et al. The histone deacetylase inhibitor ITF2357 (Givinostat) targets oncogenic BRAF in melanoma cells and promotes a switch from pro-survival autophagy to apoptosis. Biomedicines. 2022. 10.3390/biomedicines10081994. 36009541 10.3390/biomedicines10081994 PMC9405675

[31] Mitra S, Lauss M, Cabrita R, Choi J, Zhang T, Isaksson K, et al. Analysis of DNA methylation patterns in the tumor immune microenvironment of metastatic melanoma. Mol Oncol. 2020;14(5):933–50. 10.1002/1878-0261.12663. 32147909 10.1002/1878-0261.12663 PMC7191190

[32] Liu Y, Ruan H, Lu F, Peng H, Luan W. miR-224-5p acts as a tumour suppressor and reverses the resistance to BRAF inhibitor in melanoma through directly targeting PAK4 to block the MAPK pathway. Pathol Res Pract. 2023;249:154772. 10.1016/j.prp.2023.154772. 37611431 10.1016/j.prp.2023.154772

[33] Soumoy L, Genbauffe A, Sant’Angelo D, Everaert M, Mukeba-Harchies L, Sarry JE, et al. Therapeutic potential of glutaminase inhibition targeting metabolic adaptations in resistant melanomas to targeted therapy. Int J Mol Sci. 2025. 10.3390/ijms26178241. 40943167 10.3390/ijms26178241 PMC12428566

[34] Kharouf N, Flanagan TW, Hassan SY, Shalaby H, Khabaz M, Hassan SL, et al. Tumor microenvironment as a therapeutic target in melanoma treatment. Cancers (Basel). 2023. 10.3390/cancers15123147. 37370757 10.3390/cancers15123147 PMC10296288

[35] Khan ZM, Rossello-Martinez A, Mak M. Impact of mechanical and architectural signals in the tumor microenvironment on melanoma. Adv Healthc Mater. 2025. 10.1002/adhm.202501759.40914820 10.1002/adhm.202501759

[36] Smith MP, Sanchez-Laorden B, O’Brien K, Brunton H, Ferguson J, Young H, et al. The immune microenvironment confers resistance to MAPK pathway inhibitors through macrophage-derived TNFα. Cancer Discov. 2014;4(10):1214–29. 10.1158/2159-8290.Cd-13-1007. 25256614 10.1158/2159-8290.CD-13-1007 PMC4184867

[37] Ponzone L, Audrito V, Landi C, Moiso E, Levra Levron C, Ferrua S, et al. RICTOR/mTORC2 downregulation in BRAF V600E melanoma cells promotes resistance to BRAF/MEK inhibition. Mol Cancer. 2024;23(1):105. 10.1186/s12943-024-02010-1. 38755661 10.1186/s12943-024-02010-1 PMC11097536

[38] Lubrano S, Cervantes-Villagrana RD, Faraji F, Ramirez S, Sato K, Adame-Garcia SR, et al. FAK inhibition combined with the RAF-MEK clamp avutometinib overcomes resistance to targeted and immune therapies in BRAF V600E melanoma. Cancer Cell. 2025;43(3):428-445.e426. 10.1016/j.ccell.2025.02.001. 40020669 10.1016/j.ccell.2025.02.001 PMC11903146

[39] Yang S, Im SH, Chung JY, Lee J, Lee KH, Kang YK, et al. An antibody-CRISPR/Cas conjugate platform for target-specific delivery and gene editing in cancer. Adv Sci. 2024;11(21):e2308763. 10.1002/advs.202308763. 10.1002/advs.202308763 PMC11151032 38552157

[40] Phillipps J, Nassief G, Morecroft R, Adeyelu T, Elliott A, Abdulla F, et al. Efficacy of PARP inhibitor therapy after targeted BRAF/MEK failure in advanced melanoma. NPJ Precis Oncol. 2024;8(1):187. 10.1038/s41698-024-00684-w. 39232122 10.1038/s41698-024-00684-w PMC11374802

[41] Yang J, Zeng X, Pei J, Su Z, Liu Q, Zhang Y, et al. Protocatechuic aldehyde sensitizes BRAF-mutant melanoma cells to temozolomide through inducing FANCD2 degradation. Med Oncol (Northwood, London, England). 2025;42(2):48. 10.1007/s12032-025-02601-y. 10.1007/s12032-025-02601-y 39824992

[42] Delyon J, Becherirat S, Roger A, Bernard-Cacciarella M, Reger De Moura C, Louveau B, et al. PDE4D drives rewiring of the MAPK pathway in BRAF-mutated melanoma resistant to MAPK inhibitors. Cell Commun Signal. 2024;22(1):559. 10.1186/s12964-024-01941-y. 39574163 10.1186/s12964-024-01941-y PMC11580363

[43] Borbényi-Galambos K, Erdélyi K, Ditrói T, Jurányi EP, Szántó N, Szatmári R, et al. Realigned transsulfuration drives BRAF-V600E-targeted therapy resistance in melanoma. Cell Metab. 2025;37(5):1171-1188.e1179. 10.1016/j.cmet.2025.01.021. 40037361 10.1016/j.cmet.2025.01.021

[44] Devinat M, Thevenard-Devy J, Ghilane F, Devy J, Chazee L, Terryn C, et al. Xanthohumol sensitizes melanoma cells to vemurafenib by lowering membrane cholesterol and increasing membrane fluidity. Int J Mol Sci. 2025. 10.3390/ijms26052290. 40076912 10.3390/ijms26052290 PMC11901044

[45] Tiffen J, Gallagher SJ, Filipp F, Gunatilake D, Emran AA, Cullinane C, et al. EZH2 Cooperates with DNA Methylation to Downregulate Key Tumor Suppressors and IFN Gene Signatures in Melanoma. J Invest Dermatol. 2020;140(12):2442-2454.e2445. 10.1016/j.jid.2020.02.042. 32360600 10.1016/j.jid.2020.02.042

[46] Wu L, Li K, Lin W, Liu J, Qi Q, Shen G, et al. Long noncoding RNA LINC01291 promotes the aggressive properties of melanoma by functioning as a competing endogenous RNA for microRNA-625-5p and subsequently increasing IGF-1R expression. Cancer Gene Ther. 2022;29(3–4):341–57. 10.1038/s41417-021-00313-9. 33674778 10.1038/s41417-021-00313-9 PMC8940622

[47] Becker AL, Indra AK. Oxidative stress in melanoma: beneficial antioxidant and pro-oxidant therapeutic strategies. Cancers (Basel). 2023. 10.3390/cancers15113038. 37297001 10.3390/cancers15113038 PMC10252072

[48] Wang L, He S, Liu R, Xue Y, Quan Y, Shi R, et al. A pH/ROS dual-responsive system for effective chemoimmunotherapy against melanoma via remodeling tumor immune microenvironment. Acta Pharm Sin B. 2024;14(5):2263–80. 10.1016/j.apsb.2023.12.001. 38799639 10.1016/j.apsb.2023.12.001 PMC11119573

[49] Groenland SL, Janssen JM, Nijenhuis CM, de Vries N, Rosing H, Wilgenhof S, et al. Exposure-response analyses of BRAF- and MEK-inhibitors dabrafenib plus trametinib in melanoma patients. Cancer Chemother Pharmacol. 2023;91(6):447–56. 10.1007/s00280-023-04517-8. 36947208 10.1007/s00280-023-04517-8

[50] Galati D, Zanotta S, Capone M, Madonna G, Mallardo D, Romanelli M, et al. Potential clinical implications of CD4 + CD26 high T cells for nivolumab treated melanoma patients. J Transl Med. 2023;21(1):318. 10.1186/s12967-023-04184-6. 37170241 10.1186/s12967-023-04184-6 PMC10176780

[51] Boz Er AB, Sheldrake HM, Sutherland M. Overcoming vemurafenib resistance in metastatic melanoma: targeting integrins to improve treatment efficacy. Int J Mol Sci. 2024. 10.3390/ijms25147946. 39063187 10.3390/ijms25147946 PMC11277089

[52] Fragale A, Stellacci E, Romagnoli G, Licursi V, Parlato S, Canini I, et al. Reversing vemurafenib-resistance in primary melanoma cells by combined romidepsin and type I IFN treatment through blocking of tumorigenic signals and induction of immunogenic effects. Int J Cancer. 2023;153(5):1080–95. 10.1002/ijc.34602. 37293858 10.1002/ijc.34602

[53] Diefenbach RJ, Lee JH, Menzies AM, Carlino MS, Long GV, Saw RPM, et al. Design and testing of a custom melanoma next generation sequencing panel for analysis of circulating tumor DNA. Cancers (Basel). 2020. 10.3390/cancers12082228. 32785074 10.3390/cancers12082228 PMC7465941

[54] Fietz S, Diekmann E, de Vos L, Zarbl R, Hunecke A, Glosch AK, et al. Circulating cell-free SHOX2 DNA methylation is a predictive, prognostic, and monitoring biomarker in adjuvant and palliative anti-PD-1-treated melanoma. Clin Chem. 2024;70(3):516–27. 10.1093/clinchem/hvad230. 38300881 10.1093/clinchem/hvad230

[55] Valpione S, Gremel G, Mundra P, Middlehurst P, Galvani E, Girotti MR, et al. Plasma total cell-free DNA (cfDNA) is a surrogate biomarker for tumour burden and a prognostic biomarker for survival in metastatic melanoma patients. Eur J (Oxford, England : 1990). 2018;88:1–9. 10.1016/j.ejca.2017.10.029. 10.1016/j.ejca.2017.10.029 PMC5769519 29175734

[56] Redondo-Muñoz M, Rodriguez-Baena FJ, Aldaz P, Caballé-Mestres A, Moncho-Amor V, Otaegi-Ugartemendia M, et al. Metabolic rewiring induced by ranolazine improves melanoma responses to targeted therapy and immunotherapy. Nat Metab. 2023;5(9):1544–62. 10.1038/s42255-023-00861-4. 37563469 10.1038/s42255-023-00861-4 PMC10513932

[57] Mimitou EP, Lareau CA, Chen KY, Zorzetto-Fernandes AL, Hao Y, Takeshima Y, et al. Scalable, multimodal profiling of chromatin accessibility, gene expression and protein levels in single cells. Nat Biotechnol. 2021;39(10):1246–58. 10.1038/s41587-021-00927-2. 34083792 10.1038/s41587-021-00927-2 PMC8763625

[58] Wang H, Yang Y, Zhang J, Chen W, Dai J, Li C, et al. Integrating single-cell RNA sequencing and artificial intelligence for multitargeted drug design for combating resistance in liver cancer. NPJ Precision Oncol. 2025;9(1):309. 10.1038/s41698-025-00952-3. 10.1038/s41698-025-00952-3 PMC12405525 40897921

[59] Eroglu Z, Chen YA, Smalley I, Li J, Markowitz JK, Brohl AS, et al. Combined BRAF, MEK, and heat-shock protein 90 inhibition in advanced BRAF V600-mutant melanoma. Cancer. 2024;130(2):232–43. 10.1002/cncr.35029. 37776537 10.1002/cncr.35029

[60] Nagasawa I, Koido M, Tani Y, Tsukahara S, Kunimasa K, Tomida A. Disrupting ATF4 expression mechanisms provides an effective strategy for BRAF-targeted melanoma therapy. iScience. 2020;23(4):101028. 10.1016/j.isci.2020.101028. 32283529 10.1016/j.isci.2020.101028 PMC7155235

[61] Dankner M, Rose AAN, Rajkumar S, Siegel PM, Watson IR. Classifying BRAF alterations in cancer: new rational therapeutic strategies for actionable mutations. Oncogene. 2018;37(24):3183–99. 10.1038/s41388-018-0171-x. 29540830 10.1038/s41388-018-0171-x

[62] Haque M, Koski KG, Scott ME. Maternal gastrointestinal nematode infection up-regulates expression of genes associated with long-term potentiation in perinatal brains of uninfected developing pups. Sci Rep. 2019;9(1):4165. 10.1038/s41598-019-40729-w. 30862816 10.1038/s41598-019-40729-w PMC6414690

[63] Oya Y, Kuroda H, Nakada T, Takahashi Y, Sakakura N, Hida T. Efficacy of immune checkpoint inhibitor monotherapy for advanced non-small-cell lung cancer with ALK rearrangement. Int J Mol Sci. 2020. 10.3390/ijms21072623. 32283823 10.3390/ijms21072623 PMC7178012

[64] Zhao C, Jia Z. [Clinical value of BRAF V600E in thyroid carcinoma and the effect of telomerase reverse transcriptase promoter mutations]. Sheng Wu Yi Xue Gong Cheng Xue Za Zhi J Biomed Eng. 2019;36(2):338–42. 10.7507/1001-5515.201804002. 10.7507/1001-5515.201804002 PMC9929895 31016954

[65] Marranci A, Jiang Z, Vitiello M, Guzzolino E, Comelli L, Sarti S, et al. The landscape of BRAF transcript and protein variants in human cancer. Mol Cancer. 2017;16(1):85. 10.1186/s12943-017-0645-4. 28454577 10.1186/s12943-017-0645-4 PMC5410044

[66] Dong X, Akuetteh PDP, Song J, Ni C, Jin C, Li H, et al. Major vault protein (MVP) associated with BRAF V600E mutation is an immune microenvironment-related biomarker promoting the progression of papillary thyroid cancer via MAPK/ERK and PI3K/AKT pathways. Front Cell Dev Biol. 2021;9:688370. 10.3389/fcell.2021.688370. 35433709 10.3389/fcell.2021.688370 PMC9009514

[67] Maloney RC, Zhang M, Jang H, Nussinov R. The mechanism of activation of monomeric B-Raf V600E. Comput Struct Biotechnol J. 2021;19:3349–63. 10.1016/j.csbj.2021.06.007. 34188782 10.1016/j.csbj.2021.06.007 PMC8215184

[68] Yao Z, Gao Y, Su W, Yaeger R, Tao J, Na N, et al. RAF inhibitor PLX8394 selectively disrupts BRAF dimers and RAS-independent BRAF-mutant-driven signaling. Nat Med. 2019;25(2):284–91. 10.1038/s41591-018-0274-5. 30559419 10.1038/s41591-018-0274-5 PMC6404779

[69] Karoulia Z, Wu Y, Ahmed TA, Xin Q, Bollard J, Krepler C, et al. An integrated model of RAF inhibitor action predicts inhibitor activity against oncogenic BRAF signaling. Cancer Cell. 2016;30(3):485–98. 10.1016/j.ccell.2016.06.024. 27523909 10.1016/j.ccell.2016.06.024 PMC5021590

[70] Cao M, Zhang R, Hong A, Ye S, Qiu Z, Li D, et al. CEP55 promotes acral melanoma progression via MAPK pathway and predicts survival following immunotherapy. Oncol Res. 2025;33(9):2507–27. 10.32604/or.2025.064780. 40918457 10.32604/or.2025.064780 PMC12408855

[71] Papadopoulou K, Mystakidou CM, Papadopoulou E, Varsamis N, Koulouris C, Theodorou V, et al. Melanoma: BRAFi rechallenge. Medicina (Kaunas). 2023. 10.3390/medicina59050975. 37241207 10.3390/medicina59050975 PMC10220981

[72] Babačić H, Eriksson H, Pernemalm M. Plasma proteome alterations by MAPK inhibitors in BRAF V600 -mutated metastatic cutaneous melanoma. Neoplasia (New York, NY). 2021;23(8):783–91. 10.1016/j.neo.2021.06.002. 10.1016/j.neo.2021.06.002 PMC8274243 34246984

[73] Beck L, Harel M, Yu S, Markovits E, Boursi B, Markel G, et al. Clinical proteomics of metastatic melanoma reveals profiles of organ specificity and treatment resistance. Clin Cancer Res. 2021;27(7):2074–86. 10.1158/1078-0432.Ccr-20-3752. 33446566 10.1158/1078-0432.CCR-20-3752

[74] Embaby A, Huijberts SCFA, Wang L, de Leite Oliveira R, Rosing H, Nuijen B, et al. A proof-of-concept study of sequential treatment with the HDAC inhibitor vorinostat following BRAF and MEK inhibitors in BRAFV600-mutated melanoma. Clin Cancer Res Official J Am Assoc Cancer Res. 2024;30(15):3157–66. 10.1158/1078-0432.Ccr-23-3171. 10.1158/1078-0432.CCR-23-3171 38739109

[75] Barbato MI, Nashed J, Bradford D, Ren Y, Khasar S, Miller CP, et al. FDA approval summary: dabrafenib in combination with trametinib for BRAFV600E mutation-positive low-grade glioma. Clin Cancer Res. 2024;30(2):263–8. 10.1158/1078-0432.Ccr-23-1503. 37610803 10.1158/1078-0432.CCR-23-1503 PMC10841289

[76] Mazieres J, Cropet C, Montané L, Barlesi F, Souquet PJ, Quantin X, et al. Vemurafenib in non-small-cell lung cancer patients with BRAF V600 and BRAF nonV600 mutations. Ann Oncol. 2020;31(2):289–94. 10.1016/j.annonc.2019.10.022. 31959346 10.1016/j.annonc.2019.10.022

[77] Maniar R, Gallitano SM, Husain S, Moazami G, Weiss MJ, Shu CA. Unusual adverse events in a patient with BRAF-mutated non-small cell lung cancer treated with BRAF/MEK inhibition. J National Compr Cancer Network JNCCN. 2023;21(3):232–4. 10.6004/jnccn.2022.7084. 10.6004/jnccn.2022.7084 36758579

[78] Andrews LJ, Thornton ZA, Saincher SS, Yao IY, Dawson S, McGuinness LA, et al. Prevalence of BRAFV600 in glioma and use of BRAF inhibitors in patients with BRAFV600 mutation-positive glioma: systematic review. Neurooncol. 2022;24(4):528–40. 10.1093/neuonc/noab247. 10.1093/neuonc/noab247 PMC8972326 34718782

[79] Planchard D, Kim TM, Mazieres J, Quoix E, Riely G, Barlesi F, et al. Dabrafenib in patients with BRAF(V600E)-positive advanced non-small-cell lung cancer: a single-arm, multicentre, open-label, phase 2 trial. Lancet Oncol. 2016;17(5):642–50. 10.1016/s1470-2045(16)00077-2. 27080216 10.1016/S1470-2045(16)00077-2 PMC5006181

[80] Wen PY, Stein A, van den Bent M, De Greve J, Wick A, de Vos FYFL, et al. Dabrafenib plus trametinib in patients with BRAF V600E -mutant low-grade and high-grade glioma (ROAR): a multicentre, open-label, single-arm, phase 2, basket trial. Lancet Oncol. 2022;23(1):53–64. 10.1016/s1470-2045(21)00578-7. 34838156 10.1016/S1470-2045(21)00578-7

[81] Ascierto PA, Casula M, Bulgarelli J, Pisano M, Piccinini C, Piccin L, et al. Sequential immunotherapy and targeted therapy for metastatic BRAF V600 mutated melanoma: 4-year survival and biomarkers evaluation from the phase II SECOMBIT trial. Nat Commun. 2024;15(1):146. 10.1038/s41467-023-44475-6. 38167503 10.1038/s41467-023-44475-6 PMC10761671

[82] Ambrosini M, Tougeron D, Modest D, Guimbaud R, Kopetz S, Decraecker M, et al. BRAF + EGFR +/- MEK inhibitors after immune checkpoint inhibitors in BRAF V600E mutated and deficient mismatch repair or microsatellite instability high metastatic colorectal cancer. Eur J Cancer (Oxford, England : 1990). 2024;210:114290. 10.1016/j.ejca.2024.114290. 10.1016/j.ejca.2024.114290 39216175

[83] Riely GJ, Smit EF, Ahn MJ, Felip E, Ramalingam SS, Tsao A, et al. Phase II, open-label study of encorafenib plus binimetinib in patients with BRAF V600 -mutant metastatic non-small-cell lung cancer. J Clin Oncol. 2023;41(21):3700–11. 10.1200/jco.23.00774. 37270692 10.1200/JCO.23.00774

[84] Baik C, Cheng ML, Dietrich M, Gray JE, Karim NA. A practical review of encorafenib and binimetinib therapy management in patients with BRAF V600E-mutant metastatic non-small cell lung cancer. Adv Ther. 2024;41(7):2586–605. 10.1007/s12325-024-02839-4. 38698170 10.1007/s12325-024-02839-4 PMC11213720

[85] Oliveira ÉA, Chauhan J, Silva JRD, Carvalho LADC, Dias D, Carvalho DG, et al. TOP1 modulation during melanoma progression and in adaptative resistance to BRAF and MEK inhibitors. Pharmacol Res. 2021;173:105911. 10.1016/j.phrs.2021.105911. 34560251 10.1016/j.phrs.2021.105911 PMC8729257

[86] Shimizu Y, Maruyama K, Suzuki M, Kawachi H, Low SK, Oh-Hara T, et al. Acquired resistance to BRAF inhibitors is mediated by BRAF splicing variants in BRAF V600E mutation-positive colorectal neuroendocrine carcinoma. Cancer Lett. 2022;543:215799. 10.1016/j.canlet.2022.215799. 35724767 10.1016/j.canlet.2022.215799

[87] Bromberger S, Zadorozhna Y, Ressler JM, Holzner S, Nawrocki A, Zila N, et al. Off-targets of BRAF inhibitors disrupt endothelial signaling and vascular barrier function. Life Sci Alliance. 2024. 10.26508/lsa.202402671. 38839106 10.26508/lsa.202402671 PMC11153892

[88] Madej E, Brożyna AA, Adamczyk A, Wronski N, Harazin-Lechowska A, Muzyk A, et al. Vemurafenib and dabrafenib downregulates RIPK4 level. Cancers (Basel). 2023. 10.3390/cancers15030918. 36765875 10.3390/cancers15030918 PMC9913565

[89] Carvalho DG, Kenski JCN, Moreira DA, Rajão MA, Krijgsman O, Furtado C, et al. Resistance to BRAF inhibitors drives melanoma sensitivity to Chk1 inhibition. Pharmacol Res. 2025;217:107797. 10.1016/j.phrs.2025.107797. 40414585 10.1016/j.phrs.2025.107797

[90] Kado S, Komine M. Recent advances in molecular research and treatment for melanoma in Asian populations. Int J Mol Sci. 2025. 10.3390/ijms26115370. 40508177 10.3390/ijms26115370 PMC12154924

[91] Radić M, Vlašić I, Jazvinšćak Jembrek M, Horvat A, Tadijan A, Sabol M, et al. Characterization of vemurafenib-resistant melanoma cell lines reveals novel hallmarks of targeted therapy resistance. Int J Mol Sci. 2022. 10.3390/ijms23179910. 36077308 10.3390/ijms23179910 PMC9455970

[92] Du BX, Lin P, Lin J. EGCG and ECG induce apoptosis and decrease autophagy via the AMPK/mTOR and PI3K/AKT/mTOR pathway in human melanoma cells. Chin J Nat Med. 2022;20(4):290–300. 10.1016/s1875-5364(22)60166-3. 35487599 10.1016/S1875-5364(22)60166-3

[93] Wang B, Zhang W, Zhang G, Kwong L, Lu H, Tan J, et al. Targeting mTOR signaling overcomes acquired resistance to combined BRAF and MEK inhibition in BRAF-mutant melanoma. Oncogene. 2021;40(37):5590–9. 10.1038/s41388-021-01911-5. 34304249 10.1038/s41388-021-01911-5 PMC8445818

[94] Chen L, Pruteanu-Malinici I, Dastur A, Yin X, Frederick D, Sadreyev RI, et al. Transposon mediated functional genomic screening for BRAF inhibitor resistance reveals convergent Hippo and MAPK pathway activation events. Sci Rep. 2025;15(1):3048. 10.1038/s41598-025-86694-5. 39856157 10.1038/s41598-025-86694-5 PMC11760944

[95] Park S, Ryu WJ, Kim TY, Hwang Y, Han HJ, Lee JD, et al. Overcoming BRAF and CDK4/6 inhibitor resistance by inhibiting MAP3K3-dependent protection against YAP lysosomal degradation. Exp Mol Med. 2024;56(4):987–1000. 10.1038/s12276-024-01210-5. 38622197 10.1038/s12276-024-01210-5 PMC11059244

[96] Valcikova B, Vadovicova N, Smolkova K, Zacpalova M, Krejci P, Lee S, et al. eIF4F controls ERK MAPK signaling in melanomas with BRAF and NRAS mutations. Proc Natl Acad Sci U S A. 2024;121(44):e2321305121. 10.1073/pnas.2321305121. 39436655 10.1073/pnas.2321305121 PMC11536119

[97] Onaga R, Enokida T, Sakashita S, Tanaka N, Hoshi Y, Kishida T, et al. Concordance of BRAF V600E mutation between immunohistochemistry and genomic testing for thyroid cancer. Int J Clin Oncol. 2025;30(6):1143–51. 10.1007/s10147-025-02760-y. 40210835 10.1007/s10147-025-02760-y

[98] Grillo F, Paudice M, Pigozzi S, Dono M, Lastraioli S, Lugaresi M, et al. BRAF V600E immunohistochemistry can reliably substitute BRAF molecular testing in the Lynch syndrome screening algorithm in colorectal cancer. Histopathology. 2024;84(5):877–87. 10.1111/his.15133. 38173291 10.1111/his.15133

[99] Rusu S, Verocq C, Trepant AL, Maris C, De Nève N, Blanchard O, et al. Immunohistochemistry as an accurate tool for the assessment of BRAF V600E and TP53 mutations in primary and metastatic melanoma. Mol Clin Oncol. 2021;15(6):270. 10.3892/mco.2021.2432. 34790354 10.3892/mco.2021.2432 PMC8591695

[100] Zhou Y, Li J, Zhou C, Hou J, Wang J, Lan T, et al. Deep learning-guided quantitative analysis establishes optimized BRAF V600E immunohistochemical criteria for colorectal cancer: a multiplatform validation study. Lab Invest. 2025;105(11):104215. 10.1016/j.labinv.2025.104215. 40683333 10.1016/j.labinv.2025.104215

[101] Tripathi R, Liu Z, Jain A, Lyon A, Meeks C, Richards D, et al. Combating acquired resistance to MAPK inhibitors in melanoma by targeting Abl1/2-mediated reactivation of MEK/ERK/MYC signaling. Nat Commun. 2020;11(1):5463. 10.1038/s41467-020-19075-3. 33122628 10.1038/s41467-020-19075-3 PMC7596241

[102] Mondru AK, Wilkinson B, Aljasir MA, Alrumayh A, Greaves G, Emmett M, et al. The ERK5 pathway in BRAFV600E melanoma cells plays a role in development of acquired resistance to dabrafenib but not vemurafenib. FEBS Lett. 2024;598(16):2011–27. 10.1002/1873-3468.14960. 38977937 10.1002/1873-3468.14960

[103] Kot M, Simiczyjew A, Wądzyńska J, Ziętek M, Matkowski R, Nowak D. Characterization of two melanoma cell lines resistant to BRAF/MEK inhibitors (vemurafenib and cobimetinib). Cell Commun Signal. 2024;22(1):410. 10.1186/s12964-024-01788-3. 39175042 10.1186/s12964-024-01788-3 PMC11342534

[104] Samarkina A, Youssef MK, Ostano P, Ghosh S, Ma M, Tassone B, et al. Androgen receptor is a determinant of melanoma targeted drug resistance. Nat Commun. 2023;14(1):6498. 10.1038/s41467-023-42239-w. 37838724 10.1038/s41467-023-42239-w PMC10576812

[105] Ahronian LG, Sennott EM, Van Allen EM, Wagle N, Kwak EL, Faris JE, et al. Clinical acquired resistance to RAF inhibitor combinations in BRAF-mutant colorectal cancer through MAPK pathway alterations. Cancer Discov. 2015;5(4):358–67. 10.1158/2159-8290.Cd-14-1518. 25673644 10.1158/2159-8290.CD-14-1518 PMC4390490

[106] Sudhakar N, Yan L, Qiryaqos F, Engstrom LD, Laguer J, Calinisan A, et al. The SOS1 inhibitor MRTX0902 blocks KRAS activation and demonstrates antitumor activity in cancers dependent on KRAS nucleotide loading. Mol Cancer Ther. 2024;23(10):1418–30. 10.1158/1535-7163.Mct-23-0870. 38904222 10.1158/1535-7163.MCT-23-0870 PMC11443210

[107] Chun-On P, Hinchie AM, Beale HC, Gil Silva AA, Rush E, Sander C, et al. TPP1 promoter mutations cooperate with TERT promoter mutations to lengthen telomeres in melanoma. Science (New York, NY). 2022;378(6620):664–8. 10.1126/science.abq0607. 10.1126/science.abq0607 PMC10590476 36356143

[108] Yu J, Yu J, Wu X, Guo Q, Yin T, Cheng Z, et al. The TERT copy number gain is sensitive to telomerase inhibitors in human melanoma. Clin Sci (London, England : 1979). 2020;134(2):193–205. 10.1042/cs20190890. 10.1042/CS20190890 31919521

[109] Shaughnessy M, Njauw CN, Artomov M, Tsao H. Classifying melanoma by TERT promoter mutational status. J Invest Dermatol. 2020;140(2):390-394.e391. 10.1016/j.jid.2019.06.149. 31425705 10.1016/j.jid.2019.06.149 PMC6983338

[110] Zhang J, Zhang F, Porter KI, Dakup PP, Wang S, Robertson GP, et al. Telomere dysfunction in *Tert* knockout mice delays *Braf* V600E -induced melanoma development. Int J Cancer. 2024;154(3):548–60. 10.1002/ijc.34713. 37727982 10.1002/ijc.34713 PMC10840707

[111] Delyon J, Vallet A, Bernard-Cacciarella M, Kuzniak I, de Reger Moura C, Louveau B, et al. TERT expression induces resistance to BRAF and MEK inhibitors in BRAF-mutated melanoma in vitro. Cancers (Basel). 2023. 10.3390/cancers15112888. 37296851 10.3390/cancers15112888 PMC10251862

[112] Tan J, Liu R, Zhu G, Umbricht CB, Xing M. TERT promoter mutation determines apoptotic and therapeutic responses of BRAF -mutant cancers to BRAF and MEK inhibitors: achilles heel. Proc Natl Acad Sci U S A. 2020;117(27):15846–51. 10.1073/pnas.2004707117. 32561648 10.1073/pnas.2004707117 PMC7355024

[113] Bugide S, Parajuli KR, Chava S, Pattanayak R, Manna DLD, Shrestha D, et al. Loss of HAT1 expression confers BRAFV600E inhibitor resistance to melanoma cells by activating MAPK signaling via IGF1R. Oncogenesis. 2020;9(5):44. 10.1038/s41389-020-0228-x. 32371878 10.1038/s41389-020-0228-x PMC7200761

[114] Aya F, Lanuza-Gracia P, González-Pérez A, Bonnal S, Mancini E, López-Bigas N, et al. Genomic deletions explain the generation of alternative BRAF isoforms conferring resistance to MAPK inhibitors in melanoma. Cell Rep. 2024;43(4):114048. 10.1016/j.celrep.2024.114048. 38614086 10.1016/j.celrep.2024.114048

[115] Gouda MA, Polivka J, Huang HJ, Treskova I, Pivovarcikova K, Fikrle T, et al. Ultrasensitive detection of BRAF mutations in circulating tumor DNA of non-metastatic melanoma. ESMO Open. 2021;7(1):100357. 10.1016/j.esmoop.2021.100357. 34942440 10.1016/j.esmoop.2021.100357 PMC8695283

[116] Gonzalez-Cao M, Mayo de Las Casas C, Oramas J, Berciano-Guerrero MA, de la Cruz L, Cerezuela P, et al. Intermittent BRAF inhibition in advanced BRAF mutated melanoma results of a phase II randomized trial. NatCommun. 2021;12(1):7008. 10.1038/s41467-021-26572-6. 10.1038/s41467-021-26572-6 PMC8636498 34853302

[117] Chu Z, Gu L, Hu Y, Zhang X, Li M, Chen J, et al. STAG2 regulates interferon signaling in melanoma via enhancer loop reprogramming. Nat Commun. 2022;13(1):1859. 10.1038/s41467-022-29541-9. 35388001 10.1038/s41467-022-29541-9 PMC8986786

[118] Shen CH, Kim SH, Trousil S, Frederick DT, Piris A, Yuan P, et al. Loss of cohesin complex components STAG2 or STAG3 confers resistance to BRAF inhibition in melanoma. Nat Med. 2016;22(9):1056–61. 10.1038/nm.4155. 27500726 10.1038/nm.4155 PMC5014622

[119] Lu H, Liu S, Zhang G, Bin Wu, Zhu Y, Frederick DT, et al. PAK signalling drives acquired drug resistance to MAPK inhibitors in BRAF-mutant melanomas. Nature. 2017;550(7674):133–6. 10.1038/nature24040. 28953887 10.1038/nature24040 PMC5891348

[120] Nangia V, Ashraf H, Marikar N, Passanisi VJ, Ill CR, Spencer SL. MAPK and mTORC1 signaling converge to drive cyclin D1 protein production to enable cell cycle reentry in melanoma persister cells. Sci Signal. 2025;18(902):eadw3231. 10.1126/scisignal.adw3231. 40892895 10.1126/scisignal.adw3231 PMC13198176

[121] Ciccone V, Terzuoli E, Ristori E, Filippelli A, Ziche M, Morbidelli L, et al. ALDH1A1 overexpression in melanoma cells promotes tumor angiogenesis by activating the IL‑8/Notch signaling cascade. Int J Mol Med. 2022. 10.3892/ijmm.2022.5155. 35656893 10.3892/ijmm.2022.5155 PMC9186295

[122] Dinavahi SS, Gowda R, Gowda K, Bazewicz CG, Chirasani VR, Battu MB, et al. Development of a novel multi-isoform ALDH inhibitor effective as an antimelanoma agent. Mol Cancer Ther. 2020;19(2):447–59. 10.1158/1535-7163.Mct-19-0360. 31754071 10.1158/1535-7163.MCT-19-0360 PMC10763724

[123] Ciccone V, Simonis V, Del Gaudio C, Cucini C, Ziche M, Morbidelli L, et al. ALDH1A1 confers resistance to RAF/MEK inhibitors in melanoma cells by maintaining stemness phenotype and activating PI3K/AKT signaling. Biochem Pharmacol. 2024;224:116252. 10.1016/j.bcp.2024.116252. 38701866 10.1016/j.bcp.2024.116252

[124] Lu Y, Travnickova J, Badonyi M, Rambow F, Coates A, Khan Z, et al. ALDH1A3-acetaldehyde metabolism potentiates transcriptional heterogeneity in melanoma. Cell Rep. 2024;43(7):114406. 10.1016/j.celrep.2024.114406. 38963759 10.1016/j.celrep.2024.114406 PMC11290356

[125] Muraro E, Montico B, Lum B, Colizzi F, Giurato G, Salvati A, et al. Antibody dependent cellular cytotoxicity-inducing anti-EGFR antibodies as effective therapeutic option for cutaneous melanoma resistant to BRAF inhibitors. Front Immunol. 2024;15:1336566. 10.3389/fimmu.2024.1336566. 38510242 10.3389/fimmu.2024.1336566 PMC10950948

[126] Kreß JKC, Jessen C, Marquardt A, Hufnagel A, Meierjohann S. Nrf2 enables EGFR signaling in melanoma cells. Int J Mol Sci. 2021. 10.3390/ijms22083803. 33916908 10.3390/ijms22083803 PMC8067606

[127] Simiczyjew A, Wądzyńska J, Kot M, Ziętek M, Matkowski R, Hoang MP, et al. Combinations of EGFR and MET inhibitors reduce proliferation and invasiveness of mucosal melanoma cells. J Cell Mol Med. 2023;27(19):2995–3008. 10.1111/jcmm.17935. 37679999 10.1111/jcmm.17935 PMC10538264

[128] Billing O, Holmgren Y, Nosek D, Hedman H, Hemmingsson O. LRIG1 is a conserved EGFR regulator involved in melanoma development, survival and treatment resistance. Oncogene. 2021;40(21):3707–18. 10.1038/s41388-021-01808-3. 33947959 10.1038/s41388-021-01808-3 PMC8154585

[129] Wang M, Cao Y, Ren C, Wang K, Wang Y, Wu X, et al. Ptrf confers melanoma-acquired drug resistance through the upregulation of EGFR. Cell Prolif. 2025. 10.1111/cpr.70086. 40745979 10.1111/cpr.70086 PMC12877943

[130] Bai W, Yan C, Yang Y, Sang L, Hao Q, Yao X, et al. EGF/EGFR-YAP1/TEAD2 signaling upregulates STIM1 in vemurafenib resistant melanoma cells. FEBS J. 2024;291(22):4969–83. 10.1111/febs.17272. 39298503 10.1111/febs.17272

[131] Ji N, Li H, Zhang Y, Li Y, Wang P, Chen X, et al. Lansoprazole (LPZ) reverses multidrug resistance (MDR) in cancer through impeding ATP-binding cassette (ABC) transporter-mediated chemotherapeutic drug efflux and lysosomal sequestration. Drug Resist Updat. 2024;76:101100. 10.1016/j.drup.2024.101100. 38885537 10.1016/j.drup.2024.101100

[132] To KKW, Huang Z, Zhang H, Ashby CR, Fu L. Utilizing non-coding RNA-mediated regulation of ATP binding cassette (ABC) transporters to overcome multidrug resistance to cancer chemotherapy. Drug Resist Updat. 2024;73:101058. 10.1016/j.drup.2024.101058. 38277757 10.1016/j.drup.2024.101058

[133] Bharathiraja P, Yadav P, Sajid A, Ambudkar SV, Prasad NR. Natural medicinal compounds target signal transduction pathways to overcome ABC drug efflux transporter-mediated multidrug resistance in cancer. Drug Resist Updat. 2023;71:101004. 10.1016/j.drup.2023.101004. 37660590 10.1016/j.drup.2023.101004 PMC10840887

[134] Nobili S, Lapucci A, Landini I, Coronnello M, Roviello G, Mini E. Role of ATP-binding cassette transporters in cancer initiation and progression. Semin Cancer Biol. 2020;60:72–95. 10.1016/j.semcancer.2019.08.006. 31412294 10.1016/j.semcancer.2019.08.006

[135] Wagner M, Blum D, Raschka SL, Nentwig LM, Gertzen CGW, Chen M, et al. A new twist in ABC transporter mediated multidrug resistance - Pdr5 is a drug/proton co-transporter. J Mol Biol. 2022;434(14):167669. 10.1016/j.jmb.2022.167669. 35671830 10.1016/j.jmb.2022.167669

[136] Poustforoosh A, Moosavi F. Evaluation of the FDA-approved kinase inhibitors to uncover the potential repurposing candidates targeting ABC transporters in multidrug-resistant cancer cells: an in silico approach. J Biomol Struct Dyn. 2024;42(24):13650–62. 10.1080/07391102.2023.2277848. 37942620 10.1080/07391102.2023.2277848

[137] Wu CP, Hsiao SH, Wu YS. Perspectives on drug repurposing to overcome cancer multidrug resistance mediated by ABCB1 and ABCG2. Drug Resist Updat. 2023;71:101011. 10.1016/j.drup.2023.101011. 37865067 10.1016/j.drup.2023.101011

[138] Li D, Liu S, Ge Y, Li H, Ke X, Cao D, et al. Eupatilin attenuates vemurafenib resistance through inhibition of ABCB1 in melanoma. J Dermatol Sci. 2025. 10.1016/j.jdermsci.2025.06.003. 40628591 10.1016/j.jdermsci.2025.06.003

[139] Zhang P, Kuil LE, Buil LCM, Freriks S, Beijnen JH, van Tellingen O, et al. Acquired and intrinsic resistance to vemurafenib in BRAF V600E -driven melanoma brain metastases. FEBS Open Bio. 2024;14(1):96–111. 10.1002/2211-5463.13730. 37953496 10.1002/2211-5463.13730 PMC10761933

[140] Kim DY, Yun H, You JE, Koh DI, Ryu YS, Jin DH. Identification of UBE3C as an E3 ubiquitin ligase for mutant BRAF. Life Sci. 2025;378:123827. 10.1016/j.lfs.2025.123827. 40602747 10.1016/j.lfs.2025.123827

[141] Pizzichetta MA, Polesel J, Sini MC, Manca A, Simi S, Paliogiannis P, et al. Clinical, histopathological, dermoscopic features, and BRAF, NRAS, and cell cycle genes’ mutation status in cutaneous melanoma. Cancers (Basel). 2025. 10.3390/cancers17162688. 40867317 10.3390/cancers17162688 PMC12384477

[142] Gureghian V, Herbst H, Kozar I, Mihajlovic K, Malod-Dognin N, Ceddia G, et al. A multi-omics integrative approach unravels novel genes and pathways associated with senescence escape after targeted therapy in NRAS mutant melanoma. Cancer Gene Ther. 2023;30(10):1330–45. 10.1038/s41417-023-00640-z. 37420093 10.1038/s41417-023-00640-z PMC10581906

[143] Noma IHY, Carvalho LADC, Camarena DEM, Silva RO, Moraes Junior MO, de Souza ST, et al. Peroxiredoxin-2 represses NRAS-mutated melanoma cells invasion by modulating EMT markers. Biomed Pharmacother Biomed Pharmacother. 2024;177:116953. 10.1016/j.biopha.2024.116953. 38955087 10.1016/j.biopha.2024.116953

[144] Jour G, Illa-Bochaca I, Ibrahim M, Donnelly D, Zhu K, Miera EV, et al. Genomic and Transcriptomic Analyses of NF1-Mutant Melanoma Identify Potential Targeted Approach for Treatment. J Invest Dermatol. 2023;143(3):444-455.e448. 10.1016/j.jid.2022.07.022. 35988589 10.1016/j.jid.2022.07.022

[145] Vasudevan HN, Delley C, Chen WC, Mirchia K, Pan S, Shukla P, et al. Molecular features of resected melanoma brain metastases, clinical outcomes, and responses to immunotherapy. JAMA Netw Open. 2023;6(8):e2329186. 10.1001/jamanetworkopen.2023.29186. 37589977 10.1001/jamanetworkopen.2023.29186 PMC10436135

[146] Adam C, Fusi L, Weiss N, Goller SG, Meder K, Frings VG, et al. Efficient Suppression of NRAS-Driven Melanoma by Co-Inhibition of ERK1/2 and ERK5 MAPK Pathways. J Invest Dermatol. 2020;140(12):2455-2465.e2410. 10.1016/j.jid.2020.03.972. 32376279 10.1016/j.jid.2020.03.972

[147] Nguyen MQ, Teh JLF, Purwin TJ, Chervoneva I, Davies MA, Nathanson KL, et al. Targeting PHGDH Upregulation Reduces Glutathione Levels and Resensitizes Resistant NRAS-Mutant Melanoma to MAPK Kinase Inhibition. J Invest Dermatol. 2020;140(11):2242-2252.e2247. 10.1016/j.jid.2020.02.047. 32389536 10.1016/j.jid.2020.02.047 PMC7606255

[148] Tóvári J, Vári-Mező D, Surguta SE, Ladányi A, Kigyós A, Cserepes M. Evolving acquired vemurafenib resistance in a BRAF V600E mutant melanoma PDTX model to reveal new potential targets. Cells. 2023. 10.3390/cells12141919. 37508582 10.3390/cells12141919 PMC10377807

[149] Sabbah M, Krayem M, Najem A, Sales F, Miller W, Del Rincon S, et al. Dasatinib stimulates its own mechanism of resistance by activating a CRTC3/MITF/Bcl-2 pathway in melanoma with mutant or amplified c-Kit. Mol Cancer Res. 2021;19(7):1221–33. 10.1158/1541-7786.Mcr-20-1040. 33741716 10.1158/1541-7786.MCR-20-1040

[150] Maji L, Teli G, Raghavendra NM, Sengupta S, Pal R, Ghara A, et al. An updated literature on BRAF inhibitors (2018–2023). Mol Divers. 2024;28(4):2689–730. 10.1007/s11030-023-10699-3. 37470921 10.1007/s11030-023-10699-3

[151] Lamberti J, Memoli D, Montico B, Silvestro F, Guerrieri R, Colizzi F, et al. Non-coding RNA profiling in BRAF V600E -mutant cutaneous melanoma before and after Spry1 depletion. Sci Data. 2025;12(1):1538. 10.1038/s41597-025-05807-x. 40897749 10.1038/s41597-025-05807-x PMC12405587

[152] Galus Ł, Kolenda T, Michalak M, Mackiewicz J. Diagnostic and prognostic role of long non-coding RNAs (lncRNAs) in metastatic melanoma patients with BRAF gene mutation receiving BRAF and MEK inhibitors. Heliyon. 2024;10(7):e29071. 10.1016/j.heliyon.2024.e29071. 38601651 10.1016/j.heliyon.2024.e29071 PMC11004874

[153] Hung KL, Luebeck J, Dehkordi SR, Colón CI, Li R, Wong IT, et al. Targeted profiling of human extrachromosomal DNA by CRISPR-CATCH. Nat Genet. 2022;54(11):1746–54. 10.1038/s41588-022-01190-0. 36253572 10.1038/s41588-022-01190-0 PMC9649439

[154] Salvadores M, Fuster-Tormo F, Supek F. Matching cell lines with cancer type and subtype of origin via mutational, epigenomic, and transcriptomic patterns. Sci Adv. 2020. 10.1126/sciadv.aba1862. 32937430 10.1126/sciadv.aba1862 PMC7458440

[155] Ohnmacht AJ, Rajamani A, Avar G, Kutkaite G, Gonçalves E, Saur D, et al. The pharmacoepigenomic landscape of cancer cell lines reveals the epigenetic component of drug sensitivity. Commun Biol. 2023;6(1):825. 10.1038/s42003-023-05198-y. 37558831 10.1038/s42003-023-05198-y PMC10412573

[156] Gassenmaier M, Rentschler M, Fehrenbacher B, Eigentler TK, Ikenberg K, Kosnopfel C, et al. Expression of DNA methyltransferase 1 is a hallmark of melanoma, correlating with proliferation and response to B-Raf and mitogen-activated protein kinase inhibition in melanocytic tumors. Am J Pathol. 2020;190(10):2155–64. 10.1016/j.ajpath.2020.07.002. 32679231 10.1016/j.ajpath.2020.07.002

[157] De Beck L, Awad RM, Basso V, Casares N, De Ridder K, De Vlaeminck Y, et al. Inhibiting histone and DNA methylation improves cancer vaccination in an experimental model of melanoma. Front Immunol. 2022;13:799636. 10.3389/fimmu.2022.799636. 35634329 10.3389/fimmu.2022.799636 PMC9134079

[158] Jung H, Kim HS, Kim JY, Sun JM, Ahn JS, Ahn MJ, et al. DNA methylation loss promotes immune evasion of tumours with high mutation and copy number load. Nat Commun. 2019;10(1):4278. 10.1038/s41467-019-12159-9. 31537801 10.1038/s41467-019-12159-9 PMC6753140

[159] Falahat R, Berglund A, Putney RM, Perez-Villarroel P, Aoyama S, Pilon-Thomas S, et al. Epigenetic reprogramming of tumor cell-intrinsic STING function sculpts antigenicity and T cell recognition of melanoma. Proc Natl Acad Sci U S A. 2021. 10.1073/pnas.2013598118. 33827917 10.1073/pnas.2013598118 PMC8053941

[160] Fröhlich A, Loick S, Bawden EG, Fietz S, Dietrich J, Diekmann E, et al. Comprehensive analysis of tumor necrosis factor receptor TNFRSF9 (4–1BB) DNA methylation with regard to molecular and clinicopathological features, immune infiltrates, and response prediction to immunotherapy in melanoma. EBioMedicine. 2020;52:102647. 10.1016/j.ebiom.2020.102647. 32028068 10.1016/j.ebiom.2020.102647 PMC6997575

[161] Afsar S, Syed RU, Khojali WMA, Masood N, Osman ME, Jyothi JS, et al. Non-coding RNAs in BRAF-mutant melanoma: targets, indicators, and therapeutic potential. Naunyn Schmiedebergs Arch Pharmacol. 2025;398(1):297–317. 10.1007/s00210-024-03366-3. 39167168 10.1007/s00210-024-03366-3

[162] Díaz-Martínez M, Benito-Jardón L, Alonso L, Koetz-Ploch L, Hernando E, Teixidó J. miR-204-5p and miR-211-5p contribute to BRAF inhibitor resistance in melanoma. Cancer Res. 2018;78(4):1017–30. 10.1158/0008-5472.Can-17-1318. 29229605 10.1158/0008-5472.CAN-17-1318 PMC5815895

[163] Grzywa TM, Klicka K, Paskal W, Dudkiewicz J, Wejman J, Pyzlak M, et al. miR-410-3p is induced by vemurafenib via ER stress and contributes to resistance to BRAF inhibitor in melanoma. PLoS ONE. 2020;15(6):e0234707. 10.1371/journal.pone.0234707. 32555626 10.1371/journal.pone.0234707 PMC7299409

[164] Fattore L, Ruggiero CF, Pisanu ME, Liguoro D, Cerri A, Costantini S, et al. Reprogramming miRNAs global expression orchestrates development of drug resistance in BRAF mutated melanoma. Cell Death Differ. 2019;26(7):1267–82. 10.1038/s41418-018-0205-5. 30254376 10.1038/s41418-018-0205-5 PMC6748102

[165] Bezrookove V, Khan I, Bhattacharjee A, Fan J, Jones R, Sharma A, et al. miR-876-3p is a tumor suppressor on 9p21 that is inactivated in melanoma and targets ERK. J Transl Med. 2024;22(1):758. 10.1186/s12967-024-05527-7. 39138582 10.1186/s12967-024-05527-7 PMC11321151

[166] Barbato A, Iuliano A, Volpe M, D’Alterio R, Brillante S, Massa F, et al. Integrated genomics identifies miR-181/TFAM pathway as a critical driver of drug resistance in melanoma. Int J Mol Sci. 2021. 10.3390/ijms22041801. 33670365 10.3390/ijms22041801 PMC7918089

[167] Vitiello M, Mercatanti A, Podda MS, Baldanzi C, Prantera A, Sarti S, et al. A network of microRNAs and mRNAs involved in melanosome maturation and trafficking defines the lower response of pigmentable melanoma cells to targeted therapy. Cancers (Basel). 2023. 10.3390/cancers15030894. 36765859 10.3390/cancers15030894 PMC9913661

[168] Luan W, Lu X, Peng H, Shen X, Rao M, Ruan H. Exosomal miR-19a derived from melanoma cell promotes the vemurafenib resistance of malignant melanoma through directly targeting LRIG1 to reactivate AKT and MAPK pathway. Pathol Res Pract. 2024;260:155410. 10.1016/j.prp.2024.155410. 38955119 10.1016/j.prp.2024.155410

[169] Keser M, Atmaca H. Let-7a microRNA modulates caspase-3-dependent apoptosis in melanoma cells treated with dabrafenib and trametinib combination. Ir J Med Sci. 2025;194(3):797–805. 10.1007/s11845-025-03923-6. 40063190 10.1007/s11845-025-03923-6 PMC12276103

[170] Lee B, Sahoo A, Sawada J, Marchica J, Sahoo S, Layng FIAL, et al. MicroRNA-211 modulates the DUSP6-ERK5 signaling axis to promote BRAF V600E -driven melanoma growth in vivo and BRAF/MEK inhibitor resistance. J Invest Dermatol. 2021;141(2):385–94. 10.1016/j.jid.2020.06.038. 32888955 10.1016/j.jid.2020.06.038 PMC9004592

[171] Ostrowski SM, Fisher DE. The melanocyte lineage factor miR-211 promotes BRAF V600E inhibitor resistance. J Invest Dermatol. 2021;141(2):250–2. 10.1016/j.jid.2020.07.010. 33504438 10.1016/j.jid.2020.07.010 PMC7850168

[172] Vitiello M, D’Aurizio R, Poliseno L. Biological role of miR-204 and miR-211 in melanoma. Oncoscience. 2018;5(7–8):248–51. 10.18632/oncoscience.443. 30234146 10.18632/oncoscience.443 PMC6142896

[173] Castaldo V, Minopoli M, Di Modugno F, Sacconi A, Liguoro D, Frigerio R, et al. Upregulated expression of miR-4443 and miR-4488 in drug resistant melanomas promotes migratory and invasive phenotypes through downregulation of intermediate filament nestin. J Exp Clin Cancer Res. 2023;42(1):317. 10.1186/s13046-023-02878-9. 38008717 10.1186/s13046-023-02878-9 PMC10680267

[174] Han S, Yan Y, Ren Y, Hu Y, Wang Y, Chen L, et al. LncRNA SAMMSON mediates adaptive resistance to RAF inhibition in BRAF-mutant melanoma cells. Cancer Res. 2021;81(11):2918–29. 10.1158/0008-5472.Can-20-3145. 34087780 10.1158/0008-5472.CAN-20-3145

[175] Leucci E, Vendramin R, Spinazzi M, Laurette P, Fiers M, Wouters J, et al. Melanoma addiction to the long non-coding RNA SAMMSON. Nature. 2016;531(7595):518–22. 10.1038/nature17161. 27008969 10.1038/nature17161

[176] Cardoso C, Serafim RB, Kawakami A, Gonçalves Pereira C, Roszik J, Valente V, et al. The lncRNA RMEL3 protects immortalized cells from serum withdrawal-induced growth arrest and promotes melanoma cell proliferation and tumor growth. Pigment Cell Melanoma Res. 2019;32(2):303–14. 10.1111/pcmr.12751. 30457212 10.1111/pcmr.12751 PMC6613776

[177] Wang X, Cheng Q. Suppression of exosomal hsa_circ_0001005 eliminates the vemurafenib resistance of melanoma. J Cancer Res Clin Oncol. 2023;149(9):5921–36. 10.1007/s00432-022-04434-y. 36598578 10.1007/s00432-022-04434-y PMC11798329

[178] Wang Y, Gong J, Ding X, Luo S. CircRTTN upregulates EPHA2 to aggravate the malignant process of melanoma via sponging miR-890. Histol Histopathol. 2024;39(2):211–24. 10.14670/hh-18-622. 37158505 10.14670/HH-18-622

[179] Naik H, Choudhary R, Konkimalla VB. In silico analysis of novel circRNA-miRNA-mRNA axis in BRAF V600E melanoma: implications for primary to metastasis transformation and TIME modulation. J Gene Med. 2025;27(6):e70023. 10.1002/jgm.70023. 40436810 10.1002/jgm.70023

[180] Yang Y, Li J, Wei C, Wang L, Gao Z, Shen K, et al. Circular RNA circFCHO2(hsa_circ_0002490) promotes the proliferation of melanoma by directly binding to DND1. Cell Biol Toxicol. 2024;40(1):9. 10.1007/s10565-024-09851-y. 38311675 10.1007/s10565-024-09851-y PMC10838848

[181] Siena ÁDD, Barros II, Storti CB, de Biagi Júnior CAO, da Costa Carvalho LA, Maria-Engler SS, et al. Upregulation of the novel lncRNA U731166 is associated with migration, invasion and vemurafenib resistance in melanoma. J Cell Mol Med. 2022;26(3):671–83. 10.1111/jcmm.16987. 35040264 10.1111/jcmm.16987 PMC8817119

[182] You H, Li Q, Kong D, Liu X, Kong F, Zheng K, et al. The interaction of canonical Wnt/β-catenin signaling with protein lysine acetylation. Cell Mol Biol Lett. 2022;27(1):7. 10.1186/s11658-021-00305-5. 35033019 10.1186/s11658-021-00305-5 PMC8903542

[183] Yuan G, Flores NM, Hausmann S, Lofgren SM, Kharchenko V, Angulo-Ibanez M, et al. Elevated NSD3 histone methylation activity drives squamous cell lung cancer. Nature. 2021;590(7846):504–8. 10.1038/s41586-020-03170-y. 33536620 10.1038/s41586-020-03170-y PMC7895461

[184] Wang H, Yang W, Qin Q, Yang X, Yang Y, Liu H, et al. E3 ubiquitin ligase MAGI3 degrades c-Myc and acts as a predictor for chemotherapy response in colorectal cancer. Mol Cancer. 2022;21(1):151. 10.1186/s12943-022-01622-9. 35864508 10.1186/s12943-022-01622-9 PMC9306183

[185] Zhou J, Chai X, Zhu Y, Huang Z, Lin T, Hu Z, et al. A methyl-to-acetyl switch in H3K27 drives metabolic reprogramming and resistance to BRAF V600E inhibition in melanoma. Neoplasia (New York, NY). 2025;68:101223. 10.1016/j.neo.2025.101223. 10.1016/j.neo.2025.101223 PMC12446969 40850308

[186] Wu T, Li C, Zhou C, Niu X, Li G, Zhou Y, et al. Inhibition of USP14 enhances anti-tumor effect in vemurafenib-resistant melanoma by regulation of Skp2. Cell Biol Toxicol. 2023;39(5):2381–99. 10.1007/s10565-022-09729-x. 35648318 10.1007/s10565-022-09729-x

[187] Shattuck-Brandt RL, Chen SC, Murray E, Johnson CA, Crandall H, O’Neal JF, et al. Metastatic melanoma patient-derived xenografts respond to MDM2 inhibition as a single agent or in combination with BRAF/MEK inhibition. Clin Cancer Res. 2020;26(14):3803–18. 10.1158/1078-0432.Ccr-19-1895. 32234759 10.1158/1078-0432.CCR-19-1895 PMC7367743

[188] Ma ZR, Xiong QW, Cai SZ, Ding LT, Yin CH, Xia HL, et al. USP18 enhances the resistance of BRAF-mutated melanoma cells to vemurafenib by stabilizing cGAS expression to induce cell autophagy. Int Immunopharmacol. 2023;122:110617. 10.1016/j.intimp.2023.110617. 37478666 10.1016/j.intimp.2023.110617

[189] Grigore F, Yang H, Hanson ND, VanBrocklin MW, Sarver AL, Robinson JP. BRAF inhibition in melanoma is associated with the dysregulation of histone methylation and histone methyltransferases. Neoplasia (New York, NY). 2020;22(9):376–89. 10.1016/j.neo.2020.06.006. 10.1016/j.neo.2020.06.006 PMC7338995 32629178

[190] Giblin W, Bringman-Rodenbarger L, Guo AH, Kumar S, Monovich AC, Mostafa AM, et al. The deacylase SIRT5 supports melanoma viability by influencing chromatin dynamics. J Clin Invest. 2021;131(12). 10.1172/jci138926. 10.1172/JCI138926 PMC8203465 33945506

[191] Huang X, Yi P, Gou W, Zhang R, Wu C, Liu L, et al. Neddylation signaling inactivation by tetracaine hydrochloride suppresses cell proliferation and alleviates vemurafenib-resistance of melanoma. Cell Biol Toxicol. 2024;40(1):81. 10.1007/s10565-024-09916-y. 39297891 10.1007/s10565-024-09916-y PMC11413085

[192] Li A, Gong Z, Long Y, Li Y, Liu C, Lu X, et al. Lactylation of LSD1 is an acquired epigenetic vulnerability of BRAFi/MEKi-resistant melanoma. Dev Cell. 2025;60(14):1974-1990.e1911. 10.1016/j.devcel.2025.02.016. 40132584 10.1016/j.devcel.2025.02.016

[193] Sun Y, Yu H, Zhou Y, Bao J, Qian X. EGFR influences the resistance to targeted therapy in BRAF V600E melanomas by regulating the ferroptosis process. Arch Dermatol Res. 2025;317(1):514. 10.1007/s00403-025-03895-8. 40024937 10.1007/s00403-025-03895-8 PMC11872748

[194] Li G, Zhou C, Wang L, Zheng Y, Zhou B, Li G, et al. Mitocur-1 induces ferroptosis to reverse vemurafenib resistance in melanoma through inhibition of USP14. Pigment Cell Melanoma Res. 2024;37(2):316–28. 10.1111/pcmr.13150. 37985430 10.1111/pcmr.13150

[195] Park BS, Jeon H, Kim Y, Kwon H, Choi GE, Chi SG, et al. Polyamine and EIF5A hypusination downstream of c-Myc confers targeted therapy resistance in BRAF mutant melanoma. Mol Cancer. 2024;23(1):136. 10.1186/s12943-024-02031-w. 38965534 10.1186/s12943-024-02031-w PMC11223307

[196] Rubinstein JC, Domanskyi S, Sheridan TB, Sanderson B, Park S, Kaster J, et al. Spatiotemporal profiling defines persistence and resistance dynamics during targeted treatment of melanoma. Cancer Res. 2025;85(5):987–1002. 10.1158/0008-5472.Can-24-0690. 39700408 10.1158/0008-5472.CAN-24-0690 PMC11875961

[197] Vom Stein AF, Hallek M, Nguyen PH. Role of the tumor microenvironment in CLL pathogenesis. Semin Hematol. 2024;61(3):142–54. 10.1053/j.seminhematol.2023.12.004. 38220499 10.1053/j.seminhematol.2023.12.004

[198] Dey P, Li J, Zhang J, Chaurasiya S, Strom A, Wang H, et al. Oncogenic KRAS-driven metabolic reprogramming in pancreatic cancer cells utilizes cytokines from the tumor microenvironment. Cancer Discov. 2020;10(4):608–25. 10.1158/2159-8290.Cd-19-0297. 32046984 10.1158/2159-8290.CD-19-0297 PMC7125035

[199] Li H, Zhang Y, Xu Y, Huang Z, Cheng G, Xie M, et al. Tumor immune microenvironment and immunotherapy efficacy in BRAF mutation non-small-cell lung cancer. Cell Death Dis. 2022;13(12):1064. 10.1038/s41419-022-05510-4. 36543792 10.1038/s41419-022-05510-4 PMC9772302

[200] Wang M, Zadeh S, Pizzolla A, Thia K, Gyorki DE, McArthur GA, et al. Characterization of the treatment-naive immune microenvironment in melanoma with BRAF mutation. J Immunother Cancer. 2022. 10.1136/jitc-2021-004095. 35383113 10.1136/jitc-2021-004095 PMC8984014

[201] Florent L, Saby C, Courageot MP, Terryn C, Van Gulick L, Vanmansart J, et al. Age-associated changes in type I collagen promote the invasion of BRAF V600E mutated melanoma cells and their resistance to targeted therapies within three-dimensional matrix models. Biomed Pharmacother. 2025;190:118351. 10.1016/j.biopha.2025.118351. 40684498 10.1016/j.biopha.2025.118351

[202] Shin DS, Schroeder ME, Anseth KS. Impact of Collagen Triple Helix Structure on Melanoma Cell Invadopodia Formation and Matrix Degradation upon BRAF Inhibitor Treatment. Adv Healthc Mater. 2021. 10.1002/adhm.202101592. 34783464 10.1002/adhm.202101592 PMC8986579

[203] Vasilevska J, Cheng PF, Lehmann J, Ramelyte E, Gómez JM, Dimitriou F, et al. Monitoring melanoma patients on treatment reveals a distinct macrophage population driving targeted therapy resistance. Cell reports Medicine. 2024;5(7):101611. 10.1016/j.xcrm.2024.101611. 38942020 10.1016/j.xcrm.2024.101611 PMC11293307

[204] Chhabra Y, Fane ME, Pramod S, Hüser L, Zabransky DJ, Wang V, et al. Sex-dependent effects in the aged melanoma tumor microenvironment influence invasion and resistance to targeted therapy. Cell. 2024;187(21):6016-6034.e6025. 10.1016/j.cell.2024.08.013. 39243764 10.1016/j.cell.2024.08.013 PMC11580838

[205] Alicea GM, Rebecca VW, Goldman AR, Fane ME, Douglass SM, Behera R, et al. Changes in aged fibroblast lipid metabolism induce age-dependent melanoma cell resistance to targeted therapy via the fatty acid transporter FATP2. Cancer Discov. 2020;10(9):1282–95. 10.1158/2159-8290.Cd-20-0329. 32499221 10.1158/2159-8290.CD-20-0329 PMC7483379

[206] Piotrowska A, Zaucha R, Król O, Żmijewski MA. Vitamin D Modulates the Response of Patient-Derived Metastatic Melanoma Cells to Anticancer Drugs. Int J Mol Sci. 2023. 10.3390/ijms24098037. 37175742 10.3390/ijms24098037 PMC10178305

[207] Morales D, Vigneron P, Ferreira I, Hamitou W, Magnano M, Mahenthiran L, et al. Fibroblasts Influence Metastatic Melanoma Cell Sensitivity to Combined BRAF and MEK Inhibition. Cancers (Basel). 2021. 10.3390/cancers13194761. 34638245 10.3390/cancers13194761 PMC8507536

[208] Benedicto A, Hernandez-Unzueta I, Sanz E, Márquez J. Ocoxin Increases the Antitumor Effect of BRAF Inhibition and Reduces Cancer Associated Fibroblast-Mediated Chemoresistance and Protumoral Activity in Metastatic Melanoma. Nutrients. 2021. 10.3390/nu13020686. 33669949 10.3390/nu13020686 PMC7924874

[209] Liu T, Zhou L, Xiao Y, Andl T, Zhang Y. BRAF inhibitors reprogram cancer-associated fibroblasts to drive matrix remodeling and therapeutic escape in melanoma. Cancer Res. 2022;82(3):419–32. 10.1158/0008-5472.Can-21-0614. 35064015 10.1158/0008-5472.CAN-21-0614

[210] Foda BM, Neubig RR. Role of Rho/MRTF in Aggressive Vemurafenib-Resistant Murine Melanomas and Immune Checkpoint Upregulation. Int J Mol Sci. 2023. 10.3390/ijms241813785. 37762086 10.3390/ijms241813785 PMC10531039

[211] Zhao K, Lu Y, Chen Y, Cheng J, Zhang W. Transcripts 202 and 205 of IL-6 confer resistance to Vemurafenib by reactivating the MAPK pathway in BRAF(V600E) mutant melanoma cells. Exp Cell Res. 2020;390(2):111942. 10.1016/j.yexcr.2020.111942. 32173467 10.1016/j.yexcr.2020.111942

[212] Giraulo C, Orlando L, Morretta E, Voli A, Plaitano P, Cicala C, et al. High levels of soluble CD73 unveil resistance to BRAF inhibitors in melanoma cells. Biomedi Pharmacother Biomed Pharmacother. 2024;177:117033. 10.1016/j.biopha.2024.117033. 10.1016/j.biopha.2024.117033 38941889

[213] Tabolacci C, Cordella M, Mariotti S, Rossi S, Senatore C, Lintas C, et al. Melanoma Cell Resistance to Vemurafenib Modifies Inter-Cellular Communication Signals. Biomedicines. 2021;9(1). 10.3390/biomedicines9010079. 10.3390/biomedicines9010079 PMC7830125 33467521

[214] Barceló C, Sisó P, de la Rosa I, Megino-Luque C, Navaridas R, Maiques O, et al. M-CSF as a therapeutic target in BRAF V600E melanoma resistant to BRAF inhibitors. Br J Cancer. 2022;127(6):1142–52. 10.1038/s41416-022-01886-4. 35725813 10.1038/s41416-022-01886-4 PMC9470708

[215] Erkes DA, Rosenbaum SR, Field CO, Chervoneva I, Villanueva J, Aplin AE. PLX3397 inhibits the accumulation of intra-tumoral macrophages and improves bromodomain and extra-terminal inhibitor efficacy in melanoma. Pigment Cell Melanoma Res. 2020;33(2):372–7. 10.1111/pcmr.12845. 31696640 10.1111/pcmr.12845 PMC7028511

[216] Kilmister EJ, Tan ST. Cancer stem cells and the renin-angiotensin system in the tumor microenvironment of melanoma: implications on current therapies. Int J Mol Sci. 2025;26(3). 10.3390/ijms26031389. 10.3390/ijms26031389 PMC11818896 39941158

[217] Ghezzi B, Fiorilla I, Carreira Á, Recco F, Sorci L, Avalle L, et al. NAMPT and NNMT released via extracellular vesicles and as soluble mediators are distinguished traits of BRAF inhibitor resistance of melanoma cells impacting on the tumor microenvironment. Cell Commun Signal. 2025;23(1):348. 10.1186/s12964-025-02361-2. 40691620 10.1186/s12964-025-02361-2 PMC12278642

[218] Bi Y, Liu J, Qin S, Ji F, Zhou C, Yang H, et al. CDKL3 shapes immunosuppressive tumor microenvironment and initiates autophagy in esophageal cancer. Front Immunol. 2024;15:1295011. 10.3389/fimmu.2024.1295011. 38562942 10.3389/fimmu.2024.1295011 PMC10982402

[219] Ferraresi A, Girone C, Esposito A, Vidoni C, Vallino L, Secomandi E, et al. How autophagy shapes the tumor microenvironment in ovarian cancer. Front Oncol. 2020;10:599915. 10.3389/fonc.2020.599915. 33364196 10.3389/fonc.2020.599915 PMC7753622

[220] Frias A, Di Leo L, Antoranz A, Nazerai L, Carretta M, Bodemeyer V, et al. Ambra1 modulates the tumor immune microenvironment and response to PD-1 blockade in melanoma. J Immunother Cancer. 2023;11(3). 10.1136/jitc-2022-006389. 10.1136/jitc-2022-006389 PMC9990656 36868570

[221] Huang PY, Liang SY, Xiang Y, Li MR, Wang MR, Liu LH. Endoplasmic reticulum-targeting self-assembly nanosheets promote autophagy and regulate immunosuppressive tumor microenvironment for efficient photodynamic immunotherapy. Small. 2024;20(25):e2311056. 10.1002/smll.202311056. 38377262 10.1002/smll.202311056

[222] Hou W, Xiao C, Zhou R, Yao X, Chen Q, Xu T, et al. Inhibiting autophagy selectively prunes dysfunctional tumor vessels and optimizes the tumor immune microenvironment. Theranostics. 2025;15(1):258–76. 10.7150/thno.98285. 39744218 10.7150/thno.98285 PMC11667230

[223] Yan J, Shan C, Zhang Z, Li F, Sun Y, Wang Q, et al. Autophagy-induced intracellular signaling fractional nano-drug system for synergistic anti-tumor therapy. J Colloid Interface Sci. 2023;645:986–96. 10.1016/j.jcis.2023.05.031. 37179196 10.1016/j.jcis.2023.05.031

[224] Young MJ, Wang SA, Chen YC, Liu CY, Hsu KC, Tang SW, et al. USP24-i-101 targeting of USP24 activates autophagy to inhibit drug resistance acquired during cancer therapy. Cell Death Differ. 2024;31(5):574–91. 10.1038/s41418-024-01277-7. 38491202 10.1038/s41418-024-01277-7 PMC11093971

[225] Li M, Zhao D, Yan J, Fu X, Li F, Liu G, et al. A redox-triggered autophagy-induced nanoplatform with PD-L1 inhibition for enhancing combined chemo-immunotherapy. ACS Nano. 2024;18(20):12870–84. 10.1021/acsnano.4c00227. 38727063 10.1021/acsnano.4c00227

[226] Lei T, Cai X, Zhang H, Wu X, Cao Z, Li W, et al. Bmal1 upregulates ATG5 expression to promote autophagy in skin cutaneous melanoma. Cell Signal. 2024;124:111439. 10.1016/j.cellsig.2024.111439. 39343115 10.1016/j.cellsig.2024.111439

[227] Elshazly AM, Gewirtz DA. The Cytoprotective Role of Autophagy in Response to BRAF-Targeted Therapies. Int J Mol Sci. 2023;24(19). 10.3390/ijms241914774. 10.3390/ijms241914774 PMC10572960 37834222

[228] Cristofani R, Piccolella M, Montagnani Marelli M, Tedesco B, Poletti A, Moretti RM. HSPB8 counteracts tumor activity of BRAF- and NRAS-mutant melanoma cells by modulation of RAS-prenylation and autophagy. Cell Death Dis. 2022;13(11):973. 10.1038/s41419-022-05365-9. 36400750 10.1038/s41419-022-05365-9 PMC9674643

[229] L’Hôte V, Courbeyrette R, Pinna G, Cintrat JC, Le Pavec G, Delaunay-Moisan A, et al. Ouabain and chloroquine trigger senolysis of BRAF-V600E-induced senescent cells by targeting autophagy. Aging Cell. 2021;20(9):e13447. 10.1111/acel.13447. 34355491 10.1111/acel.13447 PMC8564827

[230] Pérez CN, Falcón CR, Mons JD, Orlandi FC, Sangiacomo M, Fernandez-Muñoz JM, et al. Melanoma cells with acquired resistance to vemurafenib have decreased autophagic flux and display enhanced ability to transfer resistance. Biochimica et biophysica acta. Mol Basis Dis. 2023;1869(7):166801. 10.1016/j.bbadis.2023.166801. 10.1016/j.bbadis.2023.166801 37419396

[231] Verykiou S, Alexander M, Edwards N, Plummer R, Chaudhry B, Lovat PE, et al. Harnessing autophagy to overcome mitogen-activated protein kinase kinase inhibitor-induced resistance in metastatic melanoma. Br J Dermatol. 2019;180(2):346–56. 10.1111/bjd.17333. 30339727 10.1111/bjd.17333 PMC7816093

[232] Li Y, Feng Y, and Chen D. Interfering nuclear protein laminb1 induces DNA damage and reduces vemurafenib resistance in melanoma cells in vitro. Cancers (Basel). 2024;16(23). 10.3390/cancers16234060. 10.3390/cancers16234060 PMC11639818 39682248

[233] Martin S, Dudek-Peric AM, Garg AD, Roose H, Demirsoy S, Van Eygen S, et al. An autophagy-driven pathway of ATP secretion supports the aggressive phenotype of BRAF V600E inhibitor-resistant metastatic melanoma cells. Autophagy. 2017;13(9):1512–27. 10.1080/15548627.2017.1332550. 28722539 10.1080/15548627.2017.1332550 PMC5612289

[234] Liu-Smith F, Lin J. Unsupervised Analysis Reveals the Involvement of Key Immune Response Genes and the Matrisome in Resistance to BRAF and MEK Inhibitors in Melanoma. Cancers (Basel). 2024;16(13). 10.3390/cancers16132313. 10.3390/cancers16132313 PMC11240363 39001376

[235] Ying W. Phenomic studies on diseases: potential and challenges. Phenomics (Cham, Switzerland). 2023;3(3):285–99. 10.1007/s43657-022-00089-4. 36714223 10.1007/s43657-022-00089-4 PMC9867904

[236] Khaddour K, Buchbinder EI. Individualized neoantigen-directed melanoma therapy. Am J Clin Dermatol. 2025;26(2):225–35. 10.1007/s40257-025-00920-4. 39875711 10.1007/s40257-025-00920-4

[237] Mundi PS, Dela Cruz FS, Grunn A, Diolaiti D, Mauguen A, Rainey AR, et al. A transcriptome-based precision oncology platform for patient-therapy alignment in a diverse set of treatment-resistant malignancies. Cancer Discov. 2023;13(6):1386–407. 10.1158/2159-8290.Cd-22-1020. 37061969 10.1158/2159-8290.CD-22-1020 PMC10239356

[238] Church AJ, Corson LB, Kao PC, Imamovic-Tuco A, Reidy D, Doan D, et al. Molecular profiling identifies targeted therapy opportunities in pediatric solid cancer. Nat Med. 2022;28(8):1581–9. 10.1038/s41591-022-01856-6. 35739269 10.1038/s41591-022-01856-6 PMC10953704

[239] Dai X, Blancafort P, Wang P, Sgro A, Thompson EW, Ostrikov KK. Innovative Precision Gene-Editing Tools in Personalized Cancer Medicine. Adv Sci (Weinheim, Baden-Wurttemberg, Germany). 2020;7(12):1902552. 10.1002/advs.201902552. 10.1002/advs.201902552 PMC7312441 32596104

[240] Ciardiello F, Ciardiello D, Martini G, Napolitano S, Tabernero J, Cervantes A. Clinical management of metastatic colorectal cancer in the era of precision medicine. CA Cancer J Clin. 2022;72(4):372–401. 10.3322/caac.21728. 35472088 10.3322/caac.21728

[241] Jiang YZ, Liu Y, Xiao Y, Hu X, Jiang L, Zuo WJ, et al. Molecular subtyping and genomic profiling expand precision medicine in refractory metastatic triple-negative breast cancer: the FUTURE trial. Cell Res. 2021;31(2):178–86. 10.1038/s41422-020-0375-9. 32719455 10.1038/s41422-020-0375-9 PMC8027015

[242] Su D, Ruan Y, Shi Y, Cao D, Wu T, Dang T, et al. Molecular subtyping and genomic profiling expand precision medicine in KRAS wild-type pancreatic cancer. Cancer Sci. 2025;116(4):1094–106. 10.1111/cas.16456. 39833990 10.1111/cas.16456 PMC11967249

[243] Acanda De La Rocha AM, Berlow NE, Fader M, Coats ER, Saghira C, Espinal PS, et al. Feasibility of functional precision medicine for guiding treatment of relapsed or refractory pediatric cancers. Nat Med. 2024;30(4):990–1000. 10.1038/s41591-024-02848-4. 38605166 10.1038/s41591-024-02848-4 PMC11031400

[244] Salama AKS, Li S, Macrae ER, Park JI, Mitchell EP, Zwiebel JA, et al. Dabrafenib and trametinib in patients with tumors with BRAF V600E mutations: results of the NCI-MATCH trial subprotocol H. J Clin Oncol. 2020;38(33):3895–904. 10.1200/jco.20.00762. 32758030 10.1200/JCO.20.00762 PMC7676884

[245] Unger JM, Vaidya R, Albain KS, LeBlanc M, Minasian LM, Gotay CC, et al. Sex differences in risk of severe adverse events in patients receiving immunotherapy, targeted therapy, or chemotherapy in cancer clinical trials. J Clin Oncol. 2022;40(13):1474–86. 10.1200/jco.21.02377. 35119908 10.1200/JCO.21.02377 PMC9061143

[246] Mah SJ, Carter Ramirez DM, Schnarr K, Eiriksson LR, Gayowsky A, Seow H. Timing of palliative care, end-of-life quality indicators, and health resource utilization. JAMA Netw Open. 2024;7(10):e2440977. 10.1001/jamanetworkopen.2024.40977. 39466244 10.1001/jamanetworkopen.2024.40977 PMC11519754

[247] Melero I, de Miguel LM, de Velasco G, Garralda E, Martín-Liberal J, Joerger M, et al. Neutralizing GDF-15 can overcome anti-PD-1 and anti-PD-L1 resistance in solid tumours. Nature. 2025;637(8048):1218–27. 10.1038/s41586-024-08305-z. 39663448 10.1038/s41586-024-08305-z PMC11779642

[248] Larkin J, Chiarion-Sileni V, Gonzalez R, Grob JJ, Cowey CL, Lao CD, et al. Combined nivolumab and ipilimumab or monotherapy in untreated melanoma. N Engl J Med. 2015;373(1):23–34. 10.1056/NEJMoa1504030. 26027431 10.1056/NEJMoa1504030 PMC5698905

[249] Maio M, Lewis K, Demidov L, Mandalà M, Bondarenko I, Ascierto PA, et al. Adjuvant vemurafenib in resected, BRAF V600 mutation-positive melanoma (BRIM8): a randomised, double-blind, placebo-controlled, multicentre, phase 3 trial. Lancet Oncol. 2018;19(4):510–20. 10.1016/s1470-2045(18)30106-2. 29477665 10.1016/S1470-2045(18)30106-2

[250] Vergani E, Busico A, Dugo M, Devecchi A, Valeri B, Cossa M, et al. Genetic Layout of Melanoma Lesions Is Associated with BRAF/MEK-Targeted Therapy Resistance and Transcriptional Profiles. J Invest Dermatol. 2022;142(11):3030-3040.e3035. 10.1016/j.jid.2022.04.027. 35643181 10.1016/j.jid.2022.04.027

[251] Pérez-Guijarro E, Yang HH, Araya RE, El Meskini R, Michael HT, Vodnala SK, et al. Multimodel preclinical platform predicts clinical response of melanoma to immunotherapy. Nat Med. 2020;26(5):781–91. 10.1038/s41591-020-0818-3. 32284588 10.1038/s41591-020-0818-3 PMC8482620

[252] Floristán A, Morales L, Hanniford D, Martinez C, Castellano-Sanz E, Dolgalev I, et al. Functional analysis of RPS27 mutations and expression in melanoma. Pigment Cell Melanoma Res. 2020;33(3):466–79. 10.1111/pcmr.12841. 31663663 10.1111/pcmr.12841 PMC7180098

[253] Pipek O, Vizkeleti L, Doma V, Alpár D, Bödör C, Kárpáti S, et al. The driverless triple-wild-type (BRAF, RAS, KIT) cutaneous melanoma: whole genome sequencing discoveries. Cancers (Basel). 2023. 10.3390/cancers15061712. 36980598 10.3390/cancers15061712 PMC10046270

[254] Durand S, Tang Y, Pommier RM, Benboubker V, Grimont M, Boivin F, et al. ZEB1 controls a lineage-specific transcriptional program essential for melanoma cell state transitions. Oncogene. 2024;43(20):1489–505. 10.1038/s41388-024-03010-7. 38519642 10.1038/s41388-024-03010-7 PMC11090790

[255] Cannon AC, Budagyan K, Uribe-Alvarez C, Kurimchak AM, Araiza-Olivera D, Cai KQ, et al. Unique vulnerability of RAC1-mutant melanoma to combined inhibition of CDK9 and immune checkpoints. Oncogene. 2024;43(10):729–43. 10.1038/s41388-024-02947-z. 38243078 10.1038/s41388-024-02947-z PMC11157427

[256] Laguillaumie MO, Titah S, Guillemette A, Neve B, Leprêtre F, Ségard P, et al. Deciphering genetic and nongenetic factors underlying tumour dormancy: insights from multiomics analysis of two syngeneic MRD models of melanoma and leukemia. Biol Res. 2024;57(1):59. 10.1186/s40659-024-00540-y. 39223638 10.1186/s40659-024-00540-y PMC11370043

[257] Wongchenko MJ, McArthur GA, Dréno B, Larkin J, Ascierto PA, Sosman J, et al. Gene expression profiling in BRAF-mutated melanoma reveals patient subgroups with poor outcomes to vemurafenib that may be overcome by cobimetinib plus vemurafenib. Clin Cancer Res. 2017;23(17):5238–45. 10.1158/1078-0432.Ccr-17-0172. 28536307 10.1158/1078-0432.CCR-17-0172

[258] Alhassan SO, Abd Elmageed ZY, Errami Y, Wang G, Abi-Rached JA, Kandil E, et al. BRAF V600E-PROTAC versus inhibitors in melanoma cells: deep transcriptomic characterization. Clin Transl Med. 2025;15(3):e70251. 10.1002/ctm2.70251. 40045459 10.1002/ctm2.70251 PMC11882472

[259] Barceló C, Sisó P, Maiques O, García-Mulero S, Sanz-Pamplona R, Navaridas R, et al. T-type calcium channels as potential therapeutic targets in vemurafenib-resistant BRAF V600E melanoma. J Invest Dermatol. 2020;140(6):1253–65. 10.1016/j.jid.2019.11.014. 31877318 10.1016/j.jid.2019.11.014

[260] Cammann C, Kulla J, Wiebusch L, Walz C, Zhao F, Lowinus T, et al. Proteasome inhibition potentiates Kv1.3 potassium channel expression as therapeutic target in drug-sensitive and -resistant human melanoma cells. Biomed Pharmacother. 2023;168:115635. 10.1016/j.biopha.2023.115635. 37816303 10.1016/j.biopha.2023.115635

[261] DuBose E, Bevill SM, Mitchell DK, Sciaky N, Golitz BT, Dixon SAH, et al. Neratinib, a pan ERBB/HER inhibitor, restores sensitivity of PTEN-null, BRAFV600E melanoma to BRAF/MEK inhibition. Front Oncol. 2024;14:1191217. 10.3389/fonc.2024.1191217. 38854737 10.3389/fonc.2024.1191217 PMC11159048

[262] Ren M, Wang L, Gao ZX, Deng XY, Shen KJ, Li YL, et al. Overcoming chemoresistance to b-raf inhibitor in melanoma via targeted inhibition of phosphoenolpyruvate carboxykinase1 using 3-mercaptopropionic acid. Bioengineered. 2022;13(5):13571–86. 10.1080/21655979.2022.2080385. 36700470 10.1080/21655979.2022.2080385 PMC9275918

[263] Patel H, Mishra R, Wier A, Mokhtarpour N, Merino EJ, Garrett JT. RIDR-PI-103, ROS-activated prodrug PI3K inhibitor inhibits cell growth and impairs the PI3K/Akt pathway in BRAF and MEK inhibitor-resistant BRAF-mutant melanoma cells. Anticancer Drugs. 2023;34(4):519–31. 10.1097/cad.0000000000001500. 36847042 10.1097/CAD.0000000000001500 PMC9997637

[264] Qin H, Li Z, Wu J, Liu X, Wang R, Xu J, et al. Diclofenac enhances the response of BRAF inhibitor to melanoma through ROS/p38/p53 signaling. Clin Exp Pharmacol Physiol. 2025;52(3):e70022. 10.1111/1440-1681.70022. 39788129 10.1111/1440-1681.70022

[265] Zhao B, Ban F, Li Y, Shi Q, Guo S, Yi X, et al. Exploiting mitochondrial dysfunction to overcome BRAF inhibitor resistance in advanced melanoma: the role of disulfiram as a copper ionophore. Cell Death Dis. 2025;16(1):482. 10.1038/s41419-025-07766-y. 40592836 10.1038/s41419-025-07766-y PMC12216038

[266] Suzuki Y, Usuki S, Nishizawa M, Tanaka N, Suhara Y, Yajima I. Acyclic retinoid overcomes vemurafenib resistance in melanoma cells via dual inhibition of MAPK and PI3K/AKT/mTOR pathways. Anticancer Res. 2025;45(6):2265–78. 10.21873/anticanres.17601. 40425334 10.21873/anticanres.17601

[267] Cui H, Wang Q, Miller DD, Li W. The tubulin inhibitor VERU-111 in combination with vemurafenib provides an effective treatment of vemurafenib-resistant A375 melanoma. Front Pharmacol. 2021;12:637098. 10.3389/fphar.2021.637098. 33841154 10.3389/fphar.2021.637098 PMC8027488

[268] Wang X, Lama R, Kelleher AD, Rizzo EC, Galster SL, Xue C, et al. Anticancer quinolinol small molecules target multiple pathways to promote cell death and eliminate melanoma cells resistant to BRAF inhibitors. Molecules (Basel, Switzerland). 2025. 10.3390/molecules30132696. 40649216 10.3390/molecules30132696 PMC12251381

[269] Subramanian C, Hohenberger KK, Zuo A, Cousineau E, Blagg B, Cohen M. C-terminal Hsp90 inhibitors overcome MEK and BRAF inhibitor resistance in melanoma. J Cell Mol Med. 2025;29(6):e70489. 10.1111/jcmm.70489. 40135438 10.1111/jcmm.70489 PMC11937850

[270] Vital PDS, Bonatelli M, Dias MP, de Salis LVV, Pinto MT, Baltazar F, et al. 3-bromopyruvate suppresses the malignant phenotype of vemurafenib-resistant melanoma cells. Int J Mol Sci. 2022. 10.3390/ijms232415650. 36555289 10.3390/ijms232415650 PMC9779063

[271] Radi R, Huang C, Elsey J, Jung YH, Corces VG, Arbiser JL. Indolium 1 exerts activity against vemurafenib-resistant melanoma in vivo. Antioxidants (Basel, Switzerland). 2022. 10.3390/antiox11050798. 35624662 10.3390/antiox11050798 PMC9137681

[272] Böcker M, Chatziioannou E, Niessner H, Hirn C, Busch C, Ikenberg K, et al. Ecto-NOX disulfide-thiol exchanger 2 (ENOX2/tNOX) is a potential prognostic marker in primary malignant melanoma and may serve as a therapeutic target. Int J Mol Sci. 2024. 10.3390/ijms252111853. 39519404 10.3390/ijms252111853 PMC11545956

[273] Bergeron C, Bérubé C, Lamb H, Koda Y, Craik DJ, Henriques ST, et al. Analogs of cyclic peptide mortiamide‐D from marine fungi have improved membrane permeability and kill drug‐resistant melanoma cells. Pept Sci. 2024. 10.1002/pep2.24380.

[274] Badawy MAS, Abdel-Aziz M, Abdel-Rahman HM, Ali TFS. Targeting melanoma resistance: novel oxindole and non-oxindole-based benzimidazole derivatives as potent dual inhibitors of BRAF V600E and ABL2 kinases. Eur J Med Chem. 2025;300:118096. 10.1016/j.ejmech.2025.118096. 40911967 10.1016/j.ejmech.2025.118096

[275] Delgado-Goñi T, Galobart TC, Wantuch S, Normantaite D, Leach MO, Whittaker SR, et al. Increased inflammatory lipid metabolism and anaplerotic mitochondrial activation follow acquired resistance to vemurafenib in BRAF-mutant melanoma cells. Br J Cancer. 2020;122(1):72–81. 10.1038/s41416-019-0628-x. 31819183 10.1038/s41416-019-0628-x PMC6964672

[276] Viswanathan VS, Ryan MJ, Dhruv HD, Gill S, Eichhoff OM, Seashore-Ludlow B, et al. Dependency of a therapy-resistant state of cancer cells on a lipid peroxidase pathway. Nature. 2017;547(7664):453–7. 10.1038/nature23007. 28678785 10.1038/nature23007 PMC5667900

[277] Talebi A, Dehairs J, Rambow F, Rogiers A, Nittner D, Derua R, et al. Sustained SREBP-1-dependent lipogenesis as a key mediator of resistance to BRAF-targeted therapy. Nat Commun. 2018;9(1):2500. 10.1038/s41467-018-04664-0. 29950559 10.1038/s41467-018-04664-0 PMC6021375

[278] Vergani E, Beretta GL, Aloisi M, Costantino M, Corno C, Frigerio S, et al. Targeting of the lipid metabolism impairs resistance to BRAF kinase inhibitor in melanoma. Front Cell Dev Biol. 2022;10:927118. 10.3389/fcell.2022.927118. 35912092 10.3389/fcell.2022.927118 PMC9326082

[279] Zanrè V, Bellinato F, Cardile A, Passarini C, Di Bella S, Menegazzi M. BRAF-mutated melanoma cell lines develop distinct molecular signatures after prolonged exposure to AZ628 or dabrafenib: potential benefits of the antiretroviral treatments cabotegravir or doravirine on BRAF-inhibitor-resistant cells. Int J Mol Sci. 2024. 10.3390/ijms252211939. 39596009 10.3390/ijms252211939 PMC11593403

[280] Colakoglu Bergel C, Eryilmaz IE, Cecener G, Egeli U. Second-generation BRAF inhibitor Encorafenib resistance is regulated by NCOA4-mediated iron trafficking in the drug-resistant malignant melanoma cells. Sci Rep. 2025;15(1):2422. 10.1038/s41598-025-86874-3. 39827294 10.1038/s41598-025-86874-3 PMC11742906

[281] Wang L, Otkur W, Wang A, Wang W, Lyu Y, Fang L, et al. Norcantharidin overcomes vemurafenib resistance in melanoma by inhibiting pentose phosphate pathway and lipogenesis via downregulating the mTOR pathway. Front Pharmacol. 2022;13:906043. 10.3389/fphar.2022.906043. 36034784 10.3389/fphar.2022.906043 PMC9411668

[282] Luo X, Duan Y, He J, Huang C, Liu J, Liu Y, et al. Dihydrotanshinone I enhanced BRAF mutant melanoma treatment efficacy by inhibiting the STAT3/SOX2 signaling pathway. Front Oncol. 2025;15:1429018. 10.3389/fonc.2025.1429018. 39944829 10.3389/fonc.2025.1429018 PMC11813777

[283] Foda BM, Baker AE, Joachimiak Ł, Mazur M, Neubig RR. Mechanistic insights into Rho/MRTF inhibition-induced apoptotic events and prevention of drug resistance in melanoma: implications for the involvement of pirin. Front Pharmacol. 2025;16:1505000. 10.3389/fphar.2025.1505000. 39917624 10.3389/fphar.2025.1505000 PMC11799239

[284] Alamodi Alghamdi M, Deshpande H. Selective inhibition of p300 by a novel small molecule EPS496 promotes cell death in vemurafenib-resistant BRAF V600E mutated melanoma cells. Biochem Biophys Res Commun. 2025;750:151382. 10.1016/j.bbrc.2025.151382. 39884005 10.1016/j.bbrc.2025.151382

[285] Gosztyla ML, Zhan L, Olson S, Wei X, Naritomi J, Nguyen G, et al. Integrated multi-omics analysis of zinc-finger proteins uncovers roles in RNA regulation. Mol Cell. 2024;84(19):3826-3842.e3828. 10.1016/j.molcel.2024.08.010. 39303722 10.1016/j.molcel.2024.08.010 PMC11633308

[286] Uebel A, Kewitz-Hempel S, Willscher E, Gebhardt K, Sunderkötter C, Gerloff D. Resistance to BRAF inhibitors: EZH2 and its downstream targets as potential therapeutic options in melanoma. Int J Mol Sci. 2023. 10.3390/ijms24031963. 36768289 10.3390/ijms24031963 PMC9916477

[287] Emran AA, Chatterjee A, Rodger EJ, Tiffen JC, Gallagher SJ, Eccles MR, et al. Targeting DNA methylation and EZH2 activity to overcome melanoma resistance to immunotherapy. Trends Immunol. 2019;40(4):328–44. 10.1016/j.it.2019.02.004. 30853334 10.1016/j.it.2019.02.004

[288] Ding Z, Yang J, Wu B, Wu Y, Guo F. Long non-coding RNA CCHE1 modulates LDHA-mediated glycolysis and confers chemoresistance to melanoma cells. Cancer Metab. 2023;11(1):10. 10.1186/s40170-023-00309-z. 37480145 10.1186/s40170-023-00309-z PMC10360318

[289] Han Y, Fang J, Xiao Z, Deng J, Zhang M, Gu L. Downregulation of lncRNA TSLNC8 promotes melanoma resistance to BRAF inhibitor PLX4720 through binding with PP1α to re-activate MAPK signaling. J Cancer Res Clin Oncol. 2021;147(3):767–77. 10.1007/s00432-020-03484-4. 33389075 10.1007/s00432-020-03484-4 PMC11802067

[290] Shamloo S, Kloetgen A, Petroulia S, Hockemeyer K, Sievers S, Tsirigos A, et al. Integrative CRISPR activation and small molecule inhibitor screening for lncRNA mediating BRAF inhibitor resistance in melanoma. Biomedicines. 2023. 10.3390/biomedicines11072054. 37509693 10.3390/biomedicines11072054 PMC10377043

[291] Katopodi V, Marino A, Pateraki N, Verheyden Y, Cinque S, Jimenez EL, et al. The long non-coding RNA ROSALIND protects the mitochondrial translational machinery from oxidative damage. Cell Death Differ. 2025;32(3):397–415. 10.1038/s41418-024-01377-4. 39294440 10.1038/s41418-024-01377-4 PMC11894192

[292] Gremke N, Polo P, Dort A, Schneikert J, Elmshäuser S, Brehm C, et al. mTOR-mediated cancer drug resistance suppresses autophagy and generates a druggable metabolic vulnerability. Nat Commun. 2020;11(1):4684. 10.1038/s41467-020-18504-7. 32943635 10.1038/s41467-020-18504-7 PMC7499183

[293] Valli F, García Vior MC, Roguin LP, Marino J. Crosstalk between oxidative stress-induced apoptotic and autophagic signaling pathways in Zn(II) phthalocyanine photodynamic therapy of melanoma. Free Radic Biol Med. 2020;152:743–54. 10.1016/j.freeradbiomed.2020.01.018. 31962157 10.1016/j.freeradbiomed.2020.01.018

[294] Wang C, Li Z, Xu P, Xu L, Han S, Sun Y. Combination of polythyleneimine regulating autophagy prodrug and Mdr1 siRNA for tumor multidrug resistance. J Nanobiotechnology. 2022;20(1):476. 10.1186/s12951-022-01689-y. 36369077 10.1186/s12951-022-01689-y PMC9652912

[295] Zhang Y, Liu H, Wang K, Zheng J, Luan H, Xin M. RET inhibitor SPP86 triggers apoptosis and activates the DNA damage response through the suppression of autophagy and the PI3K/AKT signaling pathway in melanoma cells. Drug Des Devel Ther. 2025;19:67–82. 10.2147/dddt.S473390. 39803607 10.2147/DDDT.S473390 PMC11724630

[296] Liu N, Yan M, Lu C, Tao Q, Wu J, Zhou Z, et al. Eravacycline improves the efficacy of anti-PD1 immunotherapy via AP1/CCL5 mediated M1 macrophage polarization in melanoma. Biomaterials. 2025;314:122815. 10.1016/j.biomaterials.2024.122815. 39288620 10.1016/j.biomaterials.2024.122815

[297] Niborski LL, Gueguen P, Ye M, Thiolat A, Ramos RN, Caudana P, et al. CD8+T cell responsiveness to anti-PD-1 is epigenetically regulated by Suv39h1 in melanomas. Nat Commun. 2022;13(1):3739. 10.1038/s41467-022-31504-z. 35768432 10.1038/s41467-022-31504-z PMC9243005

[298] Cucci MA, Grattarola M, Monge C, Roetto A, Barrera G, Caputo E, et al. Nrf2 as a therapeutic target in the resistance to targeted therapies in melanoma. Antioxidants (Basel, Switzerland). 2023. 10.3390/antiox12061313. 37372043 10.3390/antiox12061313 PMC10294952

[299] Garbarino O, Valenti GE, Monteleone L, Pietra G, Mingari MC, Benzi A, et al. PLX4032 resistance of patient-derived melanoma cells: crucial role of oxidative metabolism. Front Oncol. 2023;13:1210130. 10.3389/fonc.2023.1210130. 37534247 10.3389/fonc.2023.1210130 PMC10391174

[300] Wang X, Zhong F, Chen T, Wang H, Wang W, Jin H, et al. Cholesterol neutralized vemurafenib treatment by promoting melanoma stem-like cells via its metabolite 27-hydroxycholesterol. Cell Mol Life Sci. 2024;81(1):226. 10.1007/s00018-024-05267-3. 38775844 10.1007/s00018-024-05267-3 PMC11111659

[301] Roller A, Davydov II, Schwalie PC, Serrano-Serrano ML, Heller A, Staedler N, et al. Tumor-agnostic transcriptome-based classifier identifies spatial infiltration patterns of CD8+T cells in the tumor microenvironment and predicts clinical outcome in early-phase and late-phase clinical trials. J Immunother Cancer. 2024. 10.1136/jitc-2023-008185. 38649280 10.1136/jitc-2023-008185 PMC11043740

[302] Yu J, Wu X, Song J, Zhao Y, Li H, Luo M, et al. Loss of MHC-I antigen presentation correlated with immune checkpoint blockade tolerance in MAPK inhibitor-resistant melanoma. Front Pharmacol. 2022;13:928226. 10.3389/fphar.2022.928226. 36091815 10.3389/fphar.2022.928226 PMC9459091

[303] Zhang J, Wang S, Guo X, Lu Y, Liu X, Jiang M, et al. Arginine supplementation targeting tumor-killing immune cells reconstructs the tumor microenvironment and enhances the antitumor immune response. ACS Nano. 2022;16(8):12964–78. 10.1021/acsnano.2c05408. 35968927 10.1021/acsnano.2c05408

[304] Zhang Y, Vilalta M, Tang L, Sharma R, Pfister SX. Radiotherapy shows synergistic anti-tumor efficacy with Treg depletion by reprogramming the immune-excluded tumor microenvironment. Int J Radiat Oncol Biol Phys. 2023;117(2S):S72. 10.1016/j.ijrobp.2023.06.381.

[305] Algazi AP, Moon J, Lao CD, Chmielowski B, Kendra KL, Lewis KD, et al. A phase 1 study of triple-targeted therapy with BRAF, MEK, and AKT inhibitors for patients with BRAF-mutated cancers. Cancer. 2024;130(10):1784–96. 10.1002/cncr.35200. 38261444 10.1002/cncr.35200 PMC13225457

[306] Dirven I, Pierre E, Vander Mijnsbrugge AS, Vounckx M, Kessels JI, Neyns B. Regorafenib combined with BRAF/MEK inhibitors for the treatment of refractory melanoma brain metastases. Cancers (Basel). 2024. 10.3390/cancers16234083. 39682270 10.3390/cancers16234083 PMC11640054

[307] Frazao A, Rethacker L, Jeudy G, Colombo M, Pasmant E, Avril MF, et al. BRAF inhibitor resistance of melanoma cells triggers increased susceptibility to natural killer cell-mediated lysis. J Immunother Cancer. 2020. 10.1136/jitc-2019-000275. 32912923 10.1136/jitc-2019-000275 PMC7482503

[308] Mukhtar AB, Morgan HJ, Gibbs A, Davies GE, Lovatt C, Patel GK. Targeting CD20-expressing malignant melanoma cells augments BRAF inhibitor killing. Br J Dermatol. 2024;190(5):729–39. 10.1093/bjd/ljad502. 38288865 10.1093/bjd/ljad502

[309] Robert C, Lewis KD, Gutzmer R, Stroyakovskiy D, Gogas H, Protsenko S, et al. Biomarkers of treatment benefit with atezolizumab plus vemurafenib plus cobimetinib in BRAF V600 mutation-positive melanoma. Ann Oncol. 2022;33(5):544–55. 10.1016/j.annonc.2022.01.076. 35131452 10.1016/j.annonc.2022.01.076

[310] Sullivan RJ, Hamid O, Gonzalez R, Infante JR, Patel MR, Hodi FS, et al. Atezolizumab plus cobimetinib and vemurafenib in BRAF-mutated melanoma patients. Nat Med. 2019;25(6):929–35. 10.1038/s41591-019-0474-7. 31171876 10.1038/s41591-019-0474-7

[311] Flaherty KT. A twenty year perspective on melanoma therapy. Pigment Cell Melanoma Res. 2023;36(6):563–75. 10.1111/pcmr.13125. 37770281 10.1111/pcmr.13125

[312] Yuksel HC, Acar C, Sahin G, Celebi G, Tunbekici S, Karaca BS. LASSO-driven selection of biochemical and clinical markers for primary resistance to PD-1 inhibitors in metastatic melanoma. Medicina Kaunas. 2025. 10.3390/medicina61091559. 41010951 10.3390/medicina61091559 PMC12472068

[313] Patel RP, Lim LRJ, Saleh R, Schenk D, Lee MK, Lelliott E, et al. Sensitivity to immune checkpoint inhibitors in BRAF/MEK inhibitor refractory melanoma. J Immunother Cancer. 2025. 10.1136/jitc-2025-011551. 40379272 10.1136/jitc-2025-011551 PMC12083385

[314] Rogala P, Czarnecka AM, Cybulska-Stopa B, Ostaszewski K, Piejko K, Ziętek M, et al. Long term results and prognostic biomarkers for anti-PD1 immunotherapy used after BRAFi/MEKi combination in advanced cutaneous melanoma patients. Cancers (Basel). 2022. 10.3390/cancers14092123. 35565255 10.3390/cancers14092123 PMC9101360

[315] Kreft S, Gesierich A, Eigentler T, Franklin C, Valpione S, Ugurel S, et al. Efficacy of PD-1-based immunotherapy after radiologic progression on targeted therapy in stage IV melanoma. Eur J Cancer (Oxford, England : 1990). 2019;116:207–15. 10.1016/j.ejca.2019.05.015. 10.1016/j.ejca.2019.05.015 31212163

[316] Liu S, Liu H, Song X, Jiang A, Deng Y, Yang C, et al. Adoptive CD8 + T-cell grafted with liposomal immunotherapy drugs to counteract the immune suppressive tumor microenvironment and enhance therapy for melanoma. Nanoscale. 2021;13(37):15789–803. 10.1039/d1nr04036g. 34528979 10.1039/d1nr04036g

[317] Barras D, Ghisoni E, Chiffelle J, Orcurto A, Dagher J, Fahr N, et al. Response to tumor-infiltrating lymphocyte adoptive therapy is associated with preexisting CD8 + T-myeloid cell networks in melanoma. Sci Immunol. 2024;9(92):eadg7995. 10.1126/sciimmunol.adg7995. 38306416 10.1126/sciimmunol.adg7995

[318] Kristensen NP, Heeke C, Tvingsholm SA, Borch A, Draghi A, Crowther MD, et al. Neoantigen-reactive CD8+ T cells affect clinical outcome of adoptive cell therapy with tumor-infiltrating lymphocytes in melanoma. J Clin Invest. 2022. 10.1172/jci150535. 34813506 10.1172/JCI150535 PMC8759789

[319] Foda BM, Misek SA, Gallo KA, Neubig RR. Inhibition of the Rho/MRTF pathway improves the response of BRAF-resistant melanoma to PD1/PDL1 blockade. Int J Cancer. 2024;155(7):1303–15. 10.1002/ijc.35056. 38898604 10.1002/ijc.35056

[320] Hornsteiner F, Vierthaler J, Strandt H, Resag A, Fu Z, Ausserhofer M, et al. Tumor-targeted therapy with BRAF-inhibitor recruits activated dendritic cells to promote tumor immunity in melanoma. J Immunother Cancer. 2024. 10.1136/jitc-2023-008606. 38631706 10.1136/jitc-2023-008606 PMC11029477

[321] Ribas A, Lawrence D, Atkinson V, Agarwal S, Miller WH, Carlino MS, et al. Combined BRAF and MEK inhibition with PD-1 blockade immunotherapy in BRAF-mutant melanoma. Nat Med. 2019;25(6):936–40. 10.1038/s41591-019-0476-5. 31171879 10.1038/s41591-019-0476-5 PMC8562134

[322] Kim TW, Kim Y, Keum H, Jung W, Kang M, Jon S. Combination of a STAT3 inhibitor with anti-PD-1 immunotherapy is an effective treatment regimen for a vemurafenib-resistant melanoma. Mol Ther Oncolytics. 2022;26:1–14. 10.1016/j.omto.2022.06.001. 35784401 10.1016/j.omto.2022.06.001 PMC9218293

[323] Huang L, Xu Y, Fang J, Liu W, Chen J, Liu Z, et al. Targeting STAT3 abrogates Tim-3 upregulation of adaptive resistance to PD-1 blockade on regulatory T cells of melanoma. Front Immunol. 2021;12:654749. 10.3389/fimmu.2021.654749. 33936081 10.3389/fimmu.2021.654749 PMC8082190

[324] Chen J, Liang Y, Li J, Wu F, Wu N, Liu S, et al. Diosmetin augments BRAF-targeted therapy via concurrent suppression of MAPK and STAT3 pathways in melanoma. Eur J Pharmacol. 2025;1004:178008. 10.1016/j.ejphar.2025.178008. 40744388 10.1016/j.ejphar.2025.178008

[325] Nakamura K, Yaguchi T, Murata M, Ota Y, Mikoshiba A, Kiniwa Y, et al. Tumor eradication by triplet therapy with BRAF inhibitor, TLR 7 agonist, and PD-1 antibody for BRAF-mutated melanoma. Cancer Sci. 2024;115(9):2879–92. 10.1111/cas.16251. 38894534 10.1111/cas.16251 PMC11462939

[326] Dréno B, Khammari A, Fortun A, Vignard V, Saiagh S, Beauvais T, et al. Phase I/II clinical trial of adoptive cell transfer of sorted specific T cells for metastatic melanoma patients. Cancer Immunol Immunother CII. 2021;70(10):3015–30. 10.1007/s00262-021-02961-0. 34120214 10.1007/s00262-021-02961-0 PMC8423703

[327] Adeshakin AO, Adeshakin FO, Yan D, Wan X. Regulating histone deacetylase signaling pathways of myeloid-derived suppressor cells enhanced T cell-based immunotherapy. Front Immunol. 2022;13:781660. 10.3389/fimmu.2022.781660. 35140716 10.3389/fimmu.2022.781660 PMC8818783

[328] Yeon M, Kim Y, Jung HS, Jeoung D. Histone deacetylase inhibitors to overcome resistance to targeted and immuno therapy in metastatic melanoma. Front Cell Dev Biol. 2020;8:486. 10.3389/fcell.2020.00486. 32626712 10.3389/fcell.2020.00486 PMC7311641

[329] Sun Z, Shi M, Xia J, Li X, Chen N, Wang H, et al. HDAC and MEK inhibition synergistically suppresses HOXC6 and enhances PD-1 blockade efficacy in BRAF V600E -mutant microsatellite stable colorectal cancer. J Immunother Cancer. 2025. 10.1136/jitc-2024-010460. 39800382 10.1136/jitc-2024-010460 PMC11749543

[330] Goh CJH, Wong JH, El Farran C, Tan BX, Coffill CR, Loh YH, et al. Identification of pathways modulating vemurafenib resistance in melanoma cells via a genome-wide CRISPR/Cas9 screen. G3 Genes|Genomes|Genetics. 2021. 10.1093/g3journal/jkaa069.35382356

[331] Wen X, Han M, Hosoya M, Toshima R, Onishi M, Fujii T, et al. Identification of BRAF inhibitor resistance-associated lncRNAs using genome-scale CRISPR-Cas9 transcriptional activation screening. Anticancer Res. 2024;44(6):2349–58. 10.21873/anticanres.17042. 38821628 10.21873/anticanres.17042

[332] Simbulan-Rosenthal CM, Haribabu Y, Vakili S, Kuo LW, Clark H, Dougherty R, et al. Employing CRISPR-Cas9 to generate CD133 synthetic lethal melanoma stem cells. Int J Mol Sci. 2022. 10.3390/ijms23042333. 35216449 10.3390/ijms23042333 PMC8877091

[333] Imani S, Roozitalab G, Emadi M, Moradi A, Behzadi P, Jabbarzadeh Kaboli P. The evolution of BRAF-targeted therapies in melanoma: overcoming hurdles and unleashing novel strategies. Front Oncol. 2024;14:1504142. 10.3389/fonc.2024.1504142. 39582535 10.3389/fonc.2024.1504142 PMC11582033

[334] Beretti F, Gatti M, Zavatti M, Bassoli S, Pellacani G, Maraldi T. Reactive oxygen species regulation of chemoresistance and metastatic capacity of melanoma: role of the cancer stem cell marker CD271. Biomedicines. 2023. 10.3390/biomedicines11041229. 37189846 10.3390/biomedicines11041229 PMC10136133

[335] Yang X, Zhou S, Zeng J, Zhang S, Li M, Yue F, et al. A biodegradable lipid nanoparticle delivers a Cas9 ribonucleoprotein for efficient and safe in situ genome editing in melanoma. Acta Biomater. 2024;190:531–47. 10.1016/j.actbio.2024.10.030. 39461690 10.1016/j.actbio.2024.10.030

[336] Wei Zhao, La Zhang, Jiao Guo, Qing Xu, Mi Zhang, Hongqing Liu, et al. Intelligent Nano‐Cage for Precision Delivery of CRISPR‐Cas9 and ACC Inhibitors to Enhance Antitumor Cascade Therapy Through Lipid Metabolism Disruption. Adv Function Mater. 2025;35(13). 10.1002/adfm.202418090.

[337] Zhang LG, Wang P, Feng Q, Wang NX, Chen ZT, Huang YY, et al. Lipid nanoparticle-mediated efficient delivery of CRISPR/Cas9 for tumor therapy. NPG Asia Mater. 2017;9(10):e441-e441. 10.1038/am.2017.185.

[338] Saraswat A, Vartak R, Hegazy R, Fu Y, Rao TJR, Billack B, et al. Oral lipid nanocomplex of BRD4 PROteolysis TArgeting Chimera and vemurafenib for drug-resistant malignant melanoma. Biomed Pharmacother. 2023;168:115754. 10.1016/j.biopha.2023.115754. 37871557 10.1016/j.biopha.2023.115754

[339] Saraswat A, Vemana HP, Dukhande V, Patel K. Novel gene therapy for drug-resistant melanoma: synergistic combination of *PTEN* plasmid and BRD4 PROTAC-loaded lipid nanocarriers. Mol Ther Nucleic Acids. 2024;35(3):102292. 10.1016/j.omtn.2024.102292. 39238805 10.1016/j.omtn.2024.102292 PMC11374965

[340] Loureiro JB, Raimundo L, Calheiros J, Carvalho C, Barcherini V, Lima NR, et al. Targeting p53 for melanoma treatment: counteracting tumour proliferation, dissemination and therapeutic resistance. Cancers (Basel). 2021. 10.3390/cancers13071648. 33916029 10.3390/cancers13071648 PMC8037490

[341] Vlašić I, Horvat A, Tadijan A, Slade N. p53 family in resistance to targeted therapy of melanoma. Int J Mol Sci. 2022. 10.3390/ijms24010065. 36613518 10.3390/ijms24010065 PMC9820688

[342] Shirley M. Encorafenib and binimetinib: first global approvals. Drugs. 2018;78(12):1277–84. 10.1007/s40265-018-0963-x. 30117021 10.1007/s40265-018-0963-x

[343] Márquez-Rodas I, Álvarez A, Arance A, Valduvieco I, Berciano-Guerrero MÁ, Delgado R, et al. Encorafenib and binimetinib followed by radiotherapy for patients with BRAFV600-mutant melanoma and brain metastases (E-BRAIN/GEM1802 phase II study). Neuro Oncol. 2024;26(11):2074–83. 10.1093/neuonc/noae116. 38946469 10.1093/neuonc/noae116 PMC11534317

[344] Morris VK, Parseghian CM, Bahrambeigi V, Abdelfattah N, Xiao L, Agrawal A, et al. Phase 1/2 trial of encorafenib, cetuximab, and nivolumab in microsatellite stable BRAF metastatic colorectal cancer. Cancer Cell. 2025. 10.1016/j.ccell.2025.08.002. 40882637 10.1016/j.ccell.2025.08.002 PMC12431688

[345] Zhang Y, Vagiannis D, Budagaga Y, Sabet Z, Hanke I, Rozkoš T, et al. Encorafenib acts as a dual-activity chemosensitizer through its inhibitory effect on ABCC1 transporter in vitro and ex vivo. Pharmaceutics. 2022. 10.3390/pharmaceutics14122595. 36559089 10.3390/pharmaceutics14122595 PMC9785850

[346] Car I, Dittmann A, Vasieva O, Bočkor L, Grbčić P, Piteša N, et al. Ezrin inhibition overcomes acquired resistance to vemurafenib in BRAFV600E-mutated colon cancer and melanoma cells in vitro. Int J Mol Sci. 2023. 10.3390/ijms241612906. 37629086 10.3390/ijms241612906 PMC10454476

[347] Guo S, Yue Q, Wang S, Wang H, Ye Z, Zhang W, et al. Sestrin2 contributes to BRAF inhibitor resistance via reducing redox vulnerability of melanoma cells. J Dermatol Sci. 2023;109(2):52–60. 10.1016/j.jdermsci.2022.12.007. 36858850 10.1016/j.jdermsci.2022.12.007

[348] Irie N, Mizoguchi K, Warita T, Nakano M, Sasaki K, Tashiro J, et al. Repurposing of the cardiovascular drug statin for the treatment of cancers: efficacy of statin-dipyridamole combination treatment in melanoma cell lines. Biomedicines. 2024. 10.3390/biomedicines12030698. 38540310 10.3390/biomedicines12030698 PMC10968169

[349] Guzzetti C, Corno C, Vergani E, Mirra L, Ciusani E, Rodolfo M, et al. Kisspeptin-mediated improvement of sensitivity to BRAF inhibitors in vemurafenib-resistant melanoma cells. Front Oncol. 2023;13:1182853. 10.3389/fonc.2023.1182853. 37790750 10.3389/fonc.2023.1182853 PMC10544897

[350] Varela-Vázquez A, Guitián-Caamaño A, Carpintero-Fernández P, Carneiro-Figueira A, Álvarez V, Varela-Eirín M, et al. Cx43 enhances response to BRAF/MEK inhibitors by reducing DNA repair capacity. Nat Commun. 2025;16(1):6168. 10.1038/s41467-025-60971-3. 40615399 10.1038/s41467-025-60971-3 PMC12227752

[351] Yaeger R, McKean MA, Haq R, Beck JT, Taylor MH, Cohen JE, et al. A next-generation BRAF inhibitor overcomes resistance to BRAF inhibition in patients with BRAF-mutant cancers using pharmacokinetics-informed dose escalation. Cancer Discov. 2024;14(9):1599–611. 10.1158/2159-8290.Cd-24-0024. 38691346 10.1158/2159-8290.CD-24-0024 PMC11372368

[352] Kraft O, Hartmann AK, Brandt S, Hoenke S, Heise NV, Csuk R, et al. Asiatic acid as a leading structure for derivatives combining sub-nanomolar cytotoxicity, high selectivity, and the ability to overcome drug resistance in human preclinical tumor models. Eur J Med Chem. 2023;250:115189. 10.1016/j.ejmech.2023.115189. 36780832 10.1016/j.ejmech.2023.115189

[353] Mbaveng AT, Chi GF, Bonsou IN, Abdelfatah S, Tamfu AN, Yeboah EMO, et al. N-acetylglycoside of oleanolic acid (aridanin) displays promising cytotoxicity towards human and animal cancer cells, inducing apoptotic, ferroptotic and necroptotic cell death. Phytomedicine. 2020;76:153261. 10.1016/j.phymed.2020.153261. 32559584 10.1016/j.phymed.2020.153261

[354] Coricovac D, Dehelean CA, Pinzaru I, Mioc A, Aburel OM, Macasoi I, et al. Assessment of betulinic acid cytotoxicity and mitochondrial metabolism impairment in a human melanoma cell line. Int J Mol Sci. 2021. 10.3390/ijms22094870. 34064489 10.3390/ijms22094870 PMC8125295

[355] Aires-Lopes B, Justo GZ, Cordeiro HG, Durán N, Azevedo-Martins JM, Ferreira Halder CV. Violacein improves vemurafenib response in melanoma spheroids. Nat Prod Res. 2024;38(19):3417–20. 10.1080/14786419.2023.2244134. 37571995 10.1080/14786419.2023.2244134

[356] Cvetanova B, Shen YC, Shyur LF. Cumingianoside A, a phyto-triterpenoid saponin inhibits acquired BRAF inhibitor resistant melanoma growth via programmed cell death. Front Pharmacol. 2019;10:30. 10.3389/fphar.2019.00030. 30745871 10.3389/fphar.2019.00030 PMC6360185

[357] Liu N, Wang KS, Qi M, Zhou YJ, Zeng GY, Tao J, et al. Vitexin compound 1, a novel extraction from a Chinese herb, suppresses melanoma cell growth through DNA damage by increasing ROS levels. J Exp Clin Cancer Res CR. 2018;37(1):269. 10.1186/s13046-018-0897-x. 30400954 10.1186/s13046-018-0897-x PMC6219156

[358] Chiu YJ, Yang JS, Tsai FJ, Chiu HY, Juan YN, Lo YH, et al. Curcumin suppresses cell proliferation and triggers apoptosis in vemurafenib-resistant melanoma cells by downregulating the EGFR signaling pathway. Environ Toxicol. 2022. 10.1002/tox.23450. 34994998 10.1002/tox.23450

[359] Yu P, Wei H, Li K, Zhu S, Li J, Chen C, et al. The traditional Chinese medicine monomer Ailanthone improves the therapeutic efficacy of anti-PD-L1 in melanoma cells by targeting c-Jun. J Exp Clin Cancer Res. 2022;41(1):346. 10.1186/s13046-022-02559-z. 36522774 10.1186/s13046-022-02559-z PMC9753288

[360] Benfield AH, Vernen F, Young RSE, Nadal-Bufí F, Lamb H, Hammerlindl H, et al. Cyclic tachyplesin I kills proliferative, non-proliferative and drug-resistant melanoma cells without inducing resistance. Pharmacol Res. 2024;207:107298. 10.1016/j.phrs.2024.107298. 39032840 10.1016/j.phrs.2024.107298

[361] Stergiopoulou D, Smilkou S, Georgoulias V, Kaklamanis L, Lianidou E, Markou A. Development and validation of a novel dual-drop-off ddPCR assay for the simultaneous detection of ten hotspots PIK3CA mutations. Anal Chem. 2023;95(37):14068–76. 10.1021/acs.analchem.3c02692. 37681347 10.1021/acs.analchem.3c02692

[362] Magbanua MJM, Swigart LB, Wu HT, Hirst GL, Yau C, Wolf DM, et al. Circulating tumor DNA in neoadjuvant-treated breast cancer reflects response and survival. Ann Oncol. 2021;32(2):229–39. 10.1016/j.annonc.2020.11.007. 33232761 10.1016/j.annonc.2020.11.007 PMC9348585

[363] Vanni I, Pastorino L, Tanda ET, Andreotti V, Dalmasso B, Solari N, et al. Whole-exome sequencing and cfDNA analysis uncover genetic determinants of melanoma therapy response in a real-world setting. Int J Mol Sci. 2023. 10.3390/ijms24054302. 36901733 10.3390/ijms24054302 PMC10002464

[364] Ita MI, Wang JH, Heffron CC, Power DG, Nolan Y, Toulouse A, et al. Therapeutic response evaluation in advanced melanoma patients incorporating plasma cfDNA, LDH, VEGF, PD-L1, and IFN-γ measurements. Anticancer Res. 2022;42(2):801–10. 10.21873/anticanres.15538. 35093878 10.21873/anticanres.15538

[365] Ma Y, Chu Y, Xu Z, Xie C, Ma X, Zhang L, et al. Ultrafast and highly specific detection of one-base mutated cell-free DNA at a very low abundance. Anal Chem. 2024;96(1):117–26. 10.1021/acs.analchem.3c03326. 38114445 10.1021/acs.analchem.3c03326

[366] Silva S, Danson S, Teare D, Taylor F, Bradford J, McDonagh AJG, et al. Genome-wide analysis of circulating cell-free DNA copy number detects active melanoma and predicts survival. Clin Chem. 2018;64(9):1338–46. 10.1373/clinchem.2018.290023. 29941468 10.1373/clinchem.2018.290023

[367] Gupta PK, Orlovskiy S, Arias-Mendoza F, Nelson DS, Osborne A, Pickup S, et al. Metabolic imaging biomarkers of response to signaling inhibition therapy in melanoma. Cancers (Basel). 2024. 10.3390/cancers16020365. 38254853 10.3390/cancers16020365 PMC10814512

[368] Peng J, Lin Z, Chen W, Ruan J, Deng F, Yao L, et al. Vemurafenib induces a noncanonical senescence-associated secretory phenotype in melanoma cells which promotes vemurafenib resistance. Heliyon. 2023;9(7):e17714. 10.1016/j.heliyon.2023.e17714. 37456058 10.1016/j.heliyon.2023.e17714 PMC10345356

[369] Tomás A, Roque L, Francisco I, Silva AL, Nunes H, Gouveia E, et al. A novel patient-derived cutaneous melanoma cell line reveals key features of metastatic melanoma. Front Oncol. 2025;15:1531013. 10.3389/fonc.2025.1531013. 40756116 10.3389/fonc.2025.1531013 PMC12313697

[370] Zhang Y, Zhang C, He J, Lai G, Li W, Zeng H, et al. Comprehensive analysis of single cell and bulk RNA sequencing reveals the heterogeneity of melanoma tumor microenvironment and predicts the response of immunotherapy. Inflamm Res. 2024;73(8):1393–409. 10.1007/s00011-024-01905-5. 38896289 10.1007/s00011-024-01905-5

[371] Lim SY, Lin Y, Lee JH, Pedersen B, Stewart A, Scolyer RA, et al. Single-cell RNA sequencing reveals melanoma cell state-dependent heterogeneity of response to MAPK inhibitors. EBioMedicine. 2024;107:105308. 10.1016/j.ebiom.2024.105308. 39216232 10.1016/j.ebiom.2024.105308 PMC11402938

[372] Randic T, Magni S, Philippidou D, Margue C, Grzyb K, Preis JR, et al. Single-cell transcriptomics of NRAS-mutated melanoma transitioning to drug resistance reveals P2RX7 as an indicator of early drug response. Cell Rep. 2023;42(7):112696. 10.1016/j.celrep.2023.112696. 37379213 10.1016/j.celrep.2023.112696

[373] Zhang T, Jia H, Song T, Lv L, Gulhan DC, Wang H, et al. De novo identification of expressed cancer somatic mutations from single-cell RNA sequencing data. Genome Med. 2023;15(1):115. 10.1186/s13073-023-01269-1. 38111063 10.1186/s13073-023-01269-1 PMC10726641

[374] Saraswat A, Patel K. Development and in-depth characterization of BRAFi-resistant melanoma cell lines in vitro and in vivo. Exp Cell Res. 2024;438(1):114033. 10.1016/j.yexcr.2024.114033. 38593916 10.1016/j.yexcr.2024.114033

[375] Ho YJ, Anaparthy N, Molik D, Mathew G, Aicher T, Patel A, et al. Single-cell RNA-seq analysis identifies markers of resistance to targeted BRAF inhibitors in melanoma cell populations. Genome Res. 2018;28(9):1353–63. 10.1101/gr.234062.117. 30061114 10.1101/gr.234062.117 PMC6120620

[376] Purwin TJ, Caksa S, Sacan A, Capparelli C, Aplin AE. Gene signature reveals decreased SOX10-dependent transcripts in malignant cells from immune checkpoint inhibitor-resistant cutaneous melanomas. iScience. 2023;26(9):107472. 10.1016/j.isci.2023.107472. 37636077 10.1016/j.isci.2023.107472 PMC10450419

[377] Xie J, Zhang P, Tang Q, Ma C, Li M, Qi M. Leveraging single-cell sequencing analysis and bulk-RNA sequencing analysis to forecast necroptosis in cutaneous melanoma prognosis. Exp Dermatol. 2024;33(7):e15148. 10.1111/exd.15148. 39051739 10.1111/exd.15148

[378] Ding Y, Zhao Z, Cai H, Zhou Y, Chen H, Bai Y, et al. Single-cell sequencing analysis related to sphingolipid metabolism guides immunotherapy and prognosis of skin cutaneous melanoma. Front Immunol. 2023;14:1304466. 10.3389/fimmu.2023.1304466. 38077400 10.3389/fimmu.2023.1304466 PMC10701528

[379] Wu SZ, Roden DL, Al-Eryani G, Bartonicek N, Harvey K, Cazet AS, et al. Cryopreservation of human cancers conserves tumour heterogeneity for single-cell multi-omics analysis. Genome Med. 2021;13(1):81. 10.1186/s13073-021-00885-z. 33971952 10.1186/s13073-021-00885-z PMC8111910

[380] Wang P, Ma Y, Zhao Y, Li Y, Tang C, Wang S, et al. Single-cell RNA sequencing unveils tumor heterogeneity and immune microenvironment between subungual and plantar melanoma. Sci Rep. 2024;14(1):7039. 10.1038/s41598-024-57640-8. 38528036 10.1038/s41598-024-57640-8 PMC10963724

[381] Luo S, Wang D, Chen J, Hong S, Fang Y, Cao L, et al. The combination of single-cell and RNA sequencing analysis decodes the melanoma tumor microenvironment and identifies novel T cell-associated signature genes. J Cancer. 2024;15(15):5085–100. 10.7150/jca.96484. 39132169 10.7150/jca.96484 PMC11310880

[382] Zhong G, Wang Q, Wang Y, Guo Y, Xu M, Guan Y, et al. Scrna-seq reveals ATPIF1 activity in control of T cell antitumor activity. Oncoimmunology. 2022;11(1):2114740. 10.1080/2162402x.2022.2114740. 36016697 10.1080/2162402X.2022.2114740 PMC9397437

[383] Sun D, Guan X, Moran AE, Wu LY, Qian DZ, Schedin P, et al. Identifying phenotype-associated subpopulations by integrating bulk and single-cell sequencing data. Nat Biotechnol. 2022;40(4):527–38. 10.1038/s41587-021-01091-3. 34764492 10.1038/s41587-021-01091-3 PMC9010342

[384] Lian W, Xiang P, Ye C, Xiong J. Single-cell RNA sequencing analysis reveals the role of cancerassociated fibroblasts in skin melanoma. Curr Med Chem. 2024;31(42):7015–29. 10.2174/0109298673282799231211113347. 38173195 10.2174/0109298673282799231211113347

[385] Tirosh I, Izar B, Prakadan SM, Wadsworth MH, Treacy D, Trombetta JJ, et al. Dissecting the multicellular ecosystem of metastatic melanoma by single-cell RNA-seq. Science. 2016;352(6282):189–96. 10.1126/science.aad0501. 27124452 10.1126/science.aad0501 PMC4944528

[386] Zhang C, Yang J, Chen S, Sun L, Li K, Lai G, et al. Artificial intelligence in ovarian cancer drug resistance advanced 3PM approach: subtype classification and prognostic modeling. EPMA J. 2024;15(3):525–44. 10.1007/s13167-024-00374-4. 39239109 10.1007/s13167-024-00374-4 PMC11371997

[387] Zhang C, Yuan Y, Xia Q, Wang J, Xu K, Gong Z, et al. Machine learning-driven prediction, preparation, and evaluation of functional nanomedicines via drug-drug self-assembly. Adv Sci (Weinheim, Baden-Wurttemberg, Germany). 2025;12(9):e2415902. 10.1002/advs.202415902. 10.1002/advs.202415902 PMC11884566 39792782

[388] Goetz A, Shanahan F, Brooks L, Lin E, Mroue R, Dela Cruz D, et al. Computational modeling of drug response identifies mutant-specific constraints for dosing panRAF and MEK inhibitors in melanoma. Cancers (Basel). 2024. 10.3390/cancers16162914. 39199684 10.3390/cancers16162914 PMC11353013

[389] Sahni S, Wang B, Wu D, Dhruba SR, Nagy M, Patkar S, et al. A machine learning model reveals expansive downregulation of ligand-receptor interactions that enhance lymphocyte infiltration in melanoma with developed resistance to immune checkpoint blockade. Nat Commun. 2024;15(1):8867. 10.1038/s41467-024-52555-4. 39402030 10.1038/s41467-024-52555-4 PMC11473774

[390] Abd El-Hafeez T, Shams MY, Elshaier YAMM, Farghaly HM, Hassanien AE. Harnessing machine learning to find synergistic combinations for FDA-approved cancer drugs. Sci Rep. 2024;14(1):2428. 10.1038/s41598-024-52814-w. 38287066 10.1038/s41598-024-52814-w PMC10825182

[391] Rao KN, Fernandez-Alvarez V, Guntinas-Lichius O, Sreeram MP, de Bree R, Kowalski LP, et al. The limitations of artificial intelligence in head and neck oncology. Adv Ther. 2025;42(6):2559–68. 10.1007/s12325-025-03198-4. 40299277 10.1007/s12325-025-03198-4 PMC12085315

[392] Huang J, Xiang Y, Gan S, Wu L, Yan J, Ye D, et al. Application of artificial intelligence in medical imaging for tumor diagnosis and treatment: a comprehensive approach. Discov Oncol. 2025;16(1):1625. 10.1007/s12672-025-03307-3. 40856916 10.1007/s12672-025-03307-3 PMC12381339

[393] van der Hiel B, van de Wit- r Veen BJ, van den Eertwegh AJM, Vogel WV, Stokkel MPM, Lopez-Yurda M, et al. Metabolic parameters on baseline and early [ 18 F]FDG PET/CT as a predictive biomarker for resistance to BRAF/MEK inhibition in advanced cutaneous BRAFV600-mutated melanoma. EJNMMI Res. 2025;15(1):60. 10.1186/s13550-025-01259-x. 40434500 10.1186/s13550-025-01259-x PMC12119442

[394] Wagner NB, Lenders MM, Kühl K, Reinhardt L, Fuchß M, Ring N, et al. Baseline metastatic growth rate is an independent prognostic marker in patients with advanced BRAF V600 mutated melanoma receiving targeted therapy. Eur J Cancer (Oxford, England : 1990). 2024;196:113425. 10.1016/j.ejca.2023.113425. 10.1016/j.ejca.2023.113425 38039778

[395] O’Brien MT, Iwamoto S, Haq R, Johnson DB. Re-re-treatment?" Third and fourth courses of BRAF/MEK inhibition in advanced melanoma. Eur J Cancer. 2025;220:115378. 10.1016/j.ejca.2025.115378. 40157811 10.1016/j.ejca.2025.115378

[396] Demidov L, Samoylenko I, Kharkevich G, Orlova K, Moiseenko V, Utyashev I, et al. Improved clinical outcomes with low-dose anti-CTLA-4 (Nurulimab) plus anti-PD-1 (Prolgolimab) vs. anti-PD-1 monotherapy in advanced cutaneous melanoma: results from the phase III OCTAVA trial. Eur J Cancer (Oxford, England : 1990). 2025;227:115674. 10.1016/j.ejca.2025.115674. 10.1016/j.ejca.2025.115674 40795521

[397] Dummer R, Lebbé C, Atkinson V, Mandalà M, Nathan PD, Arance A, et al. Combined PD-1, BRAF and MEK inhibition in advanced BRAF-mutant melanoma: safety run-in and biomarker cohorts of COMBI-i. Nat Med. 2020;26(10):1557–63. 10.1038/s41591-020-1082-2. 33020648 10.1038/s41591-020-1082-2

[398] Piejko K, Cybulska-Stopa B, Ziętek M, Dziura R, Galus Ł, Kempa-Kamińska N, et al. Long-term real-world outcomes and safety of vemurafenib and vemurafenib + cobimetinib therapy in patients with BRAF-mutated melanoma. Target Oncol. 2023;18(2):235–45. 10.1007/s11523-023-00954-w. 36906728 10.1007/s11523-023-00954-w PMC10042754

[399] Ribas A, Daud A, Pavlick AC, Gonzalez R, Lewis KD, Hamid O, et al. Extended 5-year follow-up results of a phase Ib study (BRIM7) of vemurafenib and cobimetinib in BRAF -mutant melanoma. Clin Cancer Res. 2020;26(1):46–53. 10.1158/1078-0432.Ccr-18-4180. 31732523 10.1158/1078-0432.CCR-18-4180 PMC6942621

[400] Long GV, Carlino MS, Au-Yeung G, Spillane AJ, Shannon KF, Gyorki DE, et al. Neoadjuvant pembrolizumab, dabrafenib and trametinib in BRAF V600 -mutant resectable melanoma: the randomized phase 2 NeoTrio trial. Nat Med. 2024;30(9):2540–8. 10.1038/s41591-024-03077-5. 38907159 10.1038/s41591-024-03077-5 PMC11405264

[401] Ascierto PA, Stroyakovskiy D, Gogas H, Robert C, Lewis K, Protsenko S, et al. Overall survival with first-line atezolizumab in combination with vemurafenib and cobimetinib in BRAF V600 mutation-positive advanced melanoma (IMspire150): second interim analysis of a multicentre, randomised, phase 3 study. Lancet Oncol. 2023;24(1):33–44. 10.1016/s1470-2045(22)00687-8. 36460017 10.1016/S1470-2045(22)00687-8

[402] Menzies AM, Lo SN, Saw RPM, Gonzalez M, Ch’ng S, Nieweg OE, et al. Five-year analysis of neoadjuvant dabrafenib and trametinib for stage III melanoma. Ann Oncol. 2024;35(8):739–46. 10.1016/j.annonc.2024.05.002. 38754780 10.1016/j.annonc.2024.05.002

[403] Kawai K, Hayashi M, Hirota A, Nishioka M, Maesono T, Okano N, et al. P10–6 Report of a case of sigmoid colon cancer simultaneously harboring the BRAF V600E mutation and NTRK3 fusion gene. Ann Oncol. 2022;33. 10.1016/j.annonc.2022.05.246.

[404] Schadendorf D, Hauschild A, Santinami M, Atkinson V, Mandalà M, Chiarion-Sileni V, et al. Patient-reported outcomes in patients with resected, high-risk melanoma with BRAF V600E or BRAF V600K mutations treated with adjuvant dabrafenib plus trametinib (COMBI-AD): a randomised, placebo-controlled, phase 3 trial. Lancet Oncol. 2019;20(5):701–10. 10.1016/s1470-2045(18)30940-9. 30928620 10.1016/S1470-2045(18)30940-9

